# Deconstructing an infamous extinction crisis: Survival of *Partula *species on Moorea and Tahiti

**DOI:** 10.1111/eva.12778

**Published:** 2019-02-27

**Authors:** Amanda E. Haponski, Taehwan Lee, Diarmaid Ó Foighil

**Affiliations:** ^1^ Department of Ecology and Evolutionary Biology and Museum of Zoology University of Michigan Ann Arbor Michigan

**Keywords:** conservation, ddRADseq, extinction, Moorea, *Partula*, phylogenomics, survival, Tahiti

## Abstract

Eleven of eighteen Society Island *Partula* species endemic to the Windward Island subgroup (Moorea and Tahiti) have been extirpated by an ill‐advised biological control program. The conservation status of this critically endangered tree snail radiation is of considerable import, but is clouded by taxonomic uncertainty due to the extensive lack of congruence among species designations, diagnostic morphologies, and molecular markers. Using a combination of museum, captive, and remnant wild snails, we obtained the first high‐resolution nuclear genomic perspective of the evolutionary relationships and survival of fourteen Windward Island *Partula *species, totaling 93 specimens. We analyzed ~1,607–28,194 nuclear genomic loci collected with the double digest restriction‐site associated sequencing method. Results from phylogenomic trees, species estimation, and population assignment tests yielded monophyly of the Windward Island subgroup. Within this group, two well‐supported clades encompassing five species complexes were recovered. Clade 1 was restricted to Tahiti and contained two species complexes: “*P. affinis*” (three species) and “*P. otaheitana*” (five species). Clade 2 occurred on Moorea and on Tahiti and consisted of three species complexes: one Tahitian, “*P. clara*/*P. hyalina*”; the other two, “*P. taeniata*” (three species) and “*P. suturalis*” (six species), Moorean. Our genomic results largely corroborated previous mitochondrial DNA survival estimates for Moorea and Tahiti, with all five species complexes having members surviving in captivity and/or as remnant wild populations, although the details vary in each case. Continued, proactive conservation and management may yet ensure a phylogenetically representative survival of the fabled *Partula* species of Moorea and Tahiti.

## 1 INTRODUCTION

Over the past hundred years, the partulid tree snails of the Society Islands attained scientific prominence as the subject of classic studies in zoology, population biology, and evolutionary genetics (Crampton, 1916, 1932; Johnson, Murray, & Clarke, 1993a; Murray & Clarke, 1980; Murray, Clarke, & Johnson, 1993). They are viewed as a classic example of an adaptive radiation (e.g., Goodacre, 2002; Johnson et al., 1993a; Murray et al., 1993) with species displaying a variety of phenotypes, ecological differentiation, and reproductive isolation across their distribution (Cowie, 1992; Johnson, Murray, & Clarke, 1993b; Murray et al., 1993; Murray, Johnson, & Clarke, 1982).

**Figure 1:**
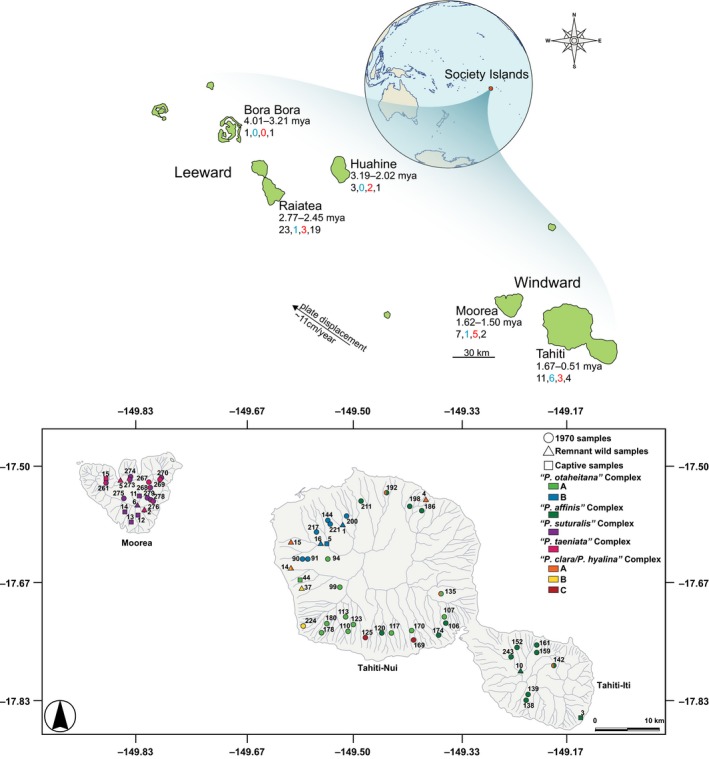
(a) Map of the Society Islands hotspot archipelago showing the Leeward and Windward Island subgroups. The first series of numbers under each island name indicate the estimated geological ages of island strata in millions of years (Duncan, Fisk, White, & Nielsen, 1994; Guillou et al., 2005; Hildenbrand, Gillot, & Le Roy, 2004; Uto et al., 2007). The second series of numbers show (from left to right) the number of endemic *Partula *species recognized for that island, followed by the number that survive in the wild (blue), that survive in captivity (red), and number that are deemed extinct (Gerlach, 2016). (b) Map showing Moorean and Tahitian valley and montane sampling locations. Sites are color‐coded according to results from the phylogenomic trees, Structure, and DAPC analyses (see Figures 3, 4, 5, 6 and Supporting Information Figure S1). Gray lines indicate mountain ridges

However, during the late 20th century, Society Island partulids fell victim to an infamous mass extinction following the deliberate introduction of the alien carnivorous land snail *Euglandina rosea *(Figure 1a; Clarke, Murray, & Johnson, 1984; Cowie, 1992; Gould, 1994; Gerlach, 2016). The rationale for the introduction was a misguided biological control program aimed at another alien mollusk, the giant African land snail, *Lissachatina fulica*, an agricultural pest (Clarke et al., 1984). *Euglandina rosea* was released on Tahiti in 1974, Moorea in 1977, and on other Society Islands from 1980 to 1990s (Coote, 2007). Approximately 51% (*N = *28/55 species) of Society Island partulid species are now considered extinct (Coote & Loève, 2003; Gerlach, 2016), with 96% (27/28 spp.) of those representing taxa from the genus *Partula *(*N = *27/51 spp.). A subset of *Partula *tree snails collectively persists in captivity (*N* = 13 spp.; Figure 1a; Gerlach, 2016; Pearce‐Kelly, Clarke, Walker, & Atkin, 1997) and in the wild in cool, cloud forest montane refuges (*N* = 4, *P. meyeri *on Raiatea and *P. compressa*,* P. laevigata, *and *P. otaheitana *on Tahiti) where *E. rosea* may be less effective (Gerlach, 1994, 2016; Lee et al., 2009; Lee, Burch, Jung et al., 2007; Lee, Meyer, Burch, Pearce‐Kelly, & Ó Foighil, 2008) or as scattered remnant surviving valley populations on Tahiti (*N* = 3, *P. affinis*, *P. clara*, and *P. hyalina*) and Moorea (*P. taeniata*; Coote, 2007; Lee et al., 2009; see Table 1 and Supporting Information Table S1).

**Table 1:** Moorean and Tahitian *Partula *species currently recognized and whether they are extinct or still occur as remnant wild or captive populations based on Gerlach (2016)

Island	Species	Status
Moorea	P. aurantia*	Extinct
	P. exigua*	Extinct
	P. mirabilis*	Captive
	P. mooreana*	Captive
	P. suturalis*	Captive
	P. taeniata*	Remnant wild/captive
	P. tohiveana*	Captive
Tahiti	P. affinis*	Remnant wild/captive
	P. clara*	Remnant wild
	*P. compressa*	Remnant wild
	*P. cytherea*	Extinct
	P. diminuta*	Extinct
	P. hyalina*	Remnant wild/captive
	*P. jackieburchi*	Extinct
	*P. laevigata*	Remnant wild
	P. nodosa*	Captive
	P. otaheitana*	Remnant wild
	P. producta*	Extinct

Note

Those marked with an * are included in this study.

Estimates of the number of Society Island endemic *Partula *species and of their survival have been in considerable flux complicating the conservation status of this critically endangered archipelagic radiation. For instance, one study (Coote & Loève, 2003) recorded 16/58 species surviving, with all 16 surviving in captivity and five of those also surviving in the wild, whereas a more recent taxonomic revision (Gerlach, 2016) respectively listed a total of 18/51 surviving, with five surviving in the wild, three in the wild and in captivity, and 10 in captivity. A persistent issue has been the extensive lack of congruence among taxonomy, morphology, different molecular markers, and degree of reproductive isolation among the species (Clarke, Johnson, Murray, Hewitt, & Wragg, 1996; Gerlach, 2016; Haponski, Lee, & Ó Foighil, 2017; Johnson, Murray, & Clarke, 1986a; Lee, Li, Churchill, & Ó Foighil, 2014; Murray, Stine, & Johnson, 1991), especially for the much better studied species on the Windward Islands of Moorea and Tahiti.

**Figure 2:**
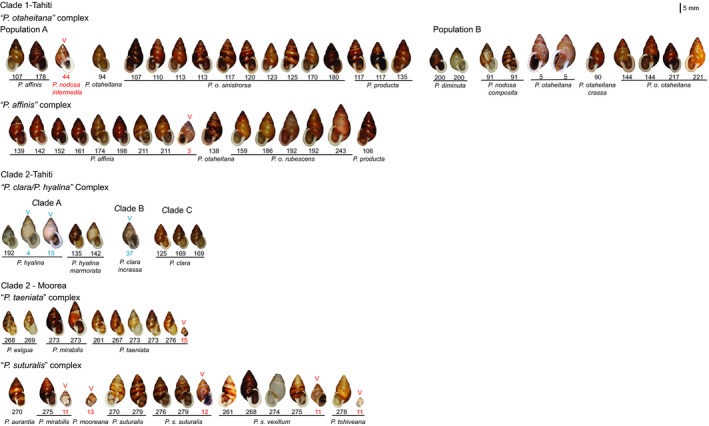
Photographic plate of the Moorean and Tahitian *Partula *species and subspecies genotyped in this study (see Supporting Information Table S1 for detailed site information). Photo credits: J. B. Burch for 1970 museum samples (black text) and A. M. Cacciaglia for remnant wild valley (V, blue) and captive valley (V, red) snails. Note: The captive valley specimens for *P. taeniata*, *P. mirabilis*, *P. mooreana*, and *P. tohiveana *are juvenile snails

Currently, 18 species are recognized from the islands of Moorea (*N* = 7 spp.) and Tahiti (*N* = 11 spp.), with many of these species exhibiting a high degree of overlap in traditional conchological and reproductive anatomical characteristics, with similar forms found in multiple species (see Figure 2; Crampton, 1916, 1932; Johnson et al., 1993b; Murray & Clarke, 1968). Moreover, molecular studies utilizing allozymes, mitochondrial genotypes, nuclear ribosomal sequences, and initial phylogenomic data have consistently failed to recover the Moorean and Tahitian species as monophyletic (Goodacre, 2001, 2002; Haponski et al., 2017; Lee, Burch, Jung et al., 2007; Lee et al., 2014; Murray et al., 1991). Notably, work by B. Clarke, J. Murray, M. Johnson and their associates over a number of decades on Moorea (Clarke et al., 1996; Goodacre, 2001, 2002; Johnson, Clarke, & Murray, 1977; Johnson et al., 1993a; Murray & Clarke, 1980) demonstrated that six of the seven Moorean species formed two species complexes: (a) *P. taeniata *and *P. exigua* and (b) *P. suturalis*, *P. tohiveana*, *P. mooreana*, and *P. aurantia*. The seventh species, *P. mirabilis*, could hybridize with either complex (Murray & Clarke, 1980). The 11 species on Tahiti have not been as well studied as those on Moorea. Much of our understanding stems from molecular studies that relied on mitochondrial (mt) markers and showed extensive poly‐ and paraphyly among the species (Goodacre, 2001, 2002; Lee, Burch, Jung et al., 2007) complicating assessments of survival and conservation management action plans.

The *status quo* taxonomic assessment of survival is that 11 of 18 Moorean and Tahitian *Partula *species are extirpated and six of 18 species are extinct (Table 1; Gerlach, 2016). In contrast, mt phylogenetic analyses of museum, captive, and remnant wild specimens showed much higher survival, with only one major mt clade containing mostly Moorean *P. suturalis *individuals as extinct (Lee et al., 2009; Lee, Burch, Jung et al., 2007). However, the mt results were based on a single molecular marker that is incongruent with nuclear datasets for these taxa (Haponski et al., 2017), a shortcoming common to many other study systems (Wallis et al., 2017). Given the taxonomic uncertainties and the limitations of the mt phylogenies, we still lack a robust understanding of what fraction of the original Windward Islands radiation has persisted. These fundamental gaps in our knowledge significantly impair our ability to not only understand the evolutionary history of these critically endangered taxa but also to design optimal conservation management programs and strategies to aid their survival.

To address these outstanding issues, we generated the first high‐resolution phylogenomic perspective of (a) the evolutionary relationships of Moorean and Tahitian *Partula *species and (b) the fraction of the radiation that has survived. We analyzed ~1,607–28,194 nuclear genomic loci from a combination of museum, captive, and remnant wild specimens which allowed us to compare relationships both pre‐ and post‐extirpation. Compared to taxonomic estimates of survival, our phylogenomic results reveal the presence of five species complexes, all of which remain extant, despite catastrophic population declines.

## 2 MATERIALS AND METHODS

### 2.1 Samples and sampling design

To address the evolutionary relationships and survival of Moorean and Tahitian *Partula *species, we sampled a total of 120 partulid individuals comprising two genera and 31 species. We sequenced 93 specimens representing all seven Moorean *Partula *species (*N* = 32 individuals), and 7/11 Tahitian species (*N* = 61 individuals) sampled from valleys and montane regions across both islands (Figure 1b, Supporting Information Table S1). These specimens characterized a majority of the taxonomic species (14/18; Gerlach, 2016) and all known mt cytochrome *c *oxidase subunit I (COI) clades (Lee, Burch, Coote et al., 2007; Lee et al., 2009, 2014, 2008; Lee, Burch, Jung et al., 2007). Our goal here was to include as many species and sampling locations, but our sampling of the valleys and montane regions was not exhaustive. These specimens were previously analyzed for mt COI (Lee, Burch, Coote et al., 2007; Lee et al., 2009, 2014, 2008; Lee, Burch, Jung et al., 2007) and a subset (*N* = 20 individuals) for double digest restriction‐site associated sequencing (ddRADseq; Haponski et al., 2017). These samples also represented a genomic snap shot both before and after the mass extinction event with 69 specimens collected in 1970 by J.B. Burch and colleagues on both Moorea and Tahiti prior to the introduction of the predator *E. rosea. *These museum specimens were mailed alive to the University of Michigan's Museum of Zoology (UMMZ) in 1970 where foot tissue samples were freeze‐dried and archived at −20°C until their extraction (Lee, Burch, Coote et al., 2007; Lee et al., 2009, 2014, 2008; Lee, Burch, Jung et al., 2007). The captive (*N* = 11 individuals), and remnant wild (*N* = 13 individuals) alcohol specimens were collected from 1994–1995, 1999, 2001–2006, and 2009 as whole snails or as foot biopsies, preserved in 95% ethanol and then archived at the UMMZ (Supporting Information Table S1).

We also sampled several outgroup species (Supporting Information Table S1) representing a range of closely to more distantly related taxa to determine the evolutionary relationships and survival of Moorean and Tahitian *Partula* species. These included ten congeners from the adjacent Leeward Island subgroup (Bora Bora, Huahine, and Raitea) and four Western Pacific congeners, the sister clade of Society Island *Partula *species (Lee et al., 2014). Lastly, we also included three Society Island *Samoana *species, the sister genus of *Partula*. The taxonomy used here complies with the most recent Partulidae revision by Gerlach (2016), with the exception of *Partula incrassa*. We retained its original name, *P. exigua*, due to the recent clarification of the phylogenomic relationships of *P. clara incrassa* and its congeners (Haponski et al., 2017).

### 2.2 ddRADseq data collection and bioinformatics

The DNA of the 120 partulid individuals genotyped in this study was previously extracted using a Qiagen DNEasy Kit (Qiagen, Valencia, CA, USA) or an E.Z.N.A. Mollusk DNA kit (Omega Bio‐Tek, Norcross, GA; Lee, Burch, Coote et al., 2007; Lee et al., 2009, 2014, 2008; Lee, Burch, Jung et al., 2007) and then stored at −80°C. The quantity of these archived DNA extractions was assessed using a Qubit 2.0 Fluorometer (Life Technologies, Carlsbad, CA, USA) housed at University of Michigan's Genomic Diversity Laboratory (GDL; http://www.lsa.umich.edu/gdl/samplequality/default.asp). We targeted 200 ng of DNA for library preparation, and any individuals with DNA quantities less than this were re‐extracted using an E.Z.N.A. Mollusk DNA kit following manufacturer's instructions. ddRADseq libraries then were prepared and followed the protocols of Peterson, Weber, Kay, Fisher, and Hoekstra (2012).

Genomic DNA was digested using the restriction enzymes Eco‐RI‐HF and MspI (New England Biolabs, Ipswich, MA, USA), and a 294–394 bp fragment (excluding Illumina adapters) was targeted for sequencing using a Pippen Prep (Sage Science, Beverly, MA, USA) following the manufacturer's instructions. Prepared ddRADseq libraries then were submitted to the University of Michigan's DNA sequencing core (http://medicine.umich.edu/medschool/research/office-research/biomedical-research-core-facilities/dna-sequencing) and run in three different lanes using 100 or 150 bp paired‐end sequencing on an Illumina HiSeq 2500. Three control individuals (Moorean *P. taeniata *NUCM1 and Tahitian *P. clara incrassa *PCTI and *P. hyalina* PHTH2) were run in every lane to ensure no lane effects in downstream data processing.

Sequence quality first was assessed using Fastqc v.0.11.5 (Andrews, 2018) and showed the presence of Illumina adapters in one of three sequencing lanes and Phred quality scores ranging from 14 to 38. Raw sequences then were deposited on the Flux high computing cluster at the University of Michigan's Center for Advanced Computing for further processing and analyses.

The alignment‐clustering algorithm in ipyrad v.0.7.17 (Eaton, 2014; Eaton & Overcast, 2018) was used to process and identify homologous ddRADseq tags with parameters modified to reflect the Fastqc results. In comparison with other methods, ipyrad allowed aligned tags to include insertions and deletions, which can be especially beneficial for studies with broad taxonomic coverage (Eaton, 2014) as done here with Society Island *Partula, *western *Partula, *and *Samoana* specimens. Illumina sequences first were demultiplexed by sorting reads by barcode, allowing no barcode mis‐matches (parameter 15 setting 0), a maximum of five low quality bases (parameter 9) and merged reads then detected in ipyrad. Restriction sites, barcodes, and Illumina adapters (based on Fastqc results; parameter 16 setting 2) then were trimmed from raw sequence reads, and bases with low quality scores (Phred‐score <20, parameter 10 setting 33) replaced with *N. *Sequences with >5 *N*s (parameter 19) were discarded. Reads then were clustered and aligned within each individual sample at three different similarity thresholds, 85, 90, and 95%. Clusters of aligned loci with a depth of coverage <6 (parameters 11 and 12) were discarded. Remaining reads then were clustered and aligned across individuals, filtered for paralogs, and finally concatenated into consensus loci at 85, 90, and 95% similarity *de novo* in ipyrad. We also varied the minimum number of individuals required for a consensus locus to be retained in the final dataset with a final filtering step that removed any consensus loci not recovered across (a) 75% (*N* = 90 individuals), (b) 50% (*N* = 60 individuals), or (c) 25% (*N* = 30 individuals) of individuals. Output files for these final nine concatenated datasets were exported for further downstream analysis and file conversion where needed.

### 2.3 Phylogenomic analyses of Moorean and Tahitian clades

**Figure 3:**
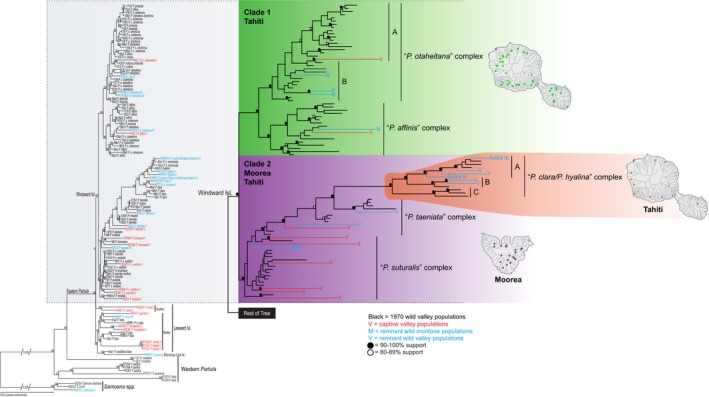
Maximum likelihood phylogenomic tree depicting relationships among the 14 Windward Island *Partula* species for the 2,169 locus 90% similarity threshold clustering across 75% of individuals (see Supporting Information Figure S1 for full tree). Tree was rooted with three species of Society Islands *Samoana, *the sister genus of *Partula*. Values on tree nodes indicate maximum likelihood bootstrap supports. Individuals are identified as 1970 wild (black), remnant valley (V, blue) or montane (M, blue), or captive (C, red) populations. A * denotes the remnant montane *P. taeniata *individual (MTOI2) from Clade 2 (see Section 44). Maps show sampling locations for the two major Moorean and Tahitian clades (see Figure 1b; Supporting Information Table S1 for site details)

To determine phylogenomic relationships among the 93 Moorean and Tahitian specimens, we analyzed the nine concatenated ddRADseq alignment files using maximum likelihood in RAxML v8.2.8 (Stamatakis, 2014). Analyses utilized the general time reversible model (Lanave, Preparata, Saccone, & Serio, 1984) and included invariable sites and a gamma distribution. Support for nodes was determined from 100 fast parametric bootstrap replications. The nine resulting trees showed congruent phylogenomic relationships and similar support values among major Society Island clades (see Figure 3 and Supporting Information Figure S1). Since these relationships were robust across the nine datasets, we then selected the 90% similarity threshold with 75% of individuals included (90‐75 hereafter) for all remaining analyses as it had an intermediate number of loci (2,169), intermediate similarity threshold (90%), and had at least 90/120 individuals (75%) present in every locus.

In addition to the RAxML analyses, we also conducted a Bayesian analysis on the concatenated 90‐75 alignment in the parallel version of MrBayes v3.2.6 (Ronquist et al., ). Bayesian analyses also included the general time reversible model with invariable sites and a gamma distribution and used a Metropolis‐coupled Markov chain Monte Carlo (MC^3^) approach and ran for 4,000,000 generations, with sampling every 100. Two analyses were performed each with four separate chains run simultaneously. Stationarity and burn‐in period for the MC^3^ were determined by plotting log likelihood values for each generation. The first 25% of the generations, trees, and parameter values sampled were discarded as burn‐in. The runs were considered to have reached convergence when the average split standard deviation was <0.01, the potential scale reduction factor was between 1.00 and 1.02, and log likelihood plots appeared as white noise (Ronquist, Huelsenbeck, & Teslenko, 2011). A 50% majority rule consensus tree was based on the remaining generations, whose branch support was determined from the posterior probability distribution (Holder & Lewis, 2003) in MrBayes.

### 2.4 Species estimation of Moorean and Tahitian clades

Phylogenies estimated from concatenated datasets may mislead when loci distributed across the genome have different evolutionary histories due to processes such as hybridization, incomplete lineage sorting (ILS), and gene duplication/loss (Chou et al., 2015; Maddison, 1997), especially in taxa that have undergone rapid radiations (Mirarab & Warnow, 2015). This is a potential concern regarding the relatively well‐studied *Partula* species of Moorea that show evidence of extensive hybridization and of rapid radiation (Chiba & Cowie, 2016; Murray & Clarke, 1980; Murray et al., 1993). To address this issue, we also constructed a phylogeny using the coalescent‐based approach in SVDquartets (Chifman & Kubatko, 2014) as implemented in PAUP* v4.0a157 (Swofford, 2002).

The program SVDquartets takes multi‐locus, unlinked single nucleotide polymorphisms (SNPs), and infers quartet trees from all subsets of four samples. These are then scored, and valid inferred splits based on these scores are combined into a tree using a quartet assembly method. We used the python code provided by Bongaerts (2018; vcf_single_snp.py) to first convert our variant call format (VCF) file containing all SNPs for the 90‐75 dataset to randomly select a single SNP per locus (total of 2,169 SNPs). We then used PGDSpider v2.1.1.0 (Lischer & Excoffier, 2012) to convert this single SNP VCF file to a nexus file for input into PAUP*. SVDquartets estimated that there were 4,159,122 quartets present in our 90‐75 2,169 single SNP dataset. Due to the large number of quartets and taxa, we analyzed a random subset of 1,000,000 quartets that represented ~25% of the distinct quartets. We determined support of the inferred relationships with 100 bootstrap replicates. Trees were assembled using the QFM quartet‐based phylogeny reconstruction algorithm. We ran the SVDquartets analysis in two ways: (a) grouping individuals into the clades recovered in the RAxML and Bayesian trees and (b) no groupings specified.

### 2.5 Population genomic analyses Moorean and Tahitian species

To test for genetic structuring within the recovered Moorean and Tahitian clades, we used two different methods: Bayesian based Structure v2.3.4 (Hubisz, Falush, Stephens, & Pritchard, 2009; Pritchard, Stephens, & Donnelly, 2000) and Discriminant Analysis of Principal Components (DAPC; Jombart, Devillard, & Balloux, 2010) analyses. As input for the former method, we converted the 90‐75 single SNP dataset used for the SVDquartets analysis to the Structure format using PGDSpider keeping only Moorean and Tahitian individuals (*N* = 93 individuals) and removing any loci consisting entirely of missing data or non‐polymorphic SNPs. The final dataset contained 2,167 SNPs.

We ran Structure iteratively (see Massatti & Knowles, 2014; Thomaz, Malabarba, & Knowles, 2017) to fully explore population sub‐structuring within the 90‐75 Moorean and Tahitian dataset. Structure was initially run including all 93 Moorean and Tahitian individuals with parameters set to defaults and *K*‐values varying from one to seven (the number of well‐supported clades in the phylogenomic trees (5) plus two). Subsets of the data that corresponded to the respective genetic clusters identified in the initial runs then were run with the number of *K*‐values ranging from *K = *1 to the number of well‐supported clades on the tree plus one. In total, five Structure analyses were performed: the full dataset of 93 samples, within each of the recovered Structure clusters, and for the two major clades evident from the phylogenomic trees.

For each Structure run, 10 independent runs for each *K* were performed with a burn‐in length of 150,000 replicates followed by 500,000 generations. Stationarity and the optimal *K* were assessed using the Δ*K *method of Evanno, Regnaut, and Goudet (2005) in the web‐based Structure Harvester (Earl & vonHoldt, 2012) and posterior probabilities (Pritchard et al., 2000) in Clumpak v1.1 (Kopelman, Mayzel, Jakobsson, Rosenberg, & Mayrose, 2015). Results from Structure runs then were visualized using Distruct v1.1 (Rosenberg, 2004) in Clumpak.

Discriminant Analysis of Principal Components is a multivariate clustering method that is more likely to infer the true number of subpopulations from hierarchical data as compared to Structure when large SNP datasets are used (Jombart et al., 2010). We implemented it via the *adegenet* package (Jombart, 2008) in R v3.3.3 (R Development Core Team, 2017). DAPC analyses also followed an iterative approach with the initial run consisting of all 93 Moorean and Tahitian *Partula *specimens, and then two subsequent runs within the recovered Clade 1‐Tahiti and Clade 2‐Tahiti clusters. The 90‐75 single SNP dataset was converted to the Genepop (Rousset, 2008) format in PGDSpider for input into DAPC.

Prior to DAPC analysis, we used the *K*‐means clustering of principal components to identify groups of individuals by maximizing the separation between groups while minimizing variation within the groups (Jombart et al., 2010). We determined the optimal number of principal components to maintain using the command optim.a.score, which showed nine for the total dataset, and two each for the Clade 1‐Tahiti and Clade 2‐Tahiti clusters (Supporting Information Figure S2). The Bayesian Information Criterion (BIC) showed the most likely number of clusters in the full dataset to be six. However, BIC was uninformative for the Clade 1‐Tahiti and Clade 2‐Tahiti analyses (Supporting Information Figure S2). We chose three for Clade 1‐Tahiti and three for Clade 2‐Tahiti based on the results from the phylogenomic trees and Structure analyses. For the Clade 1‐Tahiti cluster, the selection of three clusters did not correspond to the three Structure clusters recovered so we increased the possible number of clusters to four. Relationships among the clusters for each analysis were determined by plotting the first two principal components of the DAPC. Assignment accuracy for each individual also was assessed in DAPC.

We also tested for admixture and gene flow among the Moorean and Tahitian clades using the Structure assignments and threepop (*f*_3_) tests of Reich, Thangaraj, Patterson, Price, and Singh (2009) in the program TreeMix v1.13 (Pickrell & Pritchard, 2012). Briefly, the threepop test is formulated as *f*_3_(A; B, C) and compares whether population A has inherited a history of admixture using populations B and C as reference points (see Reich et al., 2009). A significantly negative value implies that population A is admixed (see Reich et al., 2009; Pickrell & Pritchard, 2012). The 93 Moorean and Tahitian specimens were grouped based on the results from the phylogenomic trees, Structure, and DAPC results. Input files were created using the python code vcf2treemix.py (Silva, 2018), and all possible *f*_3_ comparisons were run using blocks of 100 SNPs. Significance of *Z*‐score values then was assessed in R.

## 3 RESULTS

### 3.1 Summary of ddRADseq data

Illumina sequencing returned raw read numbers ranging from 165,507 to 5,705,274 across the 120 partulid samples, with eleven individuals having fewer than 1,000,000 reads (Supporting Information Table S2). Clustering at 85%, 90%, and 95% similarity thresholds resulted in congruent numbers of loci across the 120 individuals that passed quality filtering. The overall number of loci increased across the three similarity thresholds presumably due to homologous reads splitting into multiple loci at high stringency (90% and 95%) thresholds. The mean coverage depth of loci ranged from 9.0 to 113.8 for the 85% threshold, 9.1 to 112.0 for 90%, and 8.8 to 108.1 for 95%, with *P. pacifica *(PTUR7), an outgroup sample, having the lowest coverage and *P. hyalina* (PHTH2) having the highest coverage (Supporting Information Table S2).

We identified 1,607–28,194 nuclear genomic loci across the nine ddRADseq datasets for Society Island *Partula *species. The number of loci in the final datasets increased as the minimum number of individuals (75%, 50%, 25%) required for retaining a locus decreased. For the 85% threshold across all 75% of the 120 samples (*N* = 90 individuals), 1,607 loci were recovered in the final ddRADseq dataset whereas the number of loci for the 90% and 95% levels increased to 2,169 and 2,455, respectively. Decreasing the minimum taxon coverage from 75% to 50% (*N* = 60 individuals) resulted in a fivefold increase in the number of loci: 8,381 for 85%, 11,026 for 90%, and 11,506 for 95% threshold datasets. The number of loci also increased when only 25% (*N* = 30 individuals) of individuals were required to retain a locus (85%—18,154, 90%—23,195, 95%—28,194). Higher numbers of loci were recovered in the Society Island *Partula *individuals compared to outgroup samples (Supporting Information Table S3). Within the Moorean and Tahitian individuals of interest, similar numbers of loci were recovered across all 93 samples across the different similarity thresholds and taxon coverages (Supporting Information Table S3).

For each Illumina sequencing run, we included three control individuals: Moorean *P. taeniata *NUCM1 and Tahitian *P. clara incrassa *PCTI and *P. hyalina* PHTH2. The resulting reads for each of the three individuals clustered together in every analysis with 100% bootstrap support regardless of similarity threshold or number of individuals required for retaining a locus (data not shown) indicating there were no sequencing lane effects on clustering across individuals.

### 3.2 Moorean and Tahitian *Partula *phylogenomic clades

**Figure 4:**
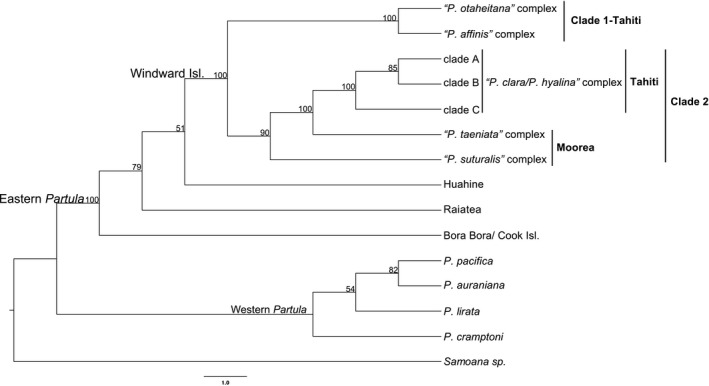
Species tree estimation of Moorean and Tahitian *Partula *clades for individuals grouped by species complex following the full phylogenomic tree, Structure, and Discriminant Analysis of Principal Components analyses (see Figures 3, 4, 5, 6 and Supporting Information Figure S1). The 2,169 locus 90% similarity threshold clustering across 75% of individuals dataset was analyzed with SVDquartets (Chifman & Kubatko, 2014) as implemented in PAUP* (Swofford, 2002). Bootstrap supports are indicated for nodes with values >50%. Tree was rooted with three species of Society Islands *Samoana *(denoted *Samoana *sp*.*)*, *the sister genus of *Partula. *All Society Islands *Partula *individuals were included in the analysis and are identified by their island of origin.

The 14 sampled Moorean and Tahitian *Partula* species (*N* = 93 individuals) were consistently recovered in a single well‐supported monophyletic Windward Island clade irrespective of the different clustering thresholds (85%, 90%, 95%) and minimum taxon coverages (75%, 50%, 25%) used to build the phylogenomic trees (Figure 3 and Supporting Information Figure S1) or groupings in the species estimation method (Figure 4 and Supporting Information Figure S3). The Windward Island clade's sister relationship to Leeward Islands congeners was not clearly resolved by the species estimation method (Figure 4 and Supporting Information Figure S3) or by the ddRAD datasets: 5/9 identifying Huahine congeners and 4/9 Raiatean congeners (Figure 3 and Supporting Information Figure S1). One interesting detail regarding our Leeward Island partulid results was the well‐supported sister relationship recovered for the now extinct Bora Bora endemic *P. lutea* and the Rarotonga (Cook Islands) endemic *P. assimilis* (Figures 3 and 4, Supporting Information Figures S1 and S3).

Within the Windward Island clade, the phylogenomic and species estimation trees did not corroborate the currently described 14 *Partula *species. The phylogenomic results recovered two well‐supported monophyletic clades (Figures 3 and 4, Supporting Information Figures S1 and S3). Clade 1 (green in Figures 3 and 4) was restricted to Tahiti and contained five of the seven Tahitian species: *P. affinis*, *P. diminuta*, *P. nodosa*, *P. otaheitana *and its subspecies, and *P. producta* (Figures 3 and 4, Supporting Information Figures S1 and S3; Supporting Information Table S1). Clade 2 occurred on both Moorea and Tahiti and contained all seven of the Moorean species: *P. aurantia*, *P. exigua*, *P. mirabilis*, *P. mooreana*, *P. suturalis *and its subspecies, *P. taeniata*, and *P. tohiveana *(purple in Figures 3 and 4) and the two remaining Tahitian species *P. clara *and *P. hyalina *(orange in Figures 3 and 4). The Tahitian portion of Clade 2 (*P. clara *and *P. hyalina*) received robust support in the phylogenomic and species estimation trees (100% in all trees) but were nested within a clade containing the Moorean species *P. exigua*, *P. mirabilis*, and *P. taeniata *(Figures 3 and 4, Supporting Information Figures S1 and S3). Regardless of the method used, the 14 morphological species appeared as para‐ and polyphyletic in all phylogenomic and species estimation trees (Figures 3 and 4, Supporting Information Figures S1 and S3).

### 3.3 Population genomic structure within Moorean and Tahitian clades

**Figure 5:**
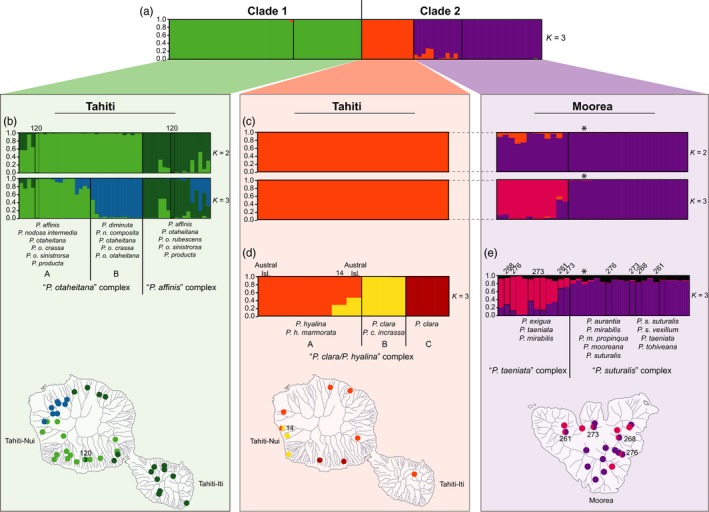
Structure bar graphs showing the most likely assignment of individuals from (a) all 93 Moorean and Tahitian *Partula *individuals, (b) Tahitian Clade 1, (c) Moorean and Tahitian Clade 2, (d) Tahitian “*P. clara*/*P. hyalina*” species complex, and (e) Moorean “*P. taeniata*” and “*P. suturalis*” species complexes based on the Δ*K *method of Evanno et al. (2005) in Structure Harvester (Earl & vonHoldt, 2012; Supporting Information Figure S4)*. *Structure analyses used a single SNP per locus (totaling 2,167 SNPs) for each individual, and each vertical bar represents an individual snail. Maps show sampling locations where the clades occur on each of their respective islands. Labels on Structure graphs include major species complex identification (e.g., “*P. otaheitana*” complex), site numbers where the different populations/clades occur in sympatry, and constituent species/subspecies from the current taxonomy (see Supporting Information Tables S1 and S4). A ^*^ denotes the remnant montane *P. taeniata *(MTO12) individual from Clade 2 (see Section 44)

The 93 Moorean and Tahitian individuals assigned highly (~72%–100%; Supporting Information Table S4) to three population groups that paralleled the highly supported clades in the phylogenomic and species estimation trees, individuals from Clade 1 (green) and those from Clade 2 (purple, orange; Figure 5). In the analysis of 93 individuals, Clade 2 clustered into two population groups corresponding to locations of the *Partula *samples on either the island of Moorea (purple) or Tahiti (orange; Figure 5a and Supporting Information Figure S4a; Supporting Information Table S4a).

Within Clade 1 (Tahiti), Structure analyses recovered two clusters, a “*P. otaheitana*” species complex and “*P. affinis*” species complex (Figure 5b and Supporting Information Figure S4b), that each showed high self‐assignment (48%–100%; Supporting Information Table S4b) and were well supported in the phylogenomic trees (Figure 3 and Supporting Information Figure S1). The “*P. otaheitana*” species complex contained individuals described as *P. affinis*, *P. nodosa composita*, *P. n. intermedia*, *P. otaheitana*, *P. o. crassa*, *P. o. otaheitana*, *P. o. sinistrorsa*, and *P. producta *and the “*P. affinis*” species complex consisted of specimens identified as *P. affinis*, *P. otaheitana*, *P. o. rubescens*, *P. o. sinistrorsa*, and *P. producta *(Figure 5b; Supporting Information Tables S1 and S4b)*. *Within the “*P. otaheitana*” species complex, Structure analyses recovered additional genetic sub‐structuring with two population groups “A” (light green, 60%–99%) and “B” (dark blue, 54%–99%), each with high self‐assignment values (Figure 5b and Supporting Information Figure S4b; Supporting Information Table S4b), but this was not supported by the phylogenomic trees (Figure 3 and Supporting Information Figure S1). The three within‐Tahitian Clade 1 clusters (“*P. otaheitana*” A and B and “*P. affinis*”) had largely distinct distributions across Tahitian valleys (Figure 5b): “*P. affinis*” (dark green) genotypes were largely absent from the south and the west of Tahiti‐Nui where the two “*P. otaheitana*” clusters dominated, one in the northwest (dark blue) and the other in the south (light green).

When the Clade 2 individuals were run independently in our hierarchical Structure analysis, the results clearly depicted the separation of the Moorean (purple) and Tahitian (orange) portions of the clade with high assignment values (72%–100%; Figure 5a,c, and Supporting Information Figure S4c; Supporting Information Table S4a,c), despite the former being phylogenetically nested with Moorean individuals (Figures 3 and 4, Supporting Information Figures S1 and S3). The Structure runs supported recognition of three species complexes: Tahitian “*P. clara*/*P. hyalina*” and Moorean “*P. taeniata*” and “*P. suturalis.*” Within the Tahitian “*P. clara*/*P. hyalina*” species complex (orange), snails showed high self‐assignment probabilities (53%–100%; Supporting Information Table S4d) to three different clusters (A, B, C; Figure 5d and Supporting Information Figure S4d), all three with 100% support in our phylogenomic trees. Austral Island *P. hyalina *individuals nested within “*P. clara*/*P. hyalina*” A (orange; Figure 5d). Although our Tahitian portion of Clade 2 sampling is modest (*N* = 13 individuals), these clusters appeared to have parallel patterns of distributions to those of Tahitian Clade 1 (Figure 5d), with clade A occurring in the north and east of Tahiti‐Nui and in Tahiti‐Iti, clade B in the west, and clade C in the south (Figure 5d).

The Moorean portion of Clade 2 assigned to two different gene pools, a “*P. taeniata*” species complex (pink) and “*P. suturalis*” species complex (purple; Figure 5e, Supporting Information Table S4e), in Structure analyses. The “*P. taeniata*” species complex contained three of the seven Moorean species: *P. exigua*, *P. mirabilis*, and *P. taeniata *(see Figures 3 and 5e, and Supporting Information Figure S1). The “*P. suturalis*” species complex also contained *P. mirabilis *and a single *P. taeniata *(MTO12) and the remaining Moorean species: *P. aurantia, P. mooreana*, *P. suturalis *and its subspecies, and *P. tohiveana*.

**Figure 6:**
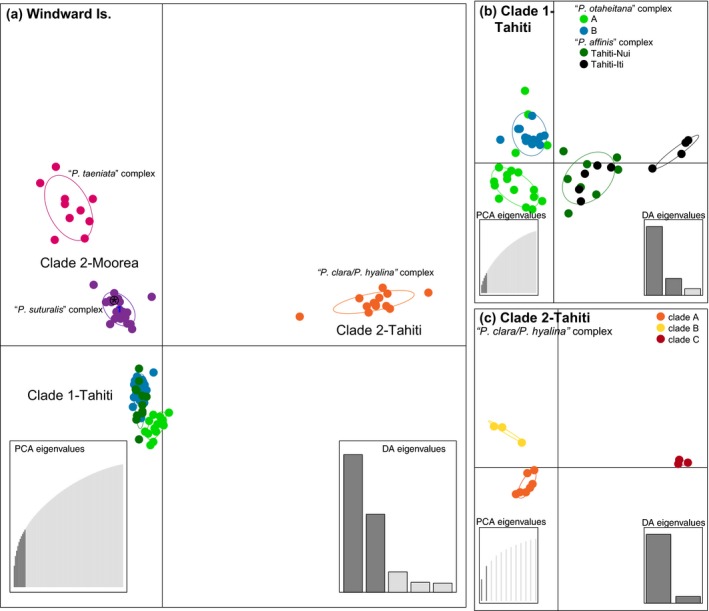
Results from the Discriminant Analysis of Principal Components (DAPC; Jombart et al., 2010) for (a) Windward Islands *Partula*, (b) within Tahitian Clade 1, and (c) within the Tahitian portion of Clade 2. In each of the scatter plots, individuals are represented as dots with 95% confidence intervals surrounding them. Clusters are color‐coded to match those recovered by the Structure analyses (Figure 5). A * denotes the remnant montane *P. taeniata* (MTO12) individual from Clade 2 (see Section 4)

The DAPC analyses were largely congruent with results from the Structure analyses. The BIC chart (Supporting Information Figure S2) showed the most likely number of clusters for the full dataset with all 93 Moorean and Tahitian individuals to be six. These corresponded to the two Moorean species complexes, “*P. suturalis*” (purple) and “*P. taeniata*” (pink) from Clade 2, the Tahitian portion of Clade 2 containing *P. clara *and *P. hyalina *(orange), and three clusters within Tahitian Clade 1 corresponding to the “*P. otaheitana*” (light green and dark blue) and “*P. affinis*” (dark green) species complexes (Figure 6a). The three clusters within Tahitian Clade 1 showed little separation in the overall analysis (Figure 6a); however, when DAPC was run iteratively, they showed greater genetic distinctiveness (Figure 6b) breaking into *“P. otaheitana*” A (light green) and B (dark blue) and “*P. affinis*” (dark green; Figure 6b). The DAPC also identified an additional cluster comprising samples from the “*P. affinis*” species complex that were sampled from Tahiti‐Iti (black; Figure 6b), showing additional geographic variation across the island not recovered in the Structure analysis. The DAPC also recovered three distinct genetic clusters within the Tahitian portion of Clade 2: “*P. clara*/*P. hyalina*” A (orange), B (yellow), and C (red; Figure 6c) matching the relationships in the phylogenomic trees (Figure 3 and Supporting Information Figure S1) and Structure analyses (Figure 5).

### 3.4 Admixture and geneflow among Moorean and Tahitian clades

We found evidence for admixture between Moorean and Tahitian *Partula *clades occurring both within and between the two islands in the Structure and threepop (*f*_3_) tests (Figure 5a; Supporting Information Tables S4 and S5). Between the two islands, Structure analyses indicated admixture between the Moorean “*P. taeniata*” species complex (pink) and Tahitian “*P. clara*/*P. hyalina*” (orange) portion of Clade 2, with individuals identified as *P. exigua *(51a1, 132b1) and *P. taeniata *(130c1, 130d1, 131b1, PS136a1, M10, NUCM1, PHAU, TAEH1) having from 2% (130c1) to 28% (51a1) assignment to the Tahitian portion of Clade 2 (orange) when all 93 Moorean and Tahitian *Partula *individuals were included in the analysis (Figure 5a; Supporting Information Table S4a). When the dataset was reduced to include only Clade 2 snails (orange, purple, and pink; Figure 5c; Supporting Information Table S4c), this assignment decreased and showed only three individuals of *P. taeniata *(M10, PHAU, and TAEH1) having 1%–7% assignment to the Tahitian portion of Clade 2 (orange; Figure 5c; Supporting Information Table S4c). The *f*_3_ test also indicated admixture between the Moorean “*P. taeniata*” species complex and the Tahitian portion of Clade 2 identifying that the admixture was from individuals in “*P. clara*/*P. hyalina*” clade C (Supporting Information Table S5). In addition, a Moorean *P. mirabilis *PM67a1 (Clade 2, purple) snail had a ~3% assignment to Tahitian Clade 1 in the Structure analyses (Figure 5a; Supporting Information Table S4a). Likewise, individuals from the “*P. affinis*” species complex (Tahitian Clade 1) and the Moorean “*P. suturalis*” species complex (Clade 2) showed evidence of admixture in the *f*_3_ tests (Supporting Information Table S5).

There was limited evidence of admixture between *Partula *snails from Clades 1 and 2 within the island of Tahiti in both the Structure and *f*_3_ tests. Structure showed a Tahitian specimen identified as *P. diminuta* 54d2 from the “*P. otaheitana*” species complex (Tahitian Clade 1, light green) had ~5% Structure assignment to the Tahitian portion of Clade 2 “*P. clara*/*P. hyalina*” species complex (orange; Figure 5a; Supporting Information Table S4a). The *f*_3_ test also indicated admixture between Clade 2 individuals comprising “*P. clara*/*P. hyalina*” clade C and the “*P. otaheitana*” and “*P. affinis*” species complexes from Tahitian Clade 1 (Supporting Information Table S5). Within the island of Tahiti, there was evidence of admixture among the three Clade 1 genomic groups “*P. otaheitana*” clades A and B and “*P. affinis*” with some “*P. affinis*” individuals having as high as ~50/50 assignment (80a1 and 97b1) to *“P. otaheitana*” A (Figure 5; Supporting Information Table S4b). Tahiti Clade 2 had little evidence of admixture among individuals with the exception of two *P. hyalina *individuals (PHTM and PHRM1) that assigned ~29%–47% to “*P. clara*/*P. hyalina*” clade B (Figure 5; Supporting Information Table S4d).

Within the island of Moorea (Clade 2), there was evidence for a gradient of admixture between the “*P. taeniata*” snails (pink) from the northwestern portion of the island having the highest assignments (Figure 5e) to “*P. suturalis*” (purple) whereas those with the lowest assignment were found in the eastern portion of the island. “*Partula taeniata*” individuals from site 273 (North‐Central Moorea; Figure 5e) showed the most variability with assignments ranging from ~13% to 72% to “*P. suturalis*” (Supporting Information Table S4d). Two Moorean snails, a *P. exigua* (individual 51a1) from Faamaariri valley (site 269; eastern Moorea) and *P. taeniata *(PHAU) from Haumi valley (site 2; southeastern Moorea), exhibited the lowest genetic contribution from the “*P. suturalis*” species complex (~3%; Figure 5e).

## 4 DISCUSSION

The 14 Moorean and Tahitian *Partula* species analyzed here formed a single well‐supported Windward Island clade (Moorea and Tahiti) with Leeward Island (Bora Bora, Huahine, and Raiatea) taxa as the sister group. In contrast, the earlier mt studies found extensive polyphyly with poor nodal support among Windward and Leeward Island *Partula *clades (Lee et al., 2009, 2014; Lee, Burch, Jung et al., 2007). Our phylogenomic results were broadly consistent with Progression Rule expectations and likely island colonization patterns (see Funk & Wagner, 1995), with the older Leeward islands (Huahine or Raiatea) forming the sister group of the younger Windward Island clade. Likewise, within the Windward Islands the topology of the major clade occurring on both Moorea and Tahiti (Clade 2) is consistent with a colonization from the older island of Moorea to the youngest island Tahiti.

Our phylogenomic analyses also corroborated previous hypotheses for relationships of Society Island *Partula *to other island clades. We recovered a close association of *P. lutea *from Bora Bora with *P. assimilis *from Rarotonga, part of the Cook Islands (Figures 3 and 4, Supporting Information Figures S1 and S3), with *P. assimilis* likely representing a founder lineage from Bora Bora to the Cook Islands similar to previous results using allozymes (Johnson et al.1986a) and nuclear ribosomal sequences (Lee et al., 2014). Additionally, the phylogenomic results supported previous hypotheses of prehistoric Polynesians introducing Tahitian *P. hyalina *to the Austral and Cook Islands (Lee, Burch, Coote et al., 2007), with Austral Island *P. hyalina *individuals nested within our “*P. clara*/*P. hyalina*” species complex (Figures 3, 4, 5, 6 and Supporting Information Figure S1).

The seven Tahitian *Partula *species sampled here were distributed between two phylogenomically distinct clades with non‐overlapping taxonomic compositions (Figures 3, 4, 5, 6 and Supporting Information Figure S1). The five Clade 1 species (*P. affinis*, *P. diminuta*, *P. nodosa, P. otaheitana *and its subspecies, and *P. producta*) are distinguished primarily by relatively modest conchological features (Crampton, 1916; Gerlach, 2016; Figure 2). A century ago, Clade 1 taxa collectively dominated Tahitian valley populations, typically comprising >95% of the tree snails in individual valleys (Bick, Ó Foighil, & Coote, 2016; Crampton, 1916). The two Tahitian Clade 2 species (*P. clara *and *P. hyalina*) were widely distributed in Tahitian valleys but typically represented <5% of individual valley partulid populations (Bick et al., 2016; Crampton, 1916). Taxa from Clades 1 and 2 occurred in sympatry throughout Tahiti's valleys (Crampton, 1916), but we observed little to no evidence for introgression among them either in this study (Figure 5; Supporting Information Table S5), or in earlier mt phylogenies (Lee, Burch, Jung et al., 2007; Lee et al., 2014).

The two Tahitian genomic clades showed a high degree of genetic structuring across the island of Tahiti that corresponded to the locations of the island's mountain ridge, valley, and rain shadow distributions (Hildenbrand, Gillot, & Marlin, 2008; Pasturel, 1993). “*P. otaheitana*” populations A (light green) and B (dark blue) and “*P. clara*/*P. hyalina*” clades B and C were located on two different mountain ridge systems corresponding to the main (northwestern Tahiti‐Nui) or secondary (southern Tahiti‐Nui) volcanic shield (Hildenbrand et al., 2008). Samples representing the “*P. affinis*” species complex (dark green) and “*P. clara*/*P. hyalina*” clade A (orange) were located in areas with the highest precipitation (see Pasturel, 1993) whereas “*P. otaheitana*” and “*P. clara*/*P. hyalina*” clades B (yellow) and C (red) tended to be in dryer regions (Figure 5).

The genetic structuring across the island was more pronounced in Clade 2 (“*P. clara*/*P. hyalina*”; Figure 5), whose distribution was restricted to Tahitian valleys (Gerlach, 2016) than in Tahitian Clade 1 that had both valley (extirpated) and montane (surviving) populations. Clade 1 individuals corresponding to the “*P. affinis*” and “*P. otaheitana*” species complexes had some individuals that assigned as much as 50/50 to both complexes (Figure 5b). This apparent admixture may reflect the ability of individuals in the “*P. affinis*” and “*P. otaheitana*” species complexes to cross montane ridge systems unlike their valley restricted congeners *P. clara *and *P. hyalina. *The DAPC analysis (Figure 6b) showed a subset of “*P. affinis*” individuals from Tahiti‐Iti, the youngest part of the island (Hildenbrand et al., 2008), clustered separately from “*P. affinis*” snails on Tahiti‐Nui. However, further sampling is necessary to determine the significance of this result.

A recent taxonomic revision (Gerlach, 2016) recognized four additional Tahitian *Partula *species not present in our analyses. We lacked identified samples for genotyping because they were described/recognized subsequent to the Tahitian valley museum collections (*P. jackieburchi*) and most occur in montane habitats not sampled by Burch and colleagues (*P. compressa*, *P. cytherea*, and *P. laevigata*). *Partula jackieburchi* is likely a member of Clade 1 (Figure 3) because it is indistinguishable from *P. otaheitana* and *P. affinis* for both allozyme and mitochondrial DNA markers (Johnson et al.1993a; Murray et al., 1991). The genomic distinctiveness of *P. compressa*, *P. cytherea*, and *P. laevigata* remains to be determined, but they are phenotypically close to *P. otaheitana* and/or *P. affinis *(Gerlach, 2016) and all of the montane Tahitian specimens genotyped to‐date (Lee, Burch, Jung et al., 2007; Lee et al., 2014; Figure 3 and Supporting Information Figure S1) cluster with Clade 1 taxa.

Our Moorean Clade 2 phylogenomic results (Figures 3, 4, 5, 6 and Supporting Information Figure S1) broadly corroborated the extensive earlier body of work (Clarke & Murray, 1969; Goodacre, 2001; Johnson et al., 1977; Johnson, Murray, & Clarke1986b; Murray & Clarke, 1980; Murray et al., 1991) inferring the presence of two species complexes on the island: “*P. taeniata*” (*P. exigua* and *P. taeniata*) and “*P. suturalis*” (*P. aurantia*, *P. mooreana*, *P. suturalis* and its subspecies, and *P. tohiveana*). These two complexes reportedly did not hybridize directly but could exchange genes through a seventh Moorean species, *P. mirabilis*, which served as a genetic bridge (Murray & Clarke, 1980). Our genomic data show clear evidence of genetic admixture between the two complexes, but it is difficult to distinguish whether this is a result of introgressive gene flow or of incomplete lineage sorting (Kutschera et al., 2014; Maddison & Knowles, 2006); Moorean *Partula *species radiated recently (Clarke et al., 1996; Johnson et al.1986b) and are estimated to be no more than ~1.7–1.5 million years old (Uto et al., 2007). If the admixture had an introgressive origin, it was predominantly uni‐directional from the “*P. suturalis*” species complex into the “*P. taeniata*” species complex and it exhibited an east–west cline. “*P. taeniata*” individuals (pink) in the east showed lower admixture with “*P. suturalis*” (purple) compared to those in the west (Figure 5). Previous studies of the two Moorean species complexes also noted high genetic similarity in the northwestern portion of the island and lower similarity in the southeast using allozyme (Clarke et al., 1996; Johnson et al.1986b) and mtDNA restriction fragment length polymorphisms (Murray et al., 1991). Nevertheless, wherever the two complexes occurred in sympatry “*P. taeniata*” snails still retained a distinct genomic signature (sites 261, 268, 273, and 276; Figure 5). We therefore concur with Clarke and colleagues (Clarke & Murray, 1969; Goodacre, 2001; Johnson et al., ; Murray & Clarke, 1980; Murray et al., 1991) that two distinct *Partula *species gene pools were maintained on the island of Moorea.

Based on data from 1,607 to 28,194 nuclear genomic loci, 14 of the 18 currently recognized species (Gerlach, 2016) formed five species complexes: “*P. otaheitana*,” “*P. affinis*,” “*P. clara*/*P. hyalina*,” “*P. suturalis*,” and “*P. taeniata*” (Figures 3, 4, 5, 6; summarized in Table 2). These five species complexes do not correspond to the existing morphological taxonomy, and our results revealed extensive genomic poly‐ and paraphyly among the 14 described species analyzed here. This is consistent with results from earlier analyses using allozymes (Clarke et al., 1996; Johnson et al.1986b) and mt sequence data (Lee et al., 2009, 2014; Lee, Burch, Jung et al., 2007) that also showed a lack of correspondence to the taxonomy (Crampton, 1916, 1932; Gerlach, 2016). Each of the Moorean and Tahitian *Partula *species complexes was characterized by a variety of shell phenotypes with no obvious diagnostic features among them (Figure 2). Some common phenotypes included shells that were either sinistral or dextral, solid or striped, dark brown, light brown, white, or some combination, with these forms occurring across most of the clades. Likewise, many of the earlier studies highlighted the phenotypic variability in this group with similar forms found in multiple species (Crampton, 1916, 1932; Johnson et al., 1993b; Murray & Clarke, 1968). This is not an uncommon pattern in young species groups especially those that have undergone recent rapid radiations, such as cichlids (see Salzburger, 2018 and references therein) and Hawaiian Island Tetragnathid spiders (Gillespie, 2004), and as hypothesized for Society Island *Partula *species (Goodacre, 2002; Johnson et al., 1993a; Murray et al., 1993).

**Table 2:** Summary of the five *Partula *species complexes on Moorea and Tahiti including the species and subspecies found within each complex, and the supporting analyses

Clade	Island	Species complex	Constituent taxa	Supporting analyses
Clade 1	Tahiti	“*P. affinis*”	*P. affinis*	Phylogenomic trees (3.2; Figure 3)
			*P. otaheitana*	SVDquartets (3.2; Figure 4)
			*P. o. rubescens*	Structure (3.3; Figure 5)
			*P. o. sinistrorsa*	DAPC (3.3; Figure 6)
			*P. producta*	
		“*P. otaheitana*”	*P. affinis*	Phylogenomic trees (3.2; Figure 3)
			*P. diminuta*	SVDquartets (3.2; Figure 4)
			*P. nodosa composita*	Structure (3.3; Figure 5)
			*P. n. intermedia*	DAPC (3.3; Figure 6)
			*P. otaheitana*	
			*P. o. crassa*	
			*P. o. otaheitana*	
			*P. o. sinistrorsa*	
			*P. producta*	
Clade 2	Tahiti	“*P. clara*/*P. hyalina*”	*P. clara*	Phylogenomic trees (3.2; Figure 3)
			*P. c. incrassa*	SVDquartets (3.2; Figure 4)
			*P. hyalina*	Structure (3.3; Figure 5)
			*P. h. marmorata*	DAPC (3.3; Figure 6)
	Moorea	“*P. taeniata*”	*P. exigua*	SVDquartets (3.2; Figure 4)
			*P. mirabilis*	Structure (3.3; Figure 5)
			*P. taeniata*	DAPC (3.3; Figure 6)
		“*P. suturalis*”	*P. aurantia*	SVDquartets (3.2; Figure 4)
			*P. mirabilis*	Structure (3.3; Figure 5)
			*P. m. propinqua*	DAPC (3.3; Figure 6)
			*P. mooreana*	
			*P. suturalis*	
			*P. s. suturalis*	
			*P. s. vexillum*	
			*P. taeniata*	
			*P. tohiveana*	

Note

Section 3 and corresponding figure for the analysis are in parentheses.

The pervasive discordance among the current Windward Island *Partula *species’ taxonomy and the genomic (Figures 3, 4, 5, 6 and Supporting Information Figure S1), allozyme (Clarke et al., 1996; Johnson et al., 1986a), and mtDNA datasets (Lee et al., 2009, 2014) highlights the need for a taxonomic revision that includes a comprehensive phylogenomic sampling of the 18 Moorean and Tahitian *Partula *species. Finer‐scale analyses have uncovered additional variation across both islands (Figures 5, 6), but how these relate to the taxonomy is still unclear. Further sampling is clearly necessary.

This study has important implications for the current estimates of Windward Island *Partula* species survival and for their conservation prioritization. Our genomic results largely corroborate and further refine the mtDNA survival estimates for Moorea and Tahiti (Lee et al., 2009; Lee, Burch, Jung et al., 2007). They also recovered a close genealogical linkage between the valley survivors on both islands: Moorean *P. taeniata* and Tahitian *P. clara *and *P. hyalina* and their phylogenomic placement and population assignment imply that the latter stem from a founder event by Moorean “*P. taeniata*” (see Figures 3 and 5c, and Supporting Information Figure S1). Previous mt results showed 10/11 mt clades survive in the wild and in captivity (Lee et al., 2009; Lee, Burch, Jung et al., 2007). Here, we uncovered five genomic species complexes, with evidence of all still surviving in the wild, although the details vary in each case. Clade 1 “*P. otaheitana*” and “*P. affinis*” have been extirpated from all but one of the valleys of Tahiti but substantial populations remain (Gerlach, 2016; Lee et al., 2009; Lee, Burch, Jung et al., 2007) in the montane refuges available on that island—Tahiti has ~13 km^2^ of habitat >1,400 m in altitude (Gargominy, 2008). The Tahitian portion of Clade 2 (“*P. clara*/*P. hyalina*” species complex) also persists in the wild as small remnant valley populations (Coote, 2007; Lee et al., 2009) that have successfully survived ~40 years of direct exposure to *E. rosea*, possibly due to elevated clutch sizes in the case of Tahitian populations (Bick et al., 2016). It also survives as prehistorically introduced populations in a number of Austral and Cook Islands (Lee, Burch, Coote et al., 2007; Figure 3 and Supporting Information Figure S1). Moorean Clade 2 also persists in the wild as small remnant *P. taeniata* valley populations (Gerlach, 2016; Lee et al., 2009). Prior to this study, all members of the *“P. suturalis*” species complex were assumed to be long extinct in the wild (Clarke et al., 1984), excluding current experimental reintroductions from captive populations. However, our new genomic data show that a wild specimen identified as *P. taeniata* (MTOI2; Figures 3, 4, 5, 6 and Supporting Information Figure S1), sampled a decade ago, belongs to this complex, an affiliation that had been masked by its divergent mt genotype (Lee et al., 2009). This survivor was encountered on Mt. Tohiea (the highest peak on Moorea) at ~1,150 m, just below the summit (1,207 m), raising the possibility that members of the “*P. suturalis*” species complex still persist there in a small montane refuge.

Three other Windward Island partulid species in the genus *Samoana* survive on Moorea and Tahiti (Lee et al., 2009). Combined with our evidence of five surviving genomic *Partula *species complexes, it appears that the loss of phylogenetically discrete endemic Moorean and Tahitian partulid species has been less than originally feared (Clarke et al., 1984; Coote & Loève, 2003). However, these results for the Windward Islands do not address the losses of *Partula *species on the other Society islands. Notably, 23 endemic species are described from Raiatea but only a single species is still reported as extant (Gerlach, 2016). Species from the other Leeward Islands are extinct, with the exception of *P. rosea *and *P. varia *from Huahine that survive in captivity (Gerlach, 2016).

These new phylogenomic findings should spur on rather than lessen ongoing conservation efforts for Moorean and Tahitian *Partula* taxa*. *The endemic Windward Island genomic clades have suffered catastrophic population declines and losses of phenotypic and population genetic diversity, but they still endure. Continued, proactive conservation and management in the wild and in captivity can still ensure a phylogenetically representative survival of the fabled *Partula* species of Moorea and Tahiti. In the wild, a conservation priority should be placed on confirming the distribution and abundance of the Moorean Clade 2 “*P. suturalis*” species complex remnant populations on Mt. Tohiea (Moorea). The scattered Clade 2 valley populations on Tahiti remain at risk due to their continued exposure to *E. rosea* and to the more recently introduced predatory New Guinea flatworm *Platydemus manokwari *(Gerlach, 2016). Members of Clade 1 surviving in Tahitian montane refuges need continued monitoring and habitat protection to ensure their survival. In captivity, the *Partula *Global Species Management Programme breeding program currently maintains representatives of all five species complexes and a substantial amount of the genetic variation that was present prior to the introduction of *E. rosea *(Figure 3). Our findings highlight the need for continuation of the captive program and also illustrate the fundamental value of research museum biodiversity holdings. J.B. Burch could not have envisaged the impending collapse of these endemic partulid populations in 1970, but the specimens he collected remain a critical research resource in understanding the scale of the loss and in developing an informed conservation strategy.

## CONFLICT OF INTEREST

The authors declare no conflict of interests or competing financial interests.

## Data Availability

The raw data for each of the 117 *Partula *individuals and the three *Samoana *from the Illumina HiSeq were deposited in NCBI's Sequence Read Archive (SRA; Accession # PRJNA326969). Parameter files used to generate the 85, 90, and 95% threshold datasets for 75, 50, and 25% of taxa from ipyrad were deposited in the Dryad Digital Repository (https://doi.org/10.5061/dryad.2j1d35d) along with all data matrices used to construct the maximum likelihood and Bayesian trees, SVDquartets tree, Structure, DAPC, and TreeMix analyses. We also deposited the single SNP.vcf file used to generate the matrices for Structure, DAPC, TreeMix, and SVDquartets tree. All relevant data also are available from the authors.
