# Supplementary material for: Deconstructing an infamous extinction crisis: Survival of Partula species on Moorea and Tahiti
Source: Evol Appl. 2019 Feb 27;12(5):1017–33. doi: 10.1111/eva.12778 (PMC6503832; doi:10.1111/eva.12778)
Supplement: Supplementary file 1 [file EVA-12-1017-s001.pdf]

Supporting Information for

# Deconstructing an infamous extinction crisis: survival of *Partula* species on Moorea and Tahiti

Amanda E. Haponski, Taehwan Lee, Diarmaid Ó Foighil

*Evolutionary Applications*

**Table S1** Summary of *Partula* species and individuals used for ddRADseq data collection. Information for each individual includes island, valley, and site of origin, University of Michigan's Museum of Zoology (UMMZ) individual identification number, UMMZ shell voucher catalog number, and species complex designations for Moorean and Tahitian specimens. See Figures 3 and S1 for placement on the tree and Figures 5 and 6 for species complex assignments. Individuals from the "*P. otaheitana*" and "*P. clara/P. hyalina*" species complexes are labelled with their corresponding clade/population identifier (i.e., A, B, C). Black= 1970 lyophilized individuals collected by J.B. Burch, blue= remnant valley population samples, and red= captive population specimens.

| Species | Island | Valley | Site | Individual | Voucher # | Species Complex |
|---|---|---|---|---|---|---|
| *P. aurantia* Crampton 1932 | Moorea | Faamaariri (300m) | 270 | M1 | 300516 | "*P. suturalis*" |
| *P. exigua* Crampton 1917 | Moorea | Paparoa (300m) | 268 | 132b1 | 300508 | "*P. taeniata*" |
| | | Faamaariri (150m) | 269 | 51a1 | 300512 | "*P. taeniata*" |
| *P. mirabilis* Crampton 1924 | Moorea | Matapoopoo (150-250m) | 273 | M9 | 300531 | "*P. taeniata*" |
| | | Matapoopoo (150-250m) | 273 | 130d1 | 300533 | "*P. taeniata*" |
| | | Mouaroa (200-300m) | 275 | PM67a1 | 300492 | "*P. suturalis*" |
| | | Fareaito | 11 | PMIZ1 | 301016 | "*P. suturalis*" |
| *P. m. propinqua* Crampton 1932 | Moorea | Matapoopoo (150-250m) | 273 | 135a3 | | "*P. suturalis*" |
| *P. mooreana* Hartman 1830 | Moorea | Atimaha Ridge | 13 | MOOM2 | 301018 | "*P. suturalis*" |
| *P. suturalis* Pfeiffer 1855 | Moorea | Faamaariri (300m) | 270 | 116a1 | 300519 | "*P. suturalis*" |
| | | Hotutea (620-700m) | 279 | 118b1 | 300557 | "*P. suturalis*" |
| | | Hotutea (620-700m) | 279 | 118d1 | 300557 | "*P. suturalis*" |
| *P. s. suturalis* (Pfeiffer 1855) | Moorea | Hotutea (400-480m) | 276 | 121d1 | 300556 | "*P. suturalis*" |
| | | Hotutea (620-700m) | 279 | 123b1 | 300558 | "*P. suturalis*" |
| | | Maatea | 12 | STRM1 | 301019 | "*P. suturalis*" |
| *P. s. vexillum* (Pease 1866) | Moorea | Mt. Tautua (350m) | 261 | M7 | 300487 | "*P. suturalis*" |
| | | Paparoa (350m) | 268 | 126c1 | 300505 | "*P. suturalis*" |
| | | Matapoopoo (400m) | 274 | 50b1 | 300537 | "*P. suturalis*" |
| | | Mouaroa (200-300m) | 275 | 127a1 | 300539 | "*P. suturalis*" |
| | | Fareaito | 11 | VEXM1 | 301021 | "*P. suturalis*" |
| | | Vaianai | 14 | VEXM2 | 301022 | "*P. suturalis*" |
| *P. taeniata* (Mörch 1850) | Moorea | Faatoai | 261 | M10 | 300489 | "*P. taeniata*" |
| | | Paparoa (100m) | 267 | 131b1 | 300496 | "*P. taeniata*" |
| | | Mt. Rotui (150-250m) | 273 | 130a1 | 300527 | "*P. taeniata*" |
| | | Mt. Rotui (150-250m) | 273 | 130c1 | 300532 | "*P. taeniata*" |
| | | Hotutea (400-480m) | 276 | PS136a1 | 300544 | "*P. taeniata*" |

| | | | | | | |
|---|---|---|---|---|---|---|
| | | Faatoai | 15 | NUCM1 | 301023 | "*P. taeniata*" |
| | | Haumi | 2 | PHAU | | "*P. taeniata*" |
| | | Opunohu Bay | 5 | TAEH1 | | "*P. taeniata*" |
| | | Mt. Tohiea (1150m) | 6 | MTOI2 | | "*P. suturalis*" |
| *P. tohiveana* Crampton 1924 | Moorea | Hotutea (480m) | 278 | M5 | 300547 | "*P. suturalis*" |
| | | Fareaito | 11 | TOHM1 | 301026 | "*P. suturalis*" |
| *P. affinis* Pease 1867 | Tahiti | Afeu R. | 107 | 73a1 | 300363 | "*P. otaheitana*" A |
| | | Tirahi | 139 | Y30 | 300409 | "*P. affinis*" |
| | | Vaitepiha | 142 | 75a1 | 300411 | "*P. affinis*" |
| | | Vaitehoro R. | 152 | 76a1 | 300417 | "*P. affinis*" |
| | | Ahaavini | 161 | 79a1 | 300441 | "*P. affinis*" |
| | | Tiitauiri R. | 174 | 80a1 | 300452 | "*P. affinis*" |
| | | Tereia R. | 178 | 28a1 | 300464 | "*P. otaheitana*" A |
| | | Fareteuira R. | 198 | 83a1 | 300476 | "*P. affinis*" |
| | | Tuaura R. | 211 | 84a1 | 300482 | "*P. affinis*" |
| | | Te Pari District | 3 | PAZL1 | 300560 | "*P. affinis*" |
| *P. clara* Pease 1864 | Tahiti | Taharuu R. | 125 | 26a1 | 300393 | "*P. clara/P. hyalina*" C |
| | | Tahiria R. | 169 | 46a1 | 300453 | "*P. clara/P. hyalina*" C |
| | | Tahiria R. | 169 | 46b1 | 300454 | "*P. clara/P. hyalina*" C |
| | | Paihau R. | 224 | 48a2 | | "*P. clara/P. hyalina*" B |
| | | Maruapo (120m) | 14 | PCTM | | "*P. clara/P. hyalina*" B |
| *P. c. incrassa* Crampton 1916 | | Tiapa | 37 | PCTI | | "*P. clara/P. hyalina*" B |
| *P. diminuta* Adams 1851 | Tahiti | Pirae | 200 | 54a4 | 300428, 429 | "*P. otaheitana*" B |
| | | Pirae | 200 | 54d2 | 300479 | "*P. otaheitana*" B |
| *P. hyalina* Broderip 1832 | Tahiti | Tetiairiroa | 192 | 60a1 | 300471 | "*P. clara/P. hyalina*" A |
| | | Haapupuni | 4 | PHTH2 | | "*P. clara/P. hyalina*" A |
| | | Matatia (120m) | 15 | PHTM | | "*P. clara/P. hyalina*" A |
| | Austral Is. | Falises de Matonaa | | PHRM1 | 300610 | "*P. clara/P. hyalina*" A |
| | | Rimatara | | PHRM314 | | "*P. clara/P. hyalina*" A |
| *P. h. marmorata* (Crampton 1916) | | Toheimahu R. | 135 | 41a1 | 300396, 398 | "*P. clara/P. hyalina*" A |
| | | Vaitepiha | 142 | 43a1 | 300412 | "*P. clara/P. hyalina*" A |
| *P. nodosa composita* Crampton 1917 | Tahiti | Punaruu R. | 91 | Y28 | | "*P. otaheitana*" B |
| *P. n. intermedia* Crampton 1917 | Tahiti | Papehue R. | 44 | PNZL1 | 300565 | "*P. otaheitana*" A |
| *P. otaheitana* (Brugière 1789) | Tahiti | Punaruu R. | 94 | 49a1 | 300359 | "*P. otaheitana*" A |
| | | Moaroa R. | 120 | 39a11 | 300386 | "*P. affinis*" |
| | | Tirahi R. | 138 | 72a1 | 300408 | "*P. affinis*" |
| | | Mt. Marau | 5 | POZL12 | 300567 | "*P. otaheitana*" B |
| | | Mt. Marau | 5 | POZL4 | 300570 | "*P. otaheitana*" B |
| | | Mt. Aorai Fare Mato (1400m) | 1 | POMA | | "*P. otaheitana*" B |
| | | Mt. Aorai Fare Mato (1400m) | 1 | POMA1 | | "*P. otaheitana*" B |

| | | | | | | |
|---|---|---|---|---|---|---|
| *P. o. crassa* Pease 1884 | Tahiti | Punaruu R. | 90 | Y2-2 | 300357 | "*P. otaheitana*" B |
| | | Orofero | 99 | Y35 | 300361 | "*P. otaheitana*" A |
| *P. o. otaheitana* (Brugière 1789) | Tahiti | Fautaua R. | 144 | 93a1 | 300414 | "*P. otaheitana*" B |
| | | Fautaua R. | 144 | Y37 | 300414 | "*P. otaheitana*" B |
| | | Tipaerui Valley | 217 | Y36 | 300431 | "*P. otaheitana*" B |
| | | Fautaua R. | 221 | 101a1 | 300485 | "*P. otaheitana*" B |
| *P. o. rubescens* Reeve 1850 | Tahiti | Ahaavini | 159 | 97b1 | 300419, 421-4 | "*P. affinis*" |
| | | Onoheha | 186 | 98a1 | 300425 | "*P. affinis*" |
| | | Tetiairiroa | 192 | 99b1 | 300468 | "*P. affinis*" |
| | | Tetiairiroa | 192 | 99c1 | 300468 | "*P. affinis*" |
| | | Taravao Plateau | 243 | 100a1 | 300434 | "*P. affinis*" |
| | | Mt. Atara (1050m) | 10 | PRIA | | "*P. affinis*" |
| *P. o. sinistrorsa* Pease 1884 | Tahiti | Afeu R. | 107 | 109a1 | 300365, 366 | "*P. otaheitana*" A |
| | | Temarua | 110 | Y39 | 300372 | "*P. otaheitana*" A |
| | | Temarua | 113 | 104b1 | 300376 | "*P. otaheitana*" A |
| | | Temarua | 113 | 104c1 | 300377 | "*P. otaheitana*" A |
| | | Faurahi R. | 117 | 110b1 | 300378 | "*P. otaheitana*" A |
| | | Moaroa R. | 120 | 111c1 | 300385, 387 | "*P. otaheitana*" A |
| | | Papeiti R. | 123 | Y40 | 300389, 390 | "*P. otaheitana*" A |
| | | Taharuu R. | 125 | Y18 | 300391, 393 | "*P. otaheitana*" A |
| | | Tahiria R. | 170 | Y42 | 300455 | "*P. otaheitana*" A |
| | | Tereia R. | 180 | 106a1 | 300465 | "*P. otaheitana*" A |
| *P. producta* Pease 1865 | Tahiti | Afeu R. | 106 | 108b1 | 300362 | "*P. affinis*" |
| | | Faurahi R. | 117 | Y10 | 300379 | "*P. otaheitana*" A |
| | | Faurahi R. | 117 | Y44 | 300379 | "*P. otaheitana*" A |
| | | Toheimahu R. | 135 | 71a1 | 300397 | "*P. otaheitana*" A |
| *P. sp.* | Tahiti | Mt. Marau | 16 | PTMT4 | | "*P. otaheitana*" B |
| *P. rosea* Broderip 1832 | Huahine | Mauhuti | | ROSH1 | 304374 | |
| *P. varia* Broderip 1832 | Huahine | | | VARH1 | 304375 | |
| *P. clarkei* (Pease 1865) | Raiatea | | | PTUG1 | 300630 | |
| | | | | PTUG2 | 300630 | |
| | | | | PTUG4 | 300630 | |
| *P. faba* (Gmelin 1791) | Raiatea | Vaiaau | | 33a2 | 300633 | |
| | | Tupua Bay | | 35a1 | 300632 | |
| | | Hamoa | | FABR3 | 300631 | |
| *P. garrettii* Crampton and Cooke 1953 | Raiatea | Tevaitoa | | TRIR1 | 300629 | |
| *P. hebe* (Pfeiffer 1846) | Raiatea | Vaiaau | | 31a2 | 300622 | |
| *P. h. bella* Pease 1881 | Raiatea | Hotopuu | | HEBR1 | 300623 | |
| *P. meyeri* Burch 2007 | Raiatea | Mt. Tefatua (950m) | | SRMT | 300621 | |
| *P. navigatoria* Pfeiffer 1854 | Raiatea | Hamoa | | DENR1 | 300624 | |
| | | | | DENR2 | 300624 | |

| | | | | | |
|---|---|---|---|---|---|
| *P. assimilis* Pease 1868 | Rarotonga, Cook Is. | Takuraine | | PHRT1 | 304370 |
| *P. lutea* Lesson 1831 | Bora Bora | Mt. Pahia | | 65a1 | 304371 |
| | | | | | |
| Outgroups | | | | | |
| *P. auraniana* Hartman 1888 | Toga, Vanuatu | | | PVT27-1 | |
| *P. cramptoni* | Bellona, Solomon Is. | | | B1 | 305032 |
| | Bellona, Solomon Is. | | | B2 | 305032 |
| *P. lirata* Mousson 1865 | Thikombia-i-lau, Fiji | | | PLIR3 | 304368 |
| | Thikombia-i-lau, Fiji | | | PLIR4 | 304368 |
| *P. pacifica* Pfeiffer 1860 | Erromango, Vanuatu | | | PTUN2 | FMNH175385 |
| | | | | PTUN4 | FMNH175385 |
| | Tanna, Vanuatu | | | PTUR7 | FLMNH58233 |
| *Samoana attenuata* (Pease 1864) | Tahiti | Haapupuni (115m) | | SATH | |
| *S. burchi* Kondo 1973 | Tahiti | Taravao Plateau | 243 | SS23a1 | 300433 |
| *S. diaphana* (Crampton and Cooke 1953) | Moorea | Hotutea | 279 | SD25a1 | 300548 |

**Table S2** Summary of ddRADseq data for each partulid individual including the number of raw reads, number of loci passing the paralog filter with a sequencing depth >6, and mean depth of loci with sequencing depth >6 for 85, 90, and 95% similarity thresholds. Black= 1970 lyophilized individuals collected by J.B. Burch, blue= remnant valley population samples, and red= captive population specimens.

| | | | 85% similarity | | 90% similarity | | 95% similarity | |
|---|---|---|---|---|---|---|---|---|
| Species | Individual | Raw reads | Loci | Depth | Loci | Depth | Loci | Depth |
| *P. aurantia* | M1 | 1,603,738 | 26,353 | 42.9 | 28,131 | 42.3 | 29,586 | 40.5 |
| *P. exigua* | 132b1 | 1,029,088 | 26,068 | 26.1 | 27,609 | 25.7 | 28,489 | 24.7 |
| | 51a1 | 1,260,058 | 22,944 | 41.0 | 24,105 | 40.5 | 24,748 | 39.2 |
| *P. mirabilis* | M9 | 876,599 | 20,906 | 21.5 | 22,232 | 21.2 | 23,072 | 20.6 |
| | 130d1 | 1,397,447 | 25,165 | 35.6 | 26,857 | 35.2 | 28,206 | 33.8 |
| | PM67a1 | 2,261,899 | 28,957 | 62.4 | 30,921 | 61.2 | 32,790 | 58.0 |
| | PMIZ1 | 1,717,056 | 24,736 | 51.9 | 25,975 | 51.3 | 26,713 | 49.8 |
| *P. m. propinqua* | 135a3 | 1,230,250 | 24,900 | 37.0 | 26,457 | 36.4 | 27,664 | 35.0 |
| *P. mooreana* | MOOM2 | 3,181,640 | 31,431 | 80.8 | 34,389 | 79.1 | 37,156 | 76.3 |
| *P. suturalis* | 116a1 | 2,192,660 | 30,457 | 53.6 | 33,770 | 52.4 | 37,009 | 50.1 |
| | 118b1 | 2,521,705 | 30,471 | 60.8 | 33,719 | 59.3 | 37,130 | 56.5 |
| | 118d1 | 2,015,712 | 27,714 | 54.2 | 30,498 | 53.4 | 33,389 | 50.9 |
| *P. s. suturalis* | 121d1 | 2,406,404 | 30,590 | 58.3 | 33,569 | 57.2 | 36,818 | 54.7 |
| | 123b1 | 2,529,319 | 33,772 | 55.3 | 37,516 | 54.0 | 41,361 | 51.4 |
| | STRM1 | 2,072,644 | 26,210 | 63.1 | 28,498 | 61.8 | 30,773 | 59.4 |
| *P. s. vexillum* | M7 | 1,421,901 | 24,460 | 41.7 | 26,994 | 40.6 | 29,332 | 39.2 |
| | 126c1 | 2,777,238 | 31,515 | 70.8 | 33,718 | 69.2 | 36,122 | 65.6 |
| | 50b1 | 1,464,583 | 23,832 | 45.9 | 25,852 | 45.3 | 27,722 | 43.9 |
| | 127a1 | 2,208,049 | 29,393 | 56.3 | 32,419 | 55.0 | 35,543 | 52.5 |
| | VEXM1 | 2,009,859 | 26,435 | 60.3 | 28,689 | 59.2 | 30,912 | 57.1 |
| | VEXM2 | 2,195,398 | 26,889 | 65.2 | 29,250 | 64.0 | 31,601 | 61.7 |
| *P. taeniata* | M10 | 1,201,048 | 28,012 | 27.8 | 29,546 | 27.4 | 30,500 | 26.4 |
| | 131b1 | 965,658 | 27,158 | 24.3 | 28,613 | 23.9 | 29,382 | 23.0 |

| | | | | | | | | |
|---|---|---|---|---|---|---|---|---|
| | 130a1 | 1,686,229 | 33,518 | 37.6 | 35,614 | 36.9 | 37,206 | 35.2 |
| | 130c1 | 1,503,655 | 28,720 | 37.8 | 30,150 | 37.4 | 31,281 | 35.6 |
| | PS136a1 | 1,047,840 | 26,202 | 25.9 | 27,597 | 25.6 | 28,351 | 24.7 |
| | NUCM1 | 2,903,135 | 31,606 | 71.4 | 34,722 | 70.1 | 37,609 | 67.5 |
| | PHAU | 1,647,003 | 35,429 | 35.7 | 37,468 | 35.0 | 38,807 | 33.5 |
| | TAEH1 | 1,433,170 | 30,508 | 34.4 | 32,026 | 33.9 | 33,015 | 32.6 |
| | MTOI2 | 1,071,454 | 19,949 | 27.1 | 21,115 | 26.9 | 21,797 | 26.7 |
| *P. tohiveana* | M5 | 1,150,028 | 22,959 | 32.2 | 24,416 | 31.9 | 25,378 | 30.8 |
| | TOHM1 | 2,061,075 | 26,663 | 61.8 | 28,961 | 60.8 | 31,228 | 58.5 |
| *P. affinis* | 73a1 | 2,113,990 | 27,649 | 57.3 | 30,460 | 56.0 | 33,182 | 53.6 |
| | Y30 | 2,416,205 | 30,613 | 60.4 | 33,476 | 59.1 | 36,411 | 56.7 |
| | 75a1 | 2,070,268 | 29,492 | 52.8 | 32,203 | 52.0 | 34,688 | 50.3 |
| | 76a1 | 1,674,304 | 25,873 | 48.7 | 28,325 | 47.5 | 30,791 | 45.4 |
| | 79a1 | 1,940,454 | 29,069 | 50.5 | 31,838 | 49.5 | 34,644 | 47.5 |
| | 80a1 | 2,271,322 | 29,632 | 56.9 | 32,653 | 55.4 | 35,521 | 53.3 |
| | 28a1 | 2,267,876 | 28,612 | 60.1 | 31,449 | 58.7 | 34,339 | 56.2 |
| | 83a1 | 2,027,464 | 28,746 | 52.1 | 31,650 | 51.0 | 34,573 | 48.9 |
| | 84a1 | 1,282,175 | 24,337 | 38.8 | 26,498 | 38.1 | 28,728 | 36.4 |
| | PAZL1 | 2,470,161 | 29,780 | 66.3 | 32,478 | 64.9 | 35,154 | 62.8 |
| *P. clara* | 26a1 | 1,485,937 | 25,572 | 44.0 | 26,530 | 43.8 | 27,029 | 42.6 |
| | 46a1 | 1,092,449 | 25,884 | 28.2 | 27,099 | 27.8 | 27,691 | 26.9 |
| | 46b1 | 1,382,197 | 24,765 | 41.5 | 26,220 | 40.8 | 27,009 | 39.9 |
| | 48a2 | 1,399,520 | 25,698 | 41.1 | 26,703 | 40.7 | 27,120 | 39.3 |
| | PCTM | 1,469,706 | 31,231 | 36.1 | 32,612 | 35.8 | 33,179 | 34.6 |
| *P. c. incrassa* | PCTI | 1,417,747 | 35,101 | 27.9 | 38,558 | 27.8 | 41,240 | 27.0 |
| *P. diminuta* | 54a4 | 1,546,869 | 26,304 | 42.7 | 29,013 | 41.8 | 31,565 | 40.8 |
| | 54d2 | 1,647,106 | 28,150 | 41.9 | 31,049 | 41.4 | 33,619 | 40.7 |
| *P. hyalina* | 60a1 | 1,204,836 | 26,725 | 31.9 | 27,961 | 31.6 | 28,336 | 30.7 |
| | PHTH2 | 4,850,061 | 35,995 | 113.8 | 38,257 | 112.0 | 40,090 | 108.1 |
| | PHTM | 2,035,731 | 35,567 | 45.7 | 37,455 | 44.7 | 38,050 | 43.0 |
| | PHRM1 | 2,259,592 | 39,083 | 46.1 | 41,249 | 45.1 | 42,354 | 43.1 |

| | | | | | | | | |
|---|---|---|---|---|---|---|---|---|
| | PHRM314 | 282,185 | 13,802 | 12.0 | 14,309 | 11.7 | 14,265 | 11.3 |
| *P. h. marmorata* | 41a1 | 1,148,513 | 26,272 | 29.7 | 27,578 | 29.5 | 28,076 | 28.7 |
| | 43a1 | 1,111,388 | 26,473 | 27.5 | 27,674 | 27.1 | 28,078 | 26.2 |
| *P. nodosa composita* | Y28 | 1,751,729 | 27,570 | 44.9 | 30,491 | 44.2 | 33,172 | 43.3 |
| *P. n. intermedia* | PNZL1 | 1,619,568 | 32,603 | 36.1 | 34,944 | 35.6 | 36,996 | 34.9 |
| *P. otaheitana* | 49a1 | 2,218,454 | 30,518 | 52.8 | 33,721 | 52.0 | 36,830 | 50.8 |
| | 39a11 | 2,415,294 | 32,519 | 52.1 | 36,105 | 51.3 | 39,331 | 50.3 |
| | 72a1 | 2,045,871 | 28,955 | 51.9 | 32,044 | 51.3 | 34,740 | 50.3 |
| | POZL12 | 976,584 | 26,930 | 24.8 | 29,078 | 24.5 | 30,947 | 24.0 |
| | POZL4 | 2,177,854 | 32,890 | 49.9 | 36,106 | 49.6 | 38,774 | 48.6 |
| | POMA | 1,855,373 | 27,335 | 53.0 | 29,823 | 52.1 | 32,223 | 51.2 |
| | POMA1 | 2,185,828 | 28,737 | 57.9 | 31,584 | 57.2 | 33,952 | 56.3 |
| *P. o. crassa* | Y2-2 | 1,879,754 | 28,820 | 44.5 | 32,032 | 43.8 | 34,971 | 42.9 |
| | Y35 | 1,536,598 | 26,213 | 41.5 | 28,999 | 40.8 | 31,522 | 40.0 |
| *P. o. otaheitana* | 93a1 | 1,689,707 | 27,275 | 44.4 | 30,222 | 43.6 | 32,889 | 42.6 |
| | Y37 | 2,269,001 | 30,328 | 51.5 | 33,765 | 50.7 | 36,956 | 49.8 |
| | Y36 | 1,968,658 | 29,682 | 47.4 | 32,931 | 46.7 | 35,977 | 45.6 |
| | 101a1 | 2,314,825 | 30,088 | 61.0 | 32,135 | 59.6 | 34,115 | 57.0 |
| *P. o. rubescens* | 97b1 | 1,230,760 | 26,194 | 32.2 | 28,983 | 31.6 | 31,432 | 30.9 |
| | 98a1 | 1,665,443 | 26,179 | 46.0 | 28,761 | 45.3 | 30,967 | 44.8 |
| | 99b1 | 2,056,240 | 29,291 | 52.2 | 32,504 | 51.2 | 35,447 | 49.9 |
| | 99c1 | 1,727,192 | 26,377 | 47.9 | 28,972 | 47.2 | 31,380 | 46.4 |
| | 100a1 | 2,338,609 | 31,886 | 58.1 | 34,097 | 56.7 | 36,168 | 54.2 |
| | PRIA | 2,281,567 | 28,926 | 59.8 | 31,572 | 59.2 | 33,931 | 58.3 |
| *P. o. sinistrorsa* | 109a1 | 1,877,837 | 27,340 | 51.7 | 28,939 | 50.9 | 30,530 | 48.7 |
| | Y39 | 2,199,338 | 28,798 | 58.6 | 30,603 | 57.7 | 32,423 | 55.1 |
| | 104b1 | 1,678,464 | 26,658 | 49.2 | 28,269 | 48.5 | 29,828 | 46.5 |
| | 104c1 | 1,754,959 | 25,829 | 51.7 | 27,259 | 50.8 | 28,550 | 49.1 |
| | 110b1 | 1,812,334 | 27,744 | 51.9 | 29,541 | 51.1 | 31,165 | 49.2 |
| | 111c1 | 1,160,317 | 25,323 | 33.7 | 26,920 | 33.2 | 27,991 | 32.1 |
| | Y40 | 1,815,907 | 26,089 | 51.0 | 27,663 | 50.2 | 29,052 | 48.4 |

| | | | | | | | | |
|---|---|---|---|---|---|---|---|---|
| | Y18 | 2,765,922 | 32,245 | 64.9 | 34,515 | 64.1 | 36,794 | 61.2 |
| | Y42 | 1,982,348 | 27,425 | 52.6 | 29,191 | 51.8 | 30,715 | 49.8 |
| | 106a1 | 1,814,093 | 25,488 | 54.0 | 26,962 | 53.4 | 28,008 | 51.7 |
| *P. producta* | 108b1 | 2,296,056 | 29,406 | 61.4 | 31,291 | 60.4 | 33,087 | 57.8 |
| | Y10 | 5,705,274 | 67,382 | 56.7 | 75,243 | 56.4 | 84,320 | 55.1 |
| | Y44 | 1,626,373 | 26,801 | 45.6 | 28,542 | 44.9 | 29,832 | 43.4 |
| | 71a1 | 2,135,448 | 30,730 | 50.1 | 33,898 | 49.4 | 36,846 | 48.4 |
| *P. sp.* | PTMT4 | 1,388,918 | 25,724 | 40.3 | 28,215 | 39.8 | 30,364 | 39.1 |
| *P. rosea* | ROSH1 | 1,962,303 | 24,827 | 63.6 | 26,054 | 62.8 | 26,799 | 61.1 |
| *P. varia* | VARH1 | 1,485,876 | 24,346 | 46.2 | 25,649 | 45.8 | 26,682 | 44.0 |
| *P. clarkei* | PTUG1 | 502,806 | 19,321 | 16.2 | 20,206 | 16.0 | 20,734 | 15.7 |
| | PTUG2 | 998,489 | 36,246 | 17.5 | 39,360 | 17.2 | 41,610 | 16.9 |
| | PTUG4 | 2,651,654 | 50,726 | 35.5 | 55,875 | 35.0 | 60,359 | 33.8 |
| *P. faba* | 33a2 | 1,481,920 | 23,871 | 43.8 | 25,256 | 43.1 | 26,279 | 41.8 |
| | 35a1 | 1,138,968 | 23,186 | 35.8 | 24,532 | 35.2 | 25,651 | 33.8 |
| | FABR3 | 1,201,712 | 24,299 | 35.6 | 25,509 | 35.3 | 26,372 | 34.1 |
| *P. garrettii* | TRIR1 | 1,936,840 | 29,198 | 53.5 | 31,116 | 52.3 | 32,338 | 50.4 |
| *P. hebe* | 31a2 | 1,483,777 | 24,278 | 44.3 | 25,715 | 43.6 | 26,833 | 41.9 |
| *P. h. bella* | HEBR1 | 1,632,905 | 24,614 | 52.9 | 25,937 | 52.2 | 26,828 | 50.5 |
| *P. meyeri* | SRMT | 2,007,575 | 25,812 | 59.4 | 27,115 | 58.6 | 28,149 | 56.4 |
| *P. navigatoria* | DENR1 | 2,392,057 | 26,431 | 70.3 | 27,849 | 69.0 | 28,947 | 66.6 |
| | DENR2 | 1,426,305 | 24,036 | 44.9 | 25,324 | 44.4 | 26,212 | 42.9 |
| *P. assimilis* | PHRT1 | 2,933,054 | 29,828 | 78.0 | 31,341 | 77.3 | 32,119 | 75.3 |
| *P. lutea* | 65a1 | 2,235,354 | 28,396 | 62.0 | 30,227 | 60.9 | 31,965 | 57.7 |
| Outgroups | | | | | | | | |
| *P. auraniana* | PVT27-1 | 1,390,541 | 24,354 | 44.7 | 25,617 | 44.2 | 26,169 | 43.0 |
| *P. cramptoni* | B1 | 2,744,600 | 26,546 | 79.8 | 28,009 | 79.0 | 28,758 | 77.1 |
| | B2 | 2,925,185 | 28,931 | 85.3 | 30,789 | 84.2 | 32,208 | 81.1 |
| *P. lirata* | PLIR3 | 804,236 | 22,555 | 25.9 | 23,798 | 25.7 | 24,455 | 25.3 |
| | PLIR4 | 834,187 | 30,491 | 14.2 | 33,380 | 14.1 | 35,258 | 13.9 |

| | | | | | | | | |
|---|---|---|---|---|---|---|---|---|
| *P. pacifica* | PTUN2 | 274,845 | 7,753 | 10.6 | 8,377 | 10.5 | 8,492 | 10.3 |
| | PTUN4 | 618,459 | 22,390 | 13.4 | 24,713 | 13.3 | 26,487 | 13.2 |
| | PTUR7 | 165,507 | 5,385 | 9.0 | 5,544 | 9.1 | 5,514 | 8.8 |
| *Samoana attenuata* | SATH | 2,111,133 | 33,438 | 52.7 | 35,168 | 51.9 | 36,130 | 50.1 |
| *S. burchi* | SS23a1 | 2,179,667 | 35,905 | 36.1 | 38,013 | 35.4 | 39,109 | 34.2 |
| *S. diaphana* | SD25a1 | 2,342,640 | 37,554 | 47.9 | 39,941 | 46.9 | 41,107 | 44.9 |

**Table S3** Summary of the final number of ddRADseq loci for each partulid individual for the 85, 90, and 95% similarity thresholds and 75, 50, and 25% taxon coverage datasets.

| Species | Individual | 85% Similarity | | | 90% Similarity | | | 95% Similarity | | |
|---|---|---|---|---|---|---|---|---|---|---|
| | | 75% | 50% | 25% | 75% | 50% | 25% | 75% | 50% | 25% |
| *P. aurantia* | M1 | 1,496 | 6,888 | 8,745 | 2,035 | 9,175 | 11,716 | 2,320 | 9,433 | 13,661 |
| *P. exigua* | 132b1 | 1,343 | 6,764 | 9,113 | 1,817 | 8,986 | 12,064 | 2,097 | 9,187 | 14,085 |
| | 51a1 | 1,339 | 6,449 | 7,916 | 1,803 | 8,513 | 10,479 | 2,068 | 8,658 | 12,143 |
| *P. mirabilis* | M9 | 1,212 | 6,238 | 7,368 | 1,619 | 8,259 | 9,847 | 1,881 | 8,410 | 11,366 |
| | 130d1 | 1,387 | 6,603 | 8,299 | 1,883 | 8,798 | 11,091 | 2,164 | 9,013 | 12,867 |
| | PM67a1 | 1,482 | 6,900 | 9,184 | 2,002 | 9,168 | 12,207 | 2,278 | 9,417 | 14,129 |
| | PMIZ1 | 1,489 | 6,810 | 8,581 | 1,984 | 9,002 | 11,389 | 2,251 | 9,202 | 13,126 |
| *P. m. propinqua* | 135a3 | 1,416 | 6,678 | 8,301 | 1,921 | 8,880 | 11,136 | 2,209 | 9,070 | 12,843 |
| *P. mooreana* | MOOM2 | 1,566 | 4,031 | 9,648 | 2,097 | 5,359 | 12,123 | 2,363 | 6,294 | 15,234 |
| *P. suturalis* | 116a1 | 1,586 | 3,955 | 9,039 | 2,143 | 5,308 | 11,757 | 2,424 | 6,263 | 14,877 |
| | 118b1 | 1,591 | 4,026 | 9,185 | 2,150 | 5,373 | 11,752 | 2,420 | 6,328 | 14,821 |
| | 118d1 | 1,588 | 4,000 | 8,402 | 2,143 | 5,331 | 11,047 | 2,422 | 6,272 | 14,132 |
| *P. s. suturalis* | 121d1 | 1,587 | 3,971 | 9,219 | 2,144 | 5,320 | 11,792 | 2,418 | 6,232 | 14,850 |
| | 123b1 | 1,592 | 4,012 | 9,572 | 2,147 | 5,398 | 12,179 | 2,424 | 6,316 | 15,305 |
| | STRM1 | 1,576 | 3,943 | 8,671 | 2,121 | 5,266 | 11,127 | 2,394 | 6,204 | 14,101 |
| *P. s. vexillum* | M7 | 1,548 | 3,723 | 7,934 | 2,105 | 4,990 | 10,490 | 2,411 | 5,918 | 13,539 |
| | 126c1 | 1,549 | 7,267 | 9,923 | 2,095 | 9,699 | 13,215 | 2,374 | 9,890 | 15,230 |
| | 50b1 | 1,556 | 3,758 | 7,835 | 2,098 | 4,989 | 10,297 | 2,383 | 5,881 | 13,190 |
| | 127a1 | 1,586 | 3,955 | 8,661 | 2,149 | 5,326 | 11,297 | 2,418 | 6,254 | 14,330 |
| | VEXM1 | 1,565 | 3,873 | 8,937 | 2,111 | 5,148 | 11,536 | 2,398 | 6,083 | 14,570 |
| | VEXM2 | 1,570 | 3,873 | 8,972 | 2,114 | 5,163 | 11,525 | 2,380 | 6,100 | 14,531 |
| *P. taeniata* | M10 | 1,435 | 6,974 | 9,643 | 1,943 | 9,210 | 12,713 | 2,217 | 9,450 | 14,854 |
| | 131b1 | 1,478 | 7,145 | 9,476 | 1,983 | 9,446 | 12,518 | 2,256 | 9,608 | 14,595 |

| | | | | | | | | | | |
|---|---|---|---|---|---|---|---|---|---|---|
| | 130a1 | 1,559 | 7,559 | 11,290 | 2,098 | 9,955 | 14,731 | 2,361 | 10,226 | 17,198 |
| | 130c1 | 1,425 | 6,932 | 9,511 | 1,921 | 9,151 | 12,515 | 2,204 | 9,351 | 14,627 |
| | PS136a1 | 1,354 | 6,720 | 8,943 | 1,821 | 8,849 | 11,750 | 2,074 | 9,052 | 13,788 |
| | NUCM1 | 1,572 | 3,981 | 9,578 | 2,126 | 5,330 | 12,095 | 2,385 | 6,246 | 15,131 |
| | PHAU | 1,570 | 7,700 | 12,170 | 2,107 | 10,122 | 15,665 | 2,376 | 10,361 | 18,268 |
| | TAEH1 | 1,526 | 7,377 | 10,696 | 2,046 | 9,682 | 13,926 | 2,329 | 9,970 | 16,366 |
| | MTOI2 | 860 | 5,435 | 6,486 | 1,144 | 7,150 | 8,597 | 1,307 | 7,193 | 9,819 |
| *P. tohiveana* | M5 | 1,306 | 6,452 | 7,931 | 1,770 | 8,567 | 10,592 | 2,034 | 8,755 | 12,277 |
| | TOHM1 | 1,566 | 3,837 | 8,969 | 2,112 | 5,110 | 11,465 | 2,370 | 6,011 | 14,470 |
| *P. affinis* | 73a1 | 1,593 | 3,939 | 8,870 | 2,141 | 5,259 | 11,487 | 2,428 | 6,218 | 14,571 |
| | Y30 | 1,578 | 3,902 | 9,401 | 2,125 | 5,229 | 12,059 | 2,402 | 6,167 | 15,175 |
| | 75a1 | 1,573 | 3,914 | 8,737 | 2,126 | 5,215 | 11,391 | 2,408 | 6,165 | 14,449 |
| | 76a1 | 1,561 | 3,829 | 8,113 | 2,117 | 5,132 | 10,643 | 2,397 | 6,034 | 13,578 |
| | 79a1 | 1,572 | 3,800 | 9,209 | 2,123 | 5,086 | 11,835 | 2,401 | 5,990 | 14,951 |
| | 80a1 | 1,593 | 3,962 | 9,088 | 2,143 | 5,294 | 11,717 | 2,423 | 6,210 | 14,804 |
| | 28a1 | 1,591 | 3,975 | 9,137 | 2,147 | 5,329 | 11,774 | 2,433 | 6,260 | 14,836 |
| | 83a1 | 1,581 | 3,840 | 8,916 | 2,139 | 5,155 | 11,598 | 2,409 | 6,097 | 14,754 |
| | 84a1 | 1,543 | 3,617 | 8,391 | 2,088 | 4,842 | 10,875 | 2,382 | 5,725 | 13,804 |
| | PAZL1 | 1,567 | 3,928 | 9,855 | 2,107 | 5,228 | 12,328 | 2,373 | 6,140 | 15,372 |
| *P. clara* | 26a1 | 1,357 | 6,526 | 8,673 | 1,800 | 8,604 | 11,369 | 2,054 | 8,777 | 13,330 |
| | 46a1 | 1,455 | 7,002 | 9,234 | 1,938 | 9,220 | 12,147 | 2,198 | 9,411 | 14,229 |
| | 46b1 | 1,406 | 6,640 | 8,321 | 1,879 | 8,770 | 10,962 | 2,156 | 8,949 | 12,702 |
| | 48a2 | 1,451 | 6,925 | 8,933 | 1,958 | 9,160 | 11,835 | 2,229 | 9,374 | 13,857 |
| | PCTM | 1,516 | 7,321 | 10,629 | 2,047 | 9,625 | 13,750 | 2,301 | 9,819 | 15,992 |
| *P. c. incrassa* | PCTI | 1,522 | 7,059 | 9,512 | 2,057 | 9,390 | 12,480 | 2,316 | 9,708 | 14,712 |
| *P. diminuta* | 54a4 | 1,031 | 2,788 | 9,549 | 1,434 | 3,704 | 11,765 | 1,714 | 4,271 | 14,415 |
| | 54d2 | 985 | 2,745 | 9,639 | 1,375 | 3,645 | 11,827 | 1,637 | 4,206 | 14,505 |
| *P. hyalina* | 60a1 | 1,334 | 6,674 | 9,051 | 1,798 | 8,788 | 11,874 | 2,054 | 8,972 | 13,863 |
| | PHTH2 | 1,573 | 7,991 | 12,614 | 2,105 | 10,450 | 16,145 | 2,358 | 10,627 | 18,738 |

| | | | | | | | | | | |
|---|---|---|---|---|---|---|---|---|---|---|
| | PHTM | 1,559 | 7,630 | 11,886 | 2,084 | 10,018 | 15,275 | 2,335 | 10,234 | 17,713 |
| | PHRM1 | 1,574 | 7,811 | 12,825 | 2,117 | 10,229 | 16,355 | 2,372 | 10,469 | 19,094 |
| | PHRM314 | 912 | 4,216 | 4,585 | 1,221 | 5,630 | 6,160 | 1,408 | 5,847 | 7,294 |
| *P. h. marmorata* | 41a1 | 1,460 | 7,146 | 9,258 | 1,976 | 9,451 | 12,263 | 2,250 | 9,627 | 14,326 |
| | 43a1 | 1,394 | 6,763 | 9,073 | 1,877 | 8,940 | 11,855 | 2,133 | 9,153 | 13,935 |
| *P. nodosa composita* | Y28 | 1,056 | 2,804 | 9,664 | 1,453 | 3,706 | 11,749 | 1,737 | 4,291 | 14,284 |
| *P. n. intermedia* | PNZL1 | 820 | 2,513 | 9,248 | 1,124 | 3,300 | 11,255 | 1,318 | 3,733 | 13,560 |
| *P. otaheitana* | 49a1 | 1,085 | 2,861 | 9,926 | 1,516 | 3,815 | 12,172 | 1,845 | 4,484 | 15,010 |
| | 39a11 | 1,087 | 2,866 | 9,895 | 1,510 | 3,811 | 12,051 | 1,835 | 4,480 | 14,815 |
| | 72a1 | 1,068 | 2,827 | 9,853 | 1,467 | 3,736 | 12,036 | 1,755 | 4,350 | 14,754 |
| | POZL12 | 674 | 2,355 | 9,019 | 913 | 3,072 | 10,944 | 997 | 3,330 | 13,084 |
| | POZL4 | 1,017 | 2,743 | 9,740 | 1,409 | 3,642 | 11,820 | 1,684 | 4,223 | 14,437 |
| | POMA | 1,054 | 2,821 | 9,781 | 1,455 | 3,718 | 12,034 | 1,770 | 4,351 | 14,774 |
| | POMA1 | 1,119 | 2,873 | 9,812 | 1,535 | 3,797 | 11,997 | 1,860 | 4,489 | 14,764 |
| *P. o. crassa* | Y2-2 | 1,021 | 2,787 | 9,722 | 1,419 | 3,688 | 11,803 | 1,730 | 4,322 | 14,469 |
| | Y35 | 1,000 | 2,747 | 9,532 | 1,382 | 3,656 | 11,667 | 1,661 | 4,221 | 14,315 |
| *P. o. otaheitana* | 93a1 | 1,043 | 2,814 | 9,813 | 1,440 | 3,729 | 12,029 | 1,737 | 4,343 | 14,751 |
| | Y37 | 1,093 | 2,877 | 9,889 | 1,515 | 3,834 | 12,034 | 1,826 | 4,465 | 14,687 |
| | Y36 | 1,046 | 2,811 | 9,825 | 1,458 | 3,756 | 11,941 | 1,751 | 4,362 | 14,577 |
| | 101a1 | 1,473 | 6,984 | 9,583 | 2,001 | 9,244 | 12,691 | 2,275 | 9,499 | 14,719 |
| *P. o. rubescens* | 97b1 | 865 | 2,602 | 9,526 | 1,203 | 3,440 | 11,594 | 1,407 | 3,886 | 14,043 |
| | 98a1 | 1,002 | 2,743 | 9,562 | 1,388 | 3,622 | 11,734 | 1,660 | 4,211 | 14,387 |
| | 99b1 | 1,098 | 2,866 | 9,924 | 1,526 | 3,822 | 12,130 | 1,827 | 4,448 | 14,903 |
| | 99c1 | 1,016 | 2,754 | 9,589 | 1,418 | 3,655 | 11,854 | 1,706 | 4,254 | 14,626 |
| | 100a1 | 1,566 | 7,336 | 10,084 | 2,100 | 9,738 | 13,271 | 2,368 | 9,955 | 15,287 |
| | PRIA | 1,097 | 2,827 | 9,738 | 1,523 | 3,762 | 11,788 | 1,826 | 4,406 | 14,370 |
| *P. o. sinistrorsa* | 109a1 | 1,418 | 6,761 | 8,876 | 1,918 | 8,983 | 11,782 | 2,209 | 9,200 | 13,590 |
| | Y39 | 1,503 | 7,003 | 9,422 | 2,019 | 9,274 | 12,457 | 2,303 | 9,519 | 14,409 |
| | 104b1 | 1,412 | 6,744 | 8,781 | 1,909 | 8,916 | 11,622 | 2,193 | 9,101 | 13,364 |

| | | | | | | | | | | |
|---|---|---|---|---|---|---|---|---|---|---|
| | 104c1 | 1,428 | 6,733 | 8,727 | 1,922 | 8,954 | 11,612 | 2,201 | 9,175 | 13,406 |
| | 110b1 | 1,450 | 6,849 | 9,070 | 1,953 | 9,086 | 12,051 | 2,228 | 9,317 | 13,846 |
| | 111c1 | 1,487 | 6,843 | 8,440 | 2,003 | 9,129 | 11,275 | 2,281 | 9,396 | 12,974 |
| | Y40 | 1,399 | 6,612 | 8,593 | 1,881 | 8,754 | 11,398 | 2,184 | 9,007 | 13,139 |
| | Y18 | 1,543 | 7,338 | 10,429 | 2,081 | 9,748 | 13,787 | 2,350 | 10,011 | 15,962 |
| | Y42 | 1,440 | 6,815 | 9,030 | 1,947 | 9,042 | 11,950 | 2,243 | 9,266 | 13,848 |
| | 106a1 | 1,429 | 6,695 | 8,837 | 1,910 | 8,849 | 11,671 | 2,183 | 9,068 | 13,464 |
| *P. producta* | 108b1 | 1,539 | 7,182 | 9,546 | 2,069 | 9,530 | 12,700 | 2,347 | 9,774 | 14,705 |
| | Y10 | 1,547 | 7,878 | 14,405 | 2,092 | 10,450 | 18,390 | 2,385 | 10,841 | 21,782 |
| | Y44 | 1,521 | 6,982 | 8,909 | 2,044 | 9,279 | 11,876 | 2,336 | 9,478 | 13,646 |
| | 71a1 | 1,081 | 2,849 | 9,867 | 1,504 | 3,787 | 12,069 | 1,800 | 4,406 | 14,799 |
| *P. sp.* | PTMT4 | 950 | 2,682 | 9,547 | 1,299 | 3,535 | 11,724 | 1,567 | 4,082 | 14,398 |
| *P. rosea* | ROSH1 | 1,454 | 6,684 | 8,674 | 1,960 | 8,837 | 11,488 | 2,222 | 9,037 | 13,169 |
| *P. varia* | VARH1 | 1,454 | 6,701 | 8,360 | 1,952 | 8,910 | 11,139 | 2,209 | 9,073 | 12,880 |
| *P. clarkei* | PTUG1 | 1,371 | 5,447 | 5,959 | 1,858 | 7,247 | 7,989 | 2,082 | 7,555 | 9,363 |
| | PTUG2 | 1,426 | 6,078 | 7,722 | 1,928 | 8,147 | 10,321 | 2,166 | 8,514 | 12,203 |
| | PTUG4 | 1,506 | 7,461 | 11,659 | 2,030 | 9,825 | 15,034 | 2,283 | 10,110 | 17,679 |
| *P. faba* | 33a2 | 1,397 | 6,602 | 8,331 | 1,890 | 8,774 | 11,120 | 2,154 | 9,010 | 12,927 |
| | 35a1 | 1,261 | 6,300 | 7,954 | 1,692 | 8,330 | 10,535 | 1,930 | 8,452 | 12,123 |
| | FABR3 | 1,442 | 6,656 | 8,122 | 1,926 | 8,800 | 10,767 | 2,178 | 8,969 | 12,364 |
| *P. garrettii* | TRIR1 | 1,472 | 6,810 | 8,903 | 1,974 | 8,986 | 11,722 | 2,214 | 9,160 | 13,455 |
| *P. hebe* | 31a2 | 1,362 | 6,542 | 8,235 | 1,835 | 8,638 | 10,933 | 2,093 | 8,794 | 12,675 |
| *P. h. bella* | HEBR1 | 1,476 | 6,840 | 8,624 | 1,990 | 9,044 | 11,441 | 2,236 | 9,228 | 13,149 |
| *P. meyeri* | SRMT | 1,444 | 6,693 | 8,477 | 1,937 | 8,862 | 11,307 | 2,215 | 9,087 | 13,090 |
| *P. navigatoria* | DENR1 | 1,485 | 6,864 | 8,896 | 1,994 | 9,090 | 11,761 | 2,268 | 9,285 | 13,585 |
| | DENR2 | 1,441 | 6,668 | 8,367 | 1,934 | 8,831 | 11,071 | 2,201 | 9,036 | 12,817 |
| *P. assimilis* | PHRT1 | 1,520 | 7,220 | 10,080 | 2,051 | 9,592 | 13,312 | 2,292 | 9,800 | 15,370 |
| *P. lutea* | 65a1 | 1,462 | 6,808 | 8,967 | 1,967 | 9,036 | 11,935 | 2,225 | 9,240 | 13,730 |

| | | | | | | | | | | |
|---|---|---|---|---|---|---|---|---|---|---|
| Outgroups | | | | | | | | | | |
| *P. auraniana* | PVT27-1 | 1,338 | 6,037 | 7,438 | 1,784 | 7,868 | 9,740 | 1,903 | 7,462 | 10,205 |
| *P. cramptoni* | B1 | 1,333 | 6,216 | 8,194 | 1,784 | 8,167 | 10,747 | 1,933 | 7,926 | 11,658 |
| | B2 | 1,419 | 6,655 | 8,768 | 1,903 | 8,752 | 11,532 | 2,076 | 8,701 | 12,871 |
| *P. lirata* | PLIR3 | 1,289 | 5,624 | 6,486 | 1,705 | 7,385 | 8,522 | 1,797 | 7,164 | 9,281 |
| | PLIR4 | 1,165 | 4,764 | 5,657 | 1,534 | 6,281 | 7,481 | 1,649 | 6,237 | 8,332 |
| *P. pacifica* | PTUN2 | 463 | 1,116 | 1,718 | 619 | 1,465 | 2,251 | 687 | 1,651 | 2,768 |
| | PTUN4 | 935 | 2,459 | 5,085 | 1,276 | 3,262 | 6,482 | 1,381 | 3,632 | 7,857 |
| | PTUR7 | 535 | 1,289 | 1,371 | 700 | 1,725 | 1,854 | 746 | 1,900 | 2,228 |
| *Samoana attenuata* | SATH | 748 | 3,396 | 5,074 | 896 | 3,922 | 5,852 | 493 | 1,853 | 3,273 |
| *S. burchi* | SS23a1 | 752 | 3,450 | 5,594 | 901 | 4,016 | 6,347 | 514 | 1,940 | 3,683 |
| *S. diaphana* | SD25a1 | 737 | 3,365 | 5,011 | 885 | 3,901 | 5,770 | 504 | 1,877 | 3,221 |

**Table S4** Population assignment values for Moorean and Tahitian *Partula* individuals from the Structure analyses for (a) all 93 Moorean and Tahitian individuals sampled, (b) Clade 1, (c) Clade 2, (d) Clade 2 – Tahiti, and (e) Clade 2 - Moorea. Information for each individual includes species designation, individual id code, and proportion of assignment. Individuals are ordered based on their position in the tree and in Structure bar graphs. Black= 1970 lyophilized individuals collected by J.B. Burch, blue= remnant valley population samples, and red= captive population specimens.

(a) all 93 Moorean and Tahitian individuals

| Clade | Island | Species | Individual | K1 (green) | K2 (orange) | K3 (purple) |
|---|---|---|---|---|---|---|
| 1 | Tahiti | *P. producta* | 71a1 | 0.999 | 0.001 | 0.001 |
| | | *P. affinis* | 73a1 | 0.999 | 0.000 | 0.001 |
| | | *P. o. sinistrorsa* | Y42 | 0.993 | 0.006 | 0.001 |
| | | *P. o. sinistrorsa* | 109a1 | 0.999 | 0.000 | 0.001 |
| | | *P. o. sinistrorsa* | 111c1 | 0.998 | 0.001 | 0.001 |
| | | *P. o. sinistrorsa* | 110b1 | 0.997 | 0.002 | 0.001 |
| | | *P. producta* | Y10 | 0.999 | 0.000 | 0.001 |
| | | *P. producta* | Y44 | 0.999 | 0.000 | 0.001 |
| | | *P. o. sinistrorsa* | Y18 | 0.999 | 0.000 | 0.001 |
| | | *P. o. sinistrorsa* | Y40 | 0.998 | 0.001 | 0.001 |
| | | *P. o. sinistrorsa* | 104b1 | 0.998 | 0.001 | 0.001 |
| | | *P. o. sinistrorsa* | Y39 | 0.999 | 0.000 | 0.001 |
| | | *P. otaheitana* | 49a1 | 0.996 | 0.001 | 0.003 |
| | | *P. o. sinistrorsa* | 104c1 | 0.998 | 0.001 | 0.001 |
| | | *P. o. sinistrorsa* | 106a1 | 0.999 | 0.000 | 0.001 |
| | | *P. affinis* | 28a1 | 0.997 | 0.001 | 0.002 |
| | | *P. o. crassa* | Y35 | 0.997 | 0.001 | 0.002 |
| | | *P. nodosa intermedia* | PNZL1 | 0.998 | 0.001 | 0.001 |
| | | *P. n. composita* | Y28 | 0.999 | 0.000 | 0.001 |
| | | *P. o. crassa* | Y2-2 | 0.997 | 0.001 | 0.002 |
| | | *P. otaheitana* | POZL12 | 0.998 | 0.001 | 0.001 |

| | | | | | | |
|---|---|---|---|---|---|---|
| | | *P. otaheitana* | POZL4 | 0.996 | 0.001 | 0.003 |
| | | *P. sp.* | PTMT4 | 0.997 | 0.001 | 0.002 |
| | | *P. o. otaheitana* | Y36 | 0.998 | 0.000 | 0.002 |
| | | *P. o. otaheitana* | Y37 | 0.999 | 0.000 | 0.001 |
| | | *P. o. otaheitana* | 93a1 | 0.998 | 0.001 | 0.001 |
| | | *P. o. otaheitana* | 101a1 | 0.998 | 0.001 | 0.001 |
| | | *P. otaheitana* | POMA | 0.998 | 0.001 | 0.001 |
| | | *P. otaheitana* | POMA1 | 0.994 | 0.004 | 0.002 |
| | | *P. diminuta* | 54a4 | 0.997 | 0.001 | 0.002 |
| | | *P. diminuta* | 54d2 | 0.943 | 0.052 | 0.004 |
| | | *P. affinis* | 79a1 | 0.996 | 0.001 | 0.003 |
| | | *P. affinis* | 76a1 | 0.998 | 0.000 | 0.002 |
| | | *P. affinis* | Y30 | 0.997 | 0.001 | 0.002 |
| | | *P. affinis* | 75a1 | 0.994 | 0.001 | 0.005 |
| | | *P. affinis* | 80a1 | 0.999 | 0.000 | 0.001 |
| | | *P. o. rubescens* | 97b1 | 0.998 | 0.001 | 0.001 |
| | | *P. producta* | 108b1 | 0.997 | 0.001 | 0.002 |
| | | *P. otaheitana* | 39a11 | 0.992 | 0.001 | 0.008 |
| | | *P. o. rubescens* | PRIA | 0.998 | 0.001 | 0.001 |
| | | *P. affinis* | PAZL1 | 0.999 | 0.000 | 0.001 |
| | | *P. o. rubescens* | 100a1 | 0.999 | 0.000 | 0.001 |
| | | *P. otaheitana* | 72a1 | 0.999 | 0.000 | 0.001 |
| | | *P. o. rubescens* | 99b1 | 0.999 | 0.000 | 0.001 |
| | | *P. o. rubescens* | 99c1 | 0.999 | 0.000 | 0.001 |
| | | *P. affinis* | 84a1 | 0.995 | 0.001 | 0.004 |
| | | *P. o. rubescens* | 98a1 | 0.998 | 0.001 | 0.001 |
| | | *P. affinis* | 83a1 | 0.995 | 0.000 | 0.005 |
| 2 | Tahiti | *P. hyalina* | PHRM314 | 0.000 | 1.000 | 0.000 |
| | | *P. h. marmorata* | 43a1 | 0.000 | 1.000 | 0.000 |
| | | *P. h. marmorata* | 41a1 | 0.000 | 1.000 | 0.000 |
| | | *P. hyalina* | 60a1 | 0.000 | 1.000 | 0.000 |

| | | | | | | |
|---|---|---|---|---|---|---|
| | | *P. hyalina* | PHTH | 0.000 | 1.000 | 0.000 |
| | | *P. hyalina* | PHTM | 0.000 | 1.000 | 0.000 |
| | | *P. hyalina* | PHRM1 | 0.000 | 1.000 | 0.000 |
| | | *P. clara* | PCTM | 0.000 | 1.000 | 0.000 |
| | | *P. c. incrassa* | PCTI | 0.000 | 1.000 | 0.000 |
| | | *P. clara* | 48a2 | 0.000 | 0.999 | 0.001 |
| | | *P. clara* | 46b1 | 0.000 | 1.000 | 0.000 |
| | | *P. clara* | 46a1 | 0.002 | 0.997 | 0.001 |
| | | *P. clara* | 26a1 | 0.002 | 0.997 | 0.001 |
| 2 | Moorea | *P. taeniata* | 131b1 | 0.001 | 0.115 | 0.884 |
| | | *P. exigua* | 132b1 | 0.001 | 0.065 | 0.934 |
| | | *P. taeniata* | PS136a1 | 0.001 | 0.137 | 0.862 |
| | | *P. exigua* | 51a1 | 0.001 | 0.275 | 0.724 |
| | | *P. taeniata* | PHAU | 0.002 | 0.259 | 0.739 |
| | | *P. mirabilis* | 130d1 | 0.001 | 0.005 | 0.994 |
| | | *P. taeniata* | 130c1 | 0.001 | 0.020 | 0.979 |
| | | *P. taeniata* | 130a1 | 0.001 | 0.035 | 0.964 |
| | | *P. taeniata* | TAEH1 | 0.001 | 0.160 | 0.839 |
| | | *P. taeniata* | NUCM1 | 0.001 | 0.036 | 0.963 |
| | | *P. taeniata* | M10 | 0.004 | 0.143 | 0.853 |
| | | *P. mirabilis* | M9 | 0.002 | 0.002 | 0.996 |
| | | *P. tohiveana* | TOHM1 | 0.001 | 0.001 | 0.998 |
| | | *P. tohiveana* | M5 | 0.001 | 0.001 | 0.998 |
| | | *P. mooreana* | MOOM2 | 0.001 | 0.000 | 0.999 |
| | | *P. taeniata* | MTO12 | 0.001 | 0.001 | 0.998 |
| | | *P. suturalis suturalis* | 123b1 | 0.001 | 0.001 | 0.998 |
| | | *P. suturalis* | 118d1 | 0.001 | 0.002 | 0.997 |
| | | *P. s. suturalis* | 121d1 | 0.001 | 0.000 | 1.000 |
| | | *P. s. vexillum* | 50b1 | 0.002 | 0.001 | 0.997 |
| | | *P. s. vexillum* | VEXM1 | 0.001 | 0.001 | 0.998 |
| | | *P. s. vexillum* | 127a1 | 0.001 | 0.000 | 0.999 |

| | | | | |
|---|---|---|---|---|
| *P. m. propinqua* | 135a3 | 0.001 | 0.001 | 0.998 |
| *P. s. vexillum* | 126c1 | 0.006 | 0.000 | 0.994 |
| *P. suturalis* | 116a1 | 0.001 | 0.000 | 0.999 |
| *P. aurantia* | M1 | 0.001 | 0.001 | 0.998 |
| *P. suturalis* | 118b1 | 0.001 | 0.000 | 0.999 |
| *P. s. vexillum* | M7 | 0.002 | 0.001 | 0.997 |
| *P. s. vexillum* | VEXM2 | 0.001 | 0.000 | 0.999 |
| *P. s. suturalis* | STRM1 | 0.002 | 0.000 | 0.998 |
| *P. mirabilis* | PM67a1 | 0.027 | 0.001 | 0.972 |
| *P. mirabilis* | PMIZ1 | 0.001 | 0.000 | 0.999 |

(b) Clade 1

| Complex | Species | Individual | Two populations | | Three populations | | |
|---|---|---|---|---|---|---|---|
| | | | K1 (lt. green) | K2 (dk. green) | K1 (lt. green) | K2 (blue) | K3 (dk. green) |
| "*P. otaheitana*" A | *P. producta* | 71a1 | 0.569 | 0.431 | 0.882 | 0.004 | 0.113 |
| "*P. otaheitana*" A | *P. affinis* | 73a1 | 0.564 | 0.436 | 0.741 | 0.006 | 0.253 |
| "*P. otaheitana*" A | *P. o. sinistrorsa* | Y42 | 0.963 | 0.037 | 0.987 | 0.005 | 0.007 |
| "*P. otaheitana*" A | *P. o. sinistrorsa* | 109a1 | 0.644 | 0.356 | 0.765 | 0.007 | 0.228 |
| "*P. otaheitana*" A | *P. o. sinistrorsa* | 111c1 | 0.992 | 0.008 | 0.992 | 0.004 | 0.004 |
| "*P. otaheitana*" A | *P. o. sinistrorsa* | 110b1 | 0.992 | 0.008 | 0.989 | 0.007 | 0.004 |
| "*P. otaheitana*" A | *P. producta* | Y10 | 0.967 | 0.033 | 0.990 | 0.002 | 0.008 |
| "*P. otaheitana*" A | *P. producta* | Y44 | 0.994 | 0.006 | 0.996 | 0.002 | 0.002 |
| "*P. otaheitana*" A | *P. o. sinistrorsa* | Y18 | 0.989 | 0.011 | 0.972 | 0.017 | 0.011 |
| "*P. otaheitana*" A | *P. o. sinistrorsa* | Y40 | 0.994 | 0.006 | 0.904 | 0.090 | 0.006 |
| "*P. otaheitana*" A | *P. o. sinistrorsa* | 104b1 | 0.995 | 0.005 | 0.890 | 0.107 | 0.003 |
| "*P. otaheitana*" A | *P. o. sinistrorsa* | Y39 | 0.996 | 0.004 | 0.988 | 0.009 | 0.003 |
| "*P. otaheitana*" A | *P. otaheitana* | 49a1 | 0.993 | 0.008 | 0.992 | 0.004 | 0.004 |
| "*P. otaheitana*" A | *P. o. sinistrorsa* | 104c1 | 0.996 | 0.004 | 0.993 | 0.004 | 0.003 |

| | | | | | | | |
|---|---|---|---|---|---|---|---|
| "*P. otaheitana*" A | *P. o. sinistrorsa* | 106a1 | 0.997 | 0.003 | 0.595 | 0.402 | 0.003 |
| "*P. otaheitana*" A | *P. affinis* | 28a1 | 0.993 | 0.007 | 0.738 | 0.257 | 0.005 |
| "*P. otaheitana*" A | *P. o. crassa* | Y35 | 0.988 | 0.013 | 0.811 | 0.181 | 0.008 |
| "*P. otaheitana*" A | *P. nodosa intermedia* | PNZL1 | 0.995 | 0.005 | 0.738 | 0.258 | 0.004 |
| "*P. otaheitana*" B | *P. n. composita* | Y28 | 0.996 | 0.004 | 0.459 | 0.538 | 0.003 |
| "*P. otaheitana*" B | *P. o. crassa* | Y2-2 | 0.971 | 0.030 | 0.108 | 0.869 | 0.023 |
| "*P. otaheitana*" B | *P. otaheitana* | POZL12 | 0.994 | 0.006 | 0.018 | 0.978 | 0.004 |
| "*P. otaheitana*" B | *P. otaheitana* | POZL4 | 0.994 | 0.006 | 0.039 | 0.957 | 0.004 |
| "*P. otaheitana*" B | *P. sp.* | PTMT4 | 0.992 | 0.008 | 0.015 | 0.979 | 0.006 |
| "*P. otaheitana*" B | *P. o. otaheitana* | Y36 | 0.993 | 0.007 | 0.019 | 0.975 | 0.005 |
| "*P. otaheitana*" B | *P. o. otaheitana* | Y37 | 0.987 | 0.013 | 0.018 | 0.975 | 0.007 |
| "*P. otaheitana*" B | *P. o. otaheitana* | 93a1 | 0.996 | 0.004 | 0.018 | 0.979 | 0.003 |
| "*P. otaheitana*" B | *P. o. otaheitana* | 101a1 | 0.944 | 0.056 | 0.030 | 0.957 | 0.014 |
| "*P. otaheitana*" B | *P. otaheitana* | POMA | 0.987 | 0.013 | 0.010 | 0.983 | 0.007 |
| "*P. otaheitana*" B | *P. otaheitana* | POMA1 | 0.943 | 0.057 | 0.005 | 0.987 | 0.008 |
| "*P. otaheitana*" B | *P. diminuta* | 54a4 | 0.993 | 0.007 | 0.008 | 0.989 | 0.003 |
| "*P. otaheitana*" B | *P. diminuta* | 54d2 | 0.991 | 0.009 | 0.006 | 0.989 | 0.005 |
| "*P. affinis*" | *P. affinis* | 79a1 | 0.006 | 0.994 | 0.006 | 0.003 | 0.991 |
| "*P. affinis*" | *P. affinis* | 76a1 | 0.005 | 0.995 | 0.005 | 0.003 | 0.992 |
| "*P. affinis*" | *P. affinis* | Y30 | 0.004 | 0.996 | 0.005 | 0.002 | 0.993 |
| "*P. affinis*" | *P. affinis* | 75a1 | 0.004 | 0.996 | 0.004 | 0.003 | 0.993 |
| "*P. affinis*" | *P. affinis* | 80a1 | 0.329 | 0.671 | 0.487 | 0.004 | 0.509 |
| "*P. affinis*" | *P. o. rubescens* | 97b1 | 0.140 | 0.861 | 0.488 | 0.004 | 0.508 |
| "*P. affinis*" | *P. producta* | 108b1 | 0.121 | 0.879 | 0.195 | 0.021 | 0.784 |
| "*P. affinis*" | *P. otaheitana* | 39a11 | 0.006 | 0.994 | 0.006 | 0.020 | 0.975 |
| "*P. affinis*" | *P. o. rubescens* | PRIA | 0.015 | 0.985 | 0.019 | 0.011 | 0.970 |
| "*P. affinis*" | *P. affinis* | PAZL1 | 0.005 | 0.995 | 0.006 | 0.032 | 0.962 |
| "*P. affinis*" | *P. o. rubescens* | 100a1 | 0.040 | 0.960 | 0.060 | 0.054 | 0.886 |
| "*P. affinis*" | *P. otaheitana* | 72a1 | 0.015 | 0.985 | 0.167 | 0.008 | 0.826 |

| | | | | | | | |
|---|---|---|---|---|---|---|---|
| *"P. affinis"* | *P. o. rubescens* | 99b1 | 0.316 | 0.684 | 0.007 | 0.614 | 0.379 |
| *"P. affinis"* | *P. o. rubescens* | 99c1 | 0.010 | 0.990 | 0.004 | 0.297 | 0.699 |
| *"P. affinis"* | *P. affinis* | 84a1 | 0.524 | 0.476 | 0.007 | 0.813 | 0.180 |
| *"P. affinis"* | *P. o. rubescens* | 98a1 | 0.092 | 0.908 | 0.052 | 0.368 | 0.580 |
| *"P. affinis"* | *P. affinis* | 83a1 | 0.303 | 0.697 | 0.011 | 0.697 | 0.293 |

(c) Clade 2

| | | | | Two populations | | Three populations | | |
|---|---|---|---|---|---|---|---|---|
| Island | Complex | Species | Individual | K1 (orange) | K2 (purple) | K1 (orange) | K2 (pink) | K3 (purple) |
| Tahiti | *"P. clara/P. hyalina"* | *P. hyalina* | PHRM314 | 0.999 | 0.001 | 0.998 | 0.001 | 0.001 |
| | *"P. clara/P. hyalina"* | *P. h. marmorata* | 43a1 | 0.999 | 0.001 | 0.999 | 0.001 | 0.000 |
| | *"P. clara/P. hyalina"* | *P. h. marmorata* | 41a1 | 0.999 | 0.001 | 1.000 | 0.001 | 0.000 |
| | *"P. clara/P. hyalina"* | *P. hyalina* | 60a1 | 0.999 | 0.001 | 0.999 | 0.001 | 0.000 |
| | *"P. clara/P. hyalina"* | *P. hyalina* | PHTH | 1.000 | 0.000 | 1.000 | 0.000 | 0.000 |
| | *"P. clara/P. hyalina"* | *P. hyalina* | PHTM | 0.999 | 0.001 | 0.999 | 0.001 | 0.000 |
| | *"P. clara/P. hyalina"* | *P. hyalina* | PHRM1 | 0.999 | 0.001 | 0.998 | 0.001 | 0.001 |
| | *"P. clara/P. hyalina"* | *P. clara* | PCTM | 0.999 | 0.001 | 0.999 | 0.001 | 0.000 |
| | *"P. clara/P. hyalina"* | *P. c. incrassa* | PCTI | 0.999 | 0.001 | 0.999 | 0.001 | 0.000 |
| | *"P. clara/P. hyalina"* | *P. clara* | 48a2 | 0.999 | 0.001 | 0.996 | 0.003 | 0.001 |
| | *"P. clara/P. hyalina"* | *P. clara* | 46b1 | 0.999 | 0.001 | 0.999 | 0.001 | 0.000 |
| | *"P. clara/P. hyalina"* | *P. clara* | 46a1 | 0.998 | 0.002 | 0.997 | 0.002 | 0.001 |
| | *"P. clara/P. hyalina"* | *P. clara* | 26a1 | 0.999 | 0.002 | 0.997 | 0.002 | 0.001 |
| Moorea | *"P. taeniata"* | *P. taeniata* | 131b1 | 0.099 | 0.901 | 0.003 | 0.884 | 0.113 |
| | *"P. taeniata"* | *P. exigua* | 132b1 | 0.047 | 0.953 | 0.001 | 0.872 | 0.127 |
| | *"P. taeniata"* | *P. taeniata* | PS136a1 | 0.125 | 0.875 | 0.001 | 0.992 | 0.006 |
| | *"P. taeniata"* | *P. exigua* | 51a1 | 0.273 | 0.727 | 0.011 | 0.988 | 0.001 |
| | *"P. taeniata"* | *P. taeniata* | PHAU | 0.241 | 0.759 | 0.009 | 0.990 | 0.001 |
| | *"P. taeniata"* | *P. mirabilis* | 130d1 | 0.004 | 0.996 | 0.001 | 0.924 | 0.075 |

| | | | | | | | |
|---|---|---|---|---|---|---|---|
| "*P. taeniata*" | *P. taeniata* | 130c1 | 0.014 | 0.986 | 0.001 | 0.997 | 0.002 |
| "*P. taeniata*" | *P. taeniata* | 130a1 | 0.022 | 0.978 | 0.001 | 0.997 | 0.002 |
| "*P. taeniata*" | *P. taeniata* | TAEH1 | 0.148 | 0.853 | 0.031 | 0.932 | 0.037 |
| "*P. taeniata*" | *P. taeniata* | NUCM1 | 0.022 | 0.978 | 0.002 | 0.960 | 0.038 |
| "*P. taeniata*" | *P. taeniata* | M10 | 0.133 | 0.867 | 0.074 | 0.425 | 0.501 |
| "*P. taeniata*" | *P. mirabilis* | M9 | 0.003 | 0.997 | 0.002 | 0.549 | 0.449 |
| "*P. suturalis*" | *P. tohiveana* | TOHM1 | 0.001 | 0.999 | 0.001 | 0.025 | 0.974 |
| "*P. suturalis*" | *P. tohiveana* | M5 | 0.002 | 0.998 | 0.002 | 0.012 | 0.986 |
| "*P. suturalis*" | *P. mooreana* | MOOM2 | 0.001 | 0.999 | 0.001 | 0.022 | 0.977 |
| "*P. suturalis*" | *P. taeniata* | MTO12 | 0.002 | 0.998 | 0.001 | 0.024 | 0.975 |
| "*P. suturalis*" | *P. suturalis suturalis* | 123b1 | 0.001 | 0.999 | 0.001 | 0.004 | 0.995 |
| "*P. suturalis*" | *P. suturalis* | 118d1 | 0.002 | 0.998 | 0.001 | 0.001 | 0.998 |
| "*P. suturalis*" | *P. s. suturalis* | 121d1 | 0.001 | 0.999 | 0.001 | 0.003 | 0.996 |
| "*P. suturalis*" | *P. s. vexillum* | 50b1 | 0.001 | 0.999 | 0.001 | 0.001 | 0.998 |
| "*P. suturalis*" | *P. s. vexillum* | VEXM1 | 0.001 | 0.999 | 0.001 | 0.001 | 0.998 |
| "*P. suturalis*" | *P. s. vexillum* | 127a1 | 0.001 | 0.999 | 0.001 | 0.001 | 0.998 |
| "*P. suturalis*" | *P. m. propinqua* | 135a3 | 0.002 | 0.998 | 0.002 | 0.009 | 0.989 |
| "*P. suturalis*" | *P. s. vexillum* | 126c1 | 0.001 | 0.999 | 0.001 | 0.002 | 0.997 |
| "*P. suturalis*" | *P. suturalis* | 116a1 | 0.001 | 0.999 | 0.001 | 0.001 | 0.998 |
| "*P. suturalis*" | *P. aurantia* | M1 | 0.001 | 0.999 | 0.001 | 0.006 | 0.993 |
| "*P. suturalis*" | *P. suturalis* | 118b1 | 0.001 | 0.999 | 0.001 | 0.002 | 0.997 |
| "*P. suturalis*" | *P. s. vexillum* | M7 | 0.001 | 0.999 | 0.001 | 0.003 | 0.996 |
| "*P. suturalis*" | *P. s. vexillum* | VEXM2 | 0.001 | 0.999 | 0.001 | 0.001 | 0.998 |
| "*P. suturalis*" | *P. s. suturalis* | STRM1 | 0.001 | 0.999 | 0.001 | 0.001 | 0.998 |
| "*P. suturalis*" | *P. mirabilis* | PM67a1 | 0.001 | 0.999 | 0.001 | 0.002 | 0.997 |
| "*P. suturalis*" | *P. mirabilis* | PMIZ1 | 0.001 | 0.999 | 0.001 | 0.001 | 0.998 |

(d) Clade 2 - Tahiti

| Complex | Species | Individual | K1 (orange) | K2 (yellow) | K3 (red) |
|---|---|---|---|---|---|
| *"P. clara/P. hyalina"* A | *P. hyalina* | PHRM314 | 0.997 | 0.002 | 0.001 |
| *"P. clara/P. hyalina"* A | *P. h. marmorata* | 43a1 | 0.998 | 0.001 | 0.001 |
| *"P. clara/P. hyalina"* A | *P. h. marmorata* | 41a1 | 0.997 | 0.002 | 0.001 |
| *"P. clara/P. hyalina"* A | *P. hyalina* | 60a1 | 0.988 | 0.009 | 0.003 |
| *"P. clara/P. hyalina"* A | *P. hyalina* | PHTH | 0.998 | 0.001 | 0.001 |
| *"P. clara/P. hyalina"* A | *P. hyalina* | PHTM | 0.707 | 0.291 | 0.002 |
| *"P. clara/P. hyalina"* A | *P. hyalina* | PHRM1 | 0.527 | 0.471 | 0.003 |
| *"P. clara/P. hyalina"* B | *P. clara* | PCTM | 0.001 | 0.998 | 0.001 |
| *"P. clara/P. hyalina"* B | *P. c. incrassa* | PCTI | 0.001 | 0.998 | 0.001 |
| *"P. clara/P. hyalina"* B | *P. clara* | 48a2 | 0.004 | 0.991 | 0.005 |
| *"P. clara/P. hyalina"* C | *P. clara* | 46b1 | 0.001 | 0.001 | 0.998 |
| *"P. clara/P. hyalina"* C | *P. clara* | 46a1 | 0.001 | 0.003 | 0.996 |
| *"P. clara/P. hyalina"* C | *P. clara* | 26a1 | 0.001 | 0.002 | 0.997 |

(e) Clade 2 - Moorea

| Complex | Species | Individual | K1 (pink) | K2 (purple) | K3 (black) |
|---|---|---|---|---|---|
| *"P. taeniata"* | *P. taeniata* | 131b1 | 0.718 | 0.206 | 0.076 |
| *"P. taeniata"* | *P. exigua* | 132b1 | 0.670 | 0.279 | 0.051 |
| *"P. taeniata"* | *P. taeniata* | PS136a1 | 0.855 | 0.125 | 0.020 |
| *"P. taeniata"* | *P. exigua* | 51a1 | 0.960 | 0.025 | 0.015 |
| *"P. taeniata"* | *P. taeniata* | PHAU | 0.897 | 0.029 | 0.074 |
| *"P. taeniata"* | *P. mirabilis* | 130d1 | 0.559 | 0.361 | 0.079 |
| *"P. taeniata"* | *P. taeniata* | 130c1 | 0.780 | 0.170 | 0.050 |
| *"P. taeniata"* | *P. taeniata* | 130a1 | 0.824 | 0.134 | 0.042 |
| *"P. taeniata"* | *P. taeniata* | TAEH1 | 0.628 | 0.291 | 0.081 |
| *"P. taeniata"* | *P. taeniata* | NUCM1 | 0.527 | 0.340 | 0.133 |
| *"P. taeniata"* | *P. taeniata* | M10 | 0.237 | 0.683 | 0.080 |
| *"P. taeniata"* | *P. mirabilis* | M9 | 0.229 | 0.718 | 0.053 |
| *"P. suturalis"* | *P. tohiveana* | TOHM1 | 0.091 | 0.802 | 0.107 |
| *"P. suturalis"* | *P. tohiveana* | M5 | 0.051 | 0.879 | 0.071 |
| *"P. suturalis"* | *P. mooreana* | MOOM2 | 0.071 | 0.783 | 0.146 |
| *"P. suturalis"* | *P. taeniata* | MTO12 | 0.036 | 0.859 | 0.105 |
| *"P. suturalis"* | *P. suturalis suturalis* | 123b1 | 0.013 | 0.928 | 0.059 |
| *"P. suturalis"* | *P. suturalis* | 118d1 | 0.008 | 0.835 | 0.157 |
| *"P. suturalis"* | *P. s. suturalis* | 121d1 | 0.014 | 0.811 | 0.176 |
| *"P. suturalis"* | *P. s. vexillum* | 50b1 | 0.009 | 0.895 | 0.096 |

| | | | | | |
|---|---|---|---|---|---|
| *“P. suturalis”* | *P. s. vexillum* | VEXM1 | 0.008 | 0.886 | 0.106 |
| *“P. suturalis”* | *P. s. vexillum* | 127a1 | 0.008 | 0.861 | 0.131 |
| *“P. suturalis”* | *P. m. propinqua* | 135a3 | 0.030 | 0.865 | 0.105 |
| *“P. suturalis”* | *P. s. vexillum* | 126c1 | 0.013 | 0.820 | 0.167 |
| *“P. suturalis”* | *P. suturalis* | 116a1 | 0.007 | 0.868 | 0.125 |
| *“P. suturalis”* | *P. aurantia* | M1 | 0.016 | 0.927 | 0.057 |
| *“P. suturalis”* | *P. suturalis* | 118b1 | 0.012 | 0.822 | 0.166 |
| *“P. suturalis”* | *P. s. vexillum* | M7 | 0.014 | 0.886 | 0.100 |
| *“P. suturalis”* | *P. s. vexillum* | VEXM2 | 0.008 | 0.887 | 0.105 |
| *“P. suturalis”* | *P. s. suturalis* | STRM1 | 0.010 | 0.886 | 0.104 |
| *“P. suturalis”* | *P. mirabilis* | PM67a1 | 0.013 | 0.869 | 0.119 |
| *“P. suturalis”* | *P. mirabilis* | PMIZ1 | 0.020 | 0.864 | 0.116 |

**Table S5** Results of the $f_3$ admixture tests for the Moorean and Tahitian *Partula* species complexes. Species complex labels are same as Table S1 and Figure S1. The $f_3$ test is in the form (A;B,C) where A is the result of admixture between B and C (Pickrell & Pritchard, 2012; Reich, Thangaraj, Patterson, Price, & Singh, 2009).

| Test | $f_3$ statistic ± s.e. | *Z*-score | *p*-value |
|---|---|---|---|
| *"P. otaheitana"*; *"P. affinis"*, *"P. clara/P. hyalina*" C | -0.0007 ± 0.0002 | -3.9161 | 9.00E-05 |
| *"P. affinis"*; *"P. otaheitana"*, *"P. suturalis"* | -0.0011 ± 0.0001 | -11.1838 | 4.89E-29 |
| *"P. affinis"*; *"P. clara/P. hyalina*" C, *"P. suturalis"* | -0.0005 ± 0.002 | -2.3010 | 0.0214 |
| *"P. taeniata"*; *"P. clara/P. hyalina*" C, *"P. suturalis"* | -0.0010 ± 0.0003 | -3.7629 | 0.0002 |

# Figure S1 (a) figure caption can be found on page 35.

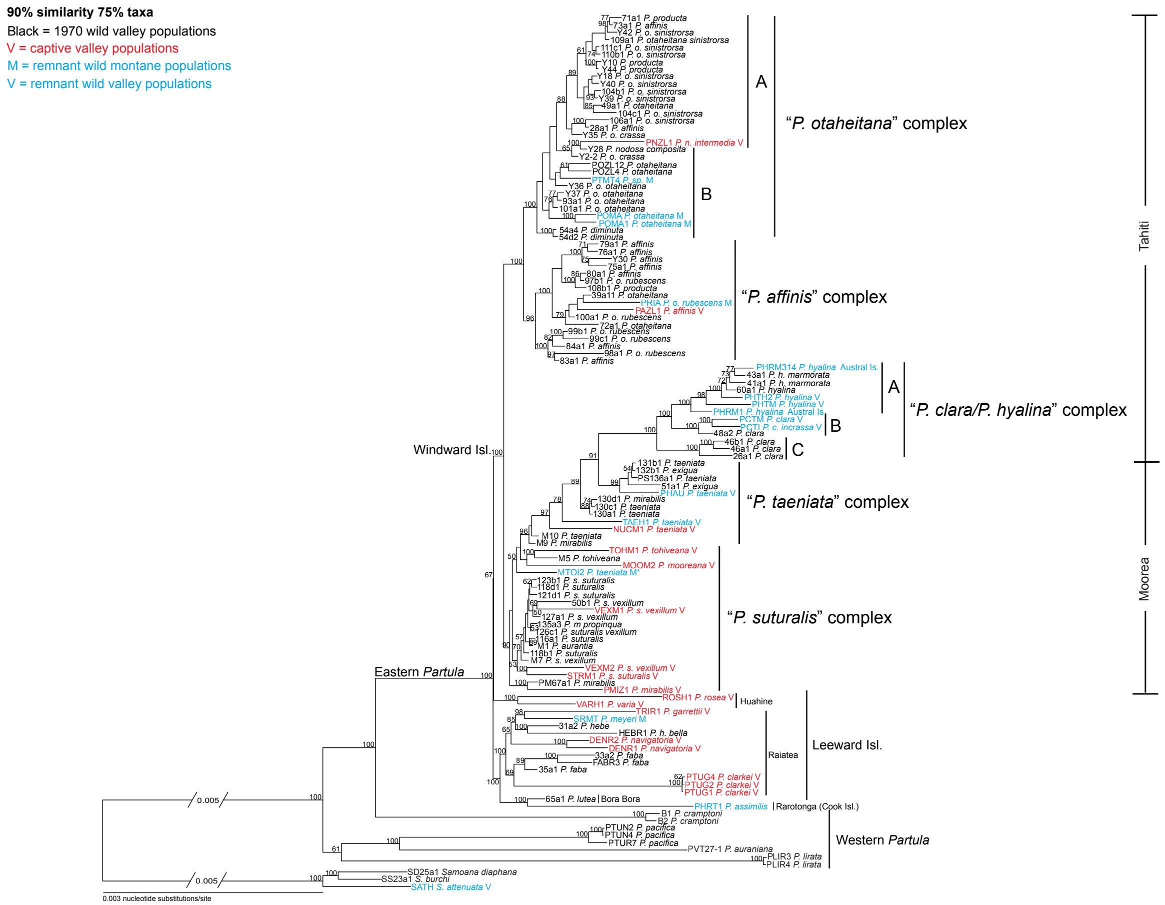

# Figure S1 (b)

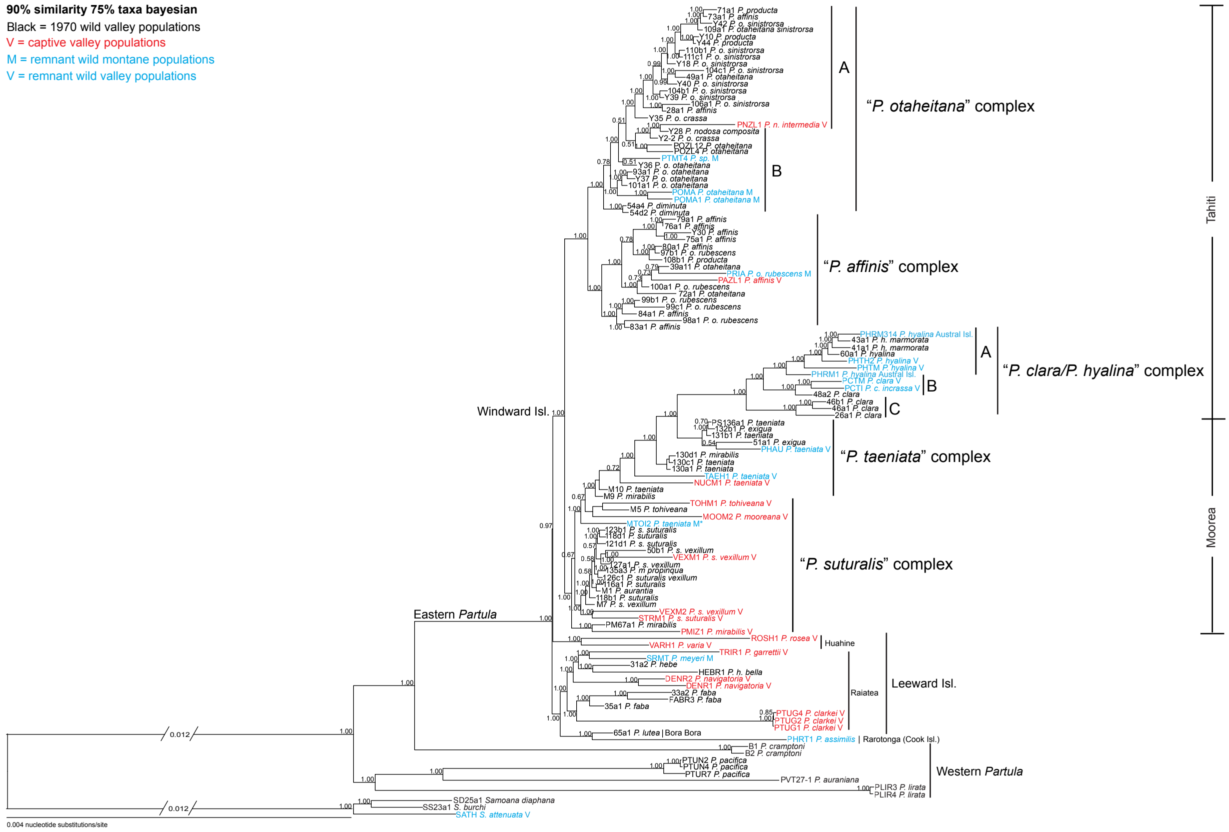
90% similarity 75% taxa bayesian
Black = 1970 wild valley populations
V = captive valley populations
M = remnant wild montane populations
V = remnant wild valley populations
"P. otaheitana" complex
A
B
"P. affinis" complex
"P. clara/P. hyalina" complex
A
B
C
"P. taeniata" complex
"P. suturalis" complex
Tahiti
Moorea
Windward Isl.
Eastern Partula
Huahine
Raiatea
Leeward Isl.
Bora Bora
Rarotonga (Cook Isl.)
Western Partula
0.012
0.004 nucleotide substitutions/site

# Figure S1 (c)

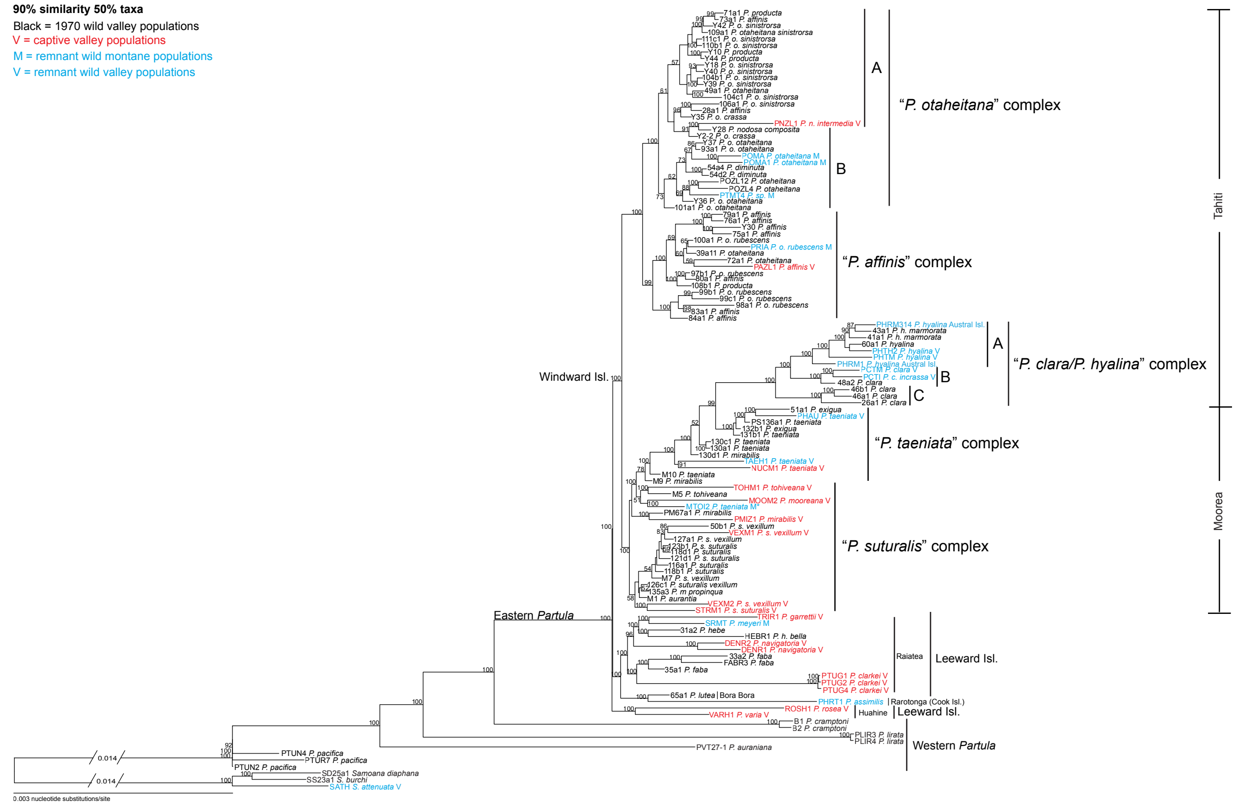
90% similarity 50% taxa
Black = 1970 wild valley populations
V = captive valley populations
M = remnant wild montane populations
V = remnant wild valley populations
A
"P. otaheitana" complex
B
"P. affinis" complex
A
"P. clara/P. hyalina" complex
B
C
"P. taeniata" complex
"P. suturalis" complex
Tahiti
Moorea
Windward Isl.
Eastern Partula
Raiatea
Leeward Isl.
Bora Bora
Rarotonga (Cook Isl.)
Huahine
Leeward Isl.
Western Partula
0.003 nucleotide substitutions/site

# Figure S1 (d)

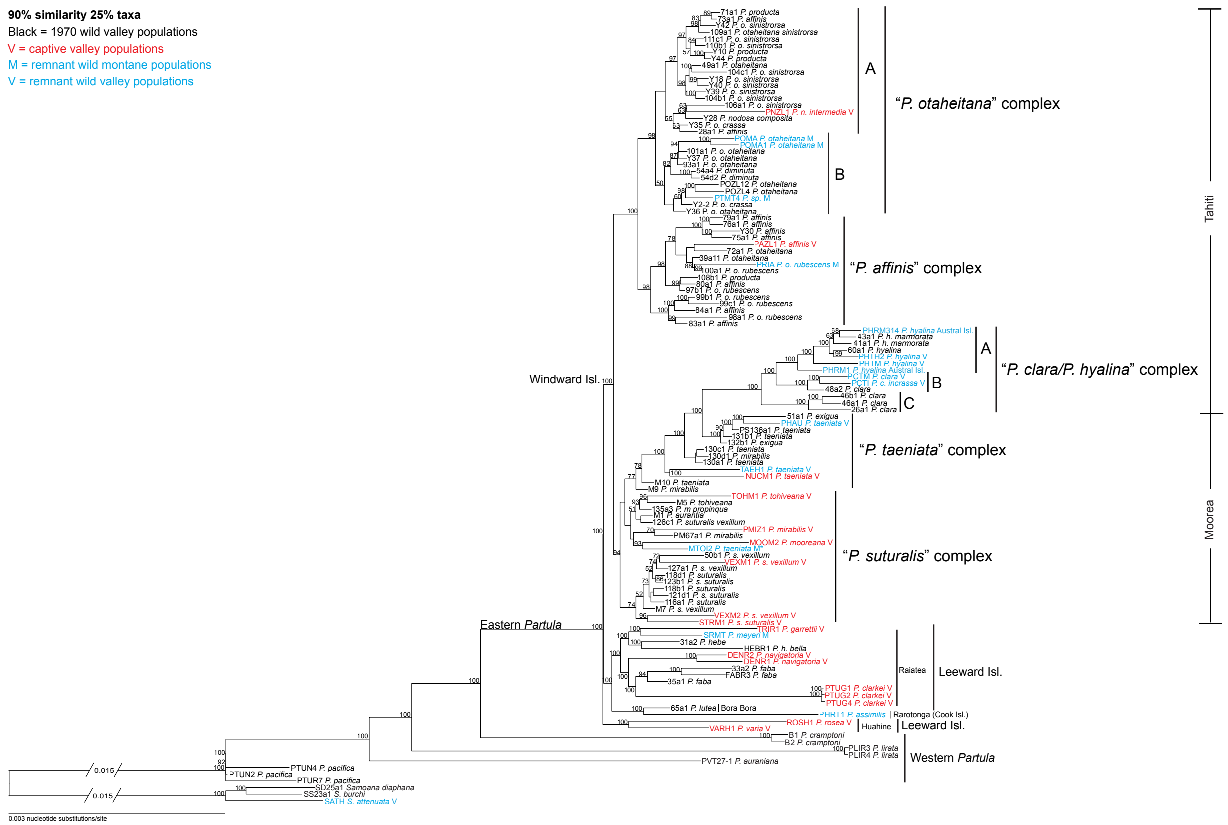

**90% similarity 25% taxa**
Black = 1970 wild valley populations
V = captive valley populations
M = remnant wild montane populations
V = remnant wild valley populations
A
"*P. otaheitana*" complex
B
"*P. affinis*" complex
A
"*P. clara/P. hyalina*" complex
B
C
"*P. taeniata*" complex
"*P. suturalis*" complex
Tahiti
Moorea
Windward Isl.
Eastern *Partula*
Raiatea
Leeward Isl.
Bora Bora
Rarotonga (Cook Isl.)
Huahine
Leeward Isl.
Western *Partula*
0.015
0.015
0.003 nucleotide substitutions/site

# Figure S1 (e)

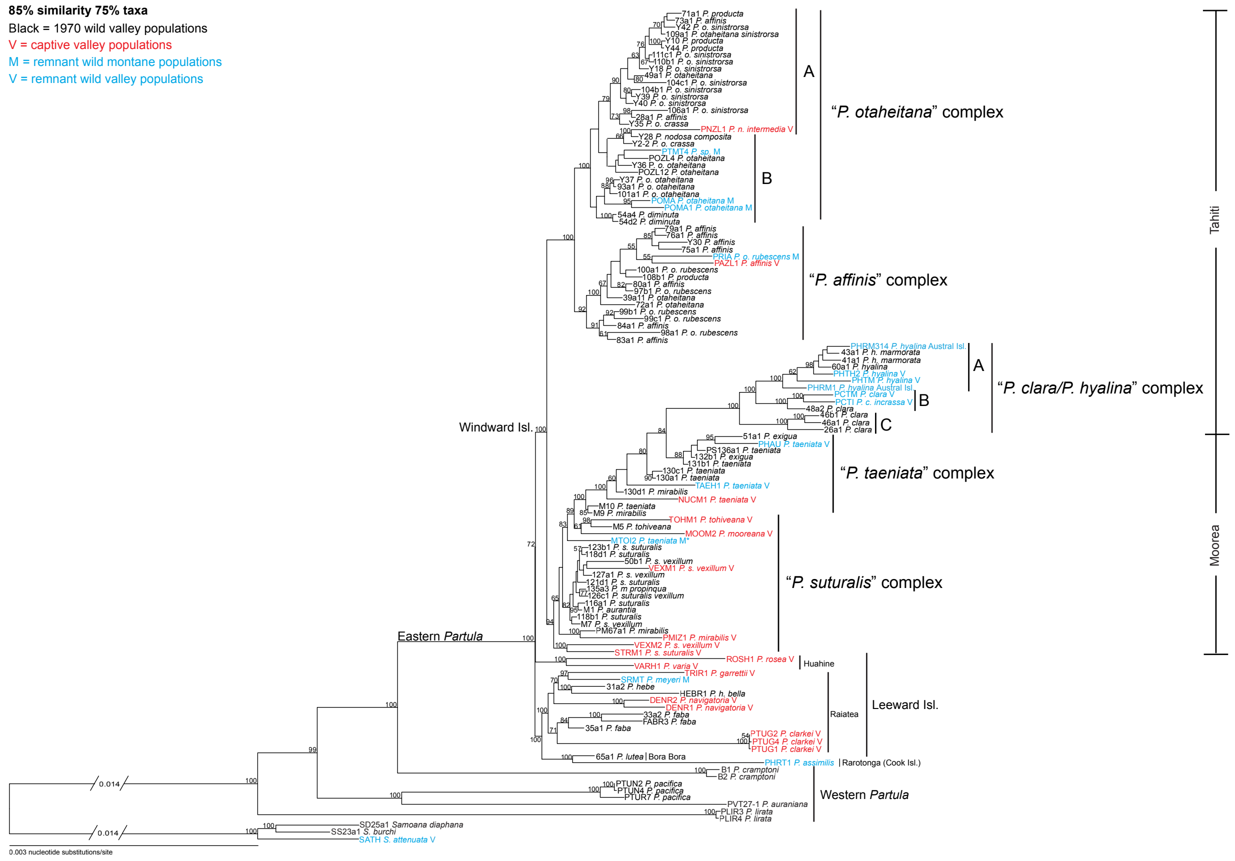
**85% similarity 75% taxa**
Black = 1970 wild valley populations
V = captive valley populations
M = remnant wild montane populations
V = remnant wild valley populations
A
B
"P. otaheitana" complex
"P. affinis" complex
A
B
C
"P. clara/P. hyalina" complex
"P. taeniata" complex
"P. suturalis" complex
Tahiti
Moorea
Windward Isl.
Eastern Partula
Huahine
Raiatea
Leeward Isl.
Bora Bora
Rarotonga (Cook Isl.)
Western Partula
0.014
0.014
0.003 nucleotide substitutions/site

# Figure S1 (f)

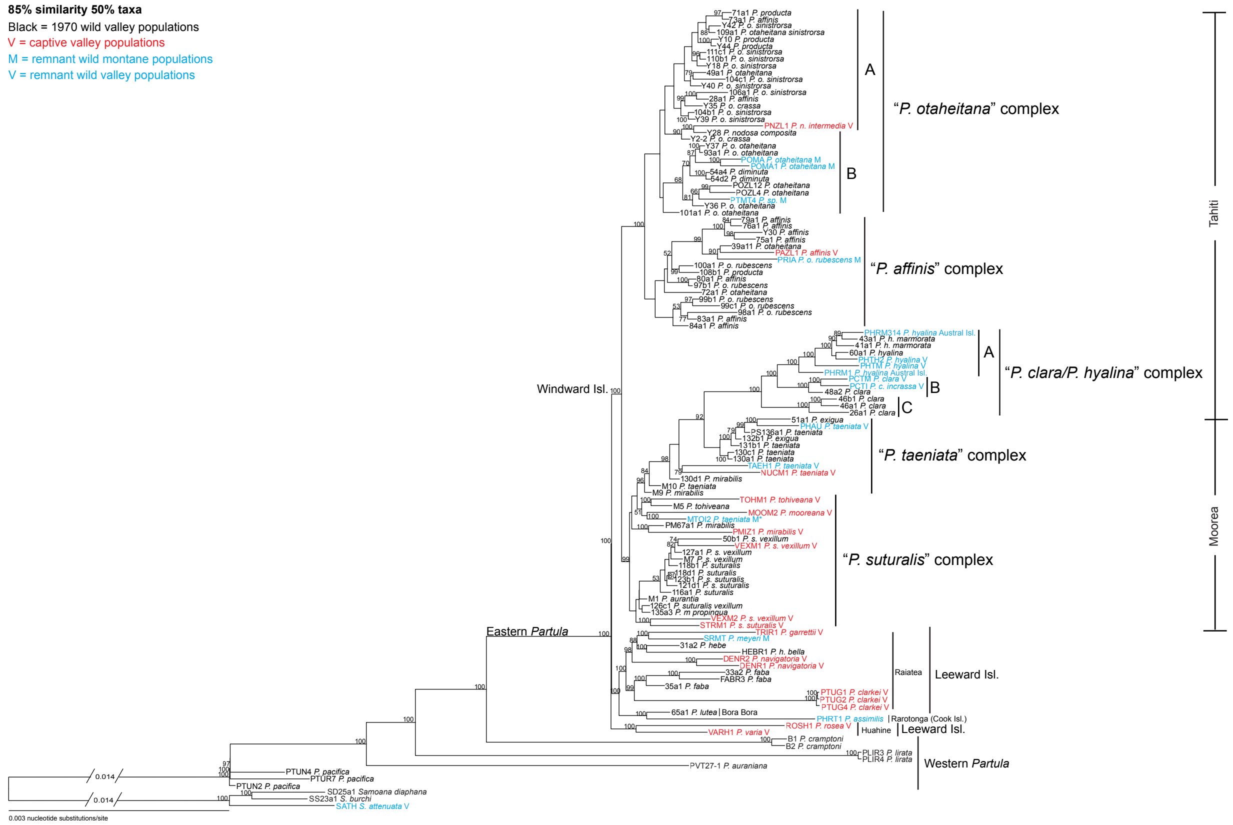
**85% similarity 50% taxa**
Black = 1970 wild valley populations
V = captive valley populations
M = remnant wild montane populations
V = remnant wild valley populations
"*P. otaheitana*" complex
A
B
"*P. affinis*" complex
"*P. clara/P. hyalina*" complex
C
"*P. taeniata*" complex
"*P. suturalis*" complex
Tahiti
Moorea
Windward Isl.
Eastern *Partula*
Raiatea
Leeward Isl.
Rarotonga (Cook Isl.)
Huahine
Western *Partula*
0.014
0.003 nucleotide substitutions/site

# Figure S1 (g)

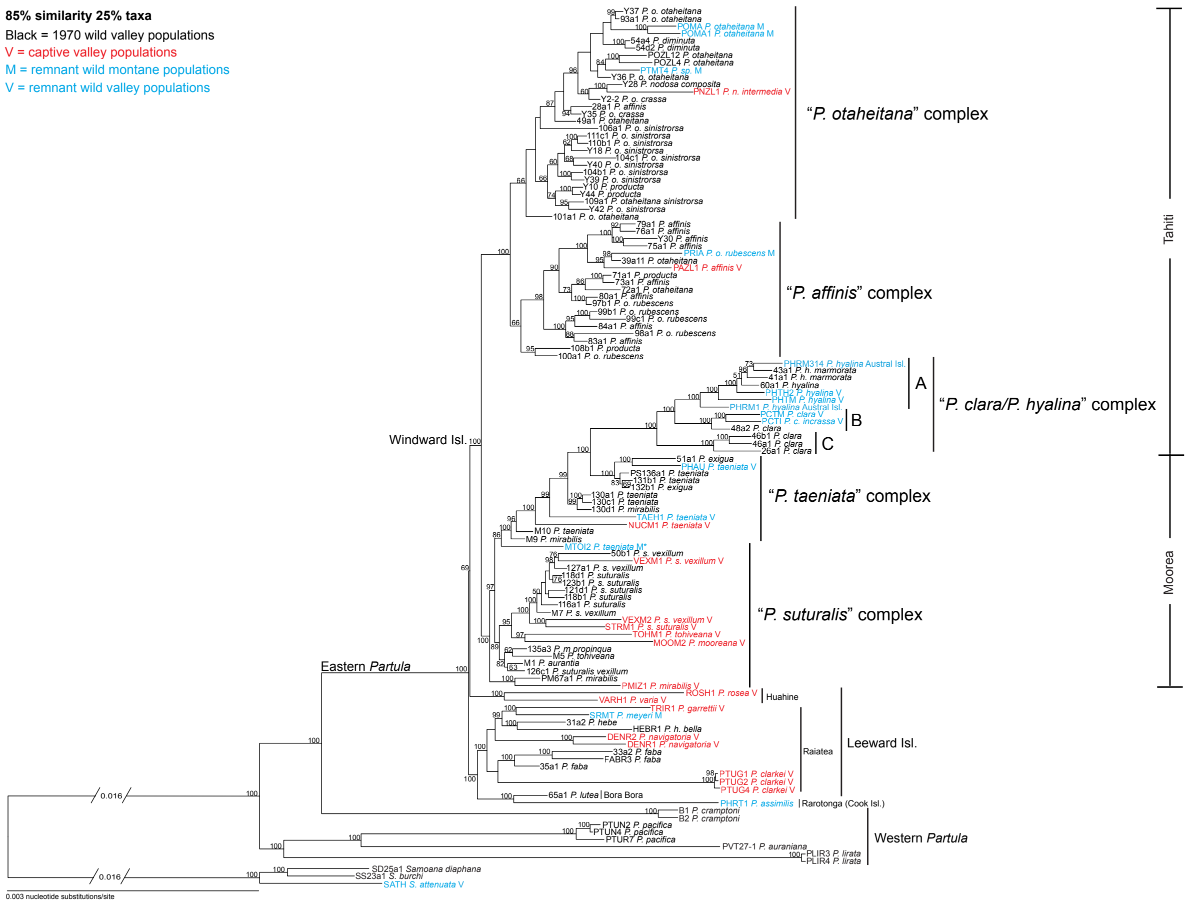

85% similarity 25% taxa
Black = 1970 wild valley populations
V = captive valley populations
M = remnant wild montane populations
V = remnant wild valley populations
"P. otaheitana" complex
"P. affinis" complex
"P. clara/P. hyalina" complex
"P. taeniata" complex
"P. suturalis" complex
Tahiti
Moorea
Windward Isl.
Eastern Partula
Huahine
Raiatea
Leeward Isl.
Bora Bora
Rarotonga (Cook Isl.)
Western Partula
0.003 nucleotide substitutions/site

# Figure S1 (h)

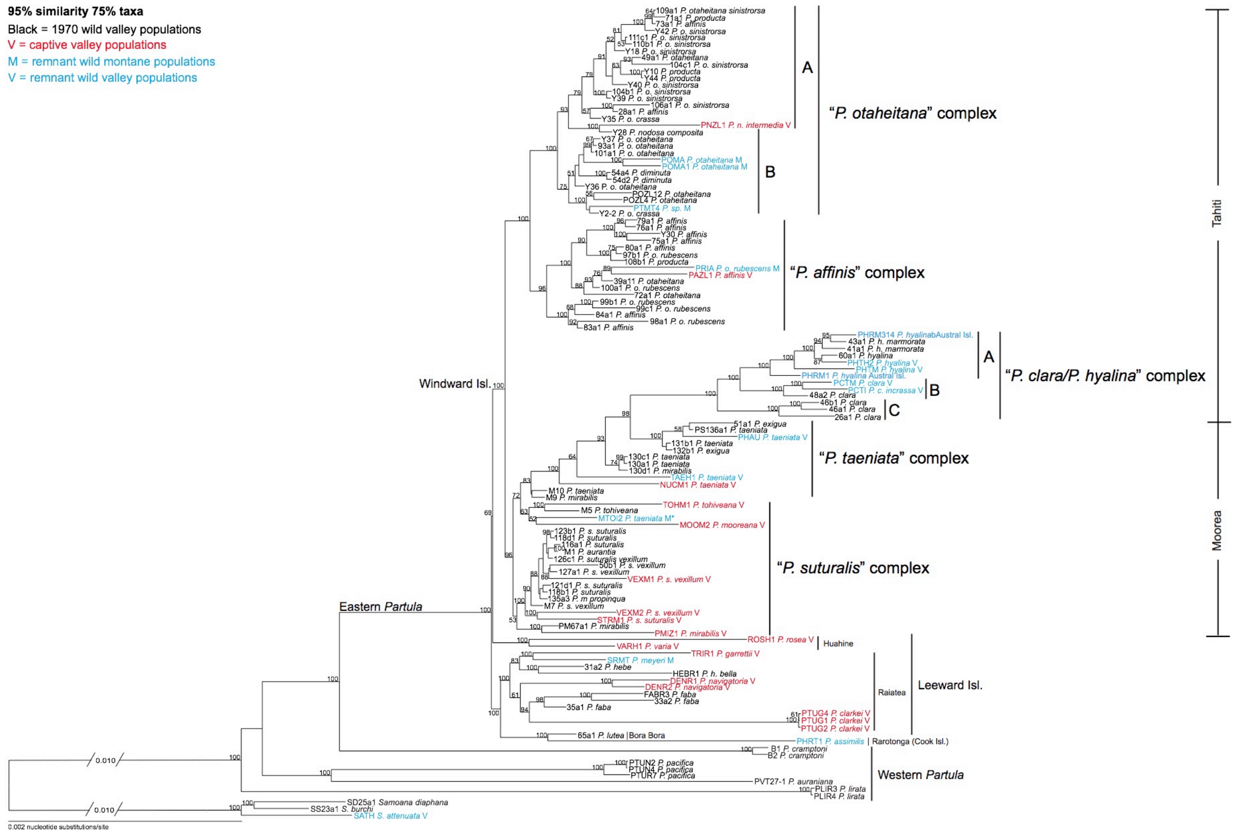
95% similarity 75% taxa
Black = 1970 wild valley populations
V = captive valley populations
M = remnant wild montane populations
V = remnant wild valley populations
"*P. otaheitana*" complex
A
B
"*P. affinis*" complex
"*P. clara/P. hyalina*" complex
"*P. taeniata*" complex
"*P. suturalis*" complex
C
Tahiti
Moorea
Windward Isl.
Eastern *Partula*
Huahine
Raiatea
Leeward Isl.
Bora Bora
Rarotonga (Cook Isl.)
Western *Partula*
0.010
0.002 nucleotide substitutions/site

# Figure S1 (i)

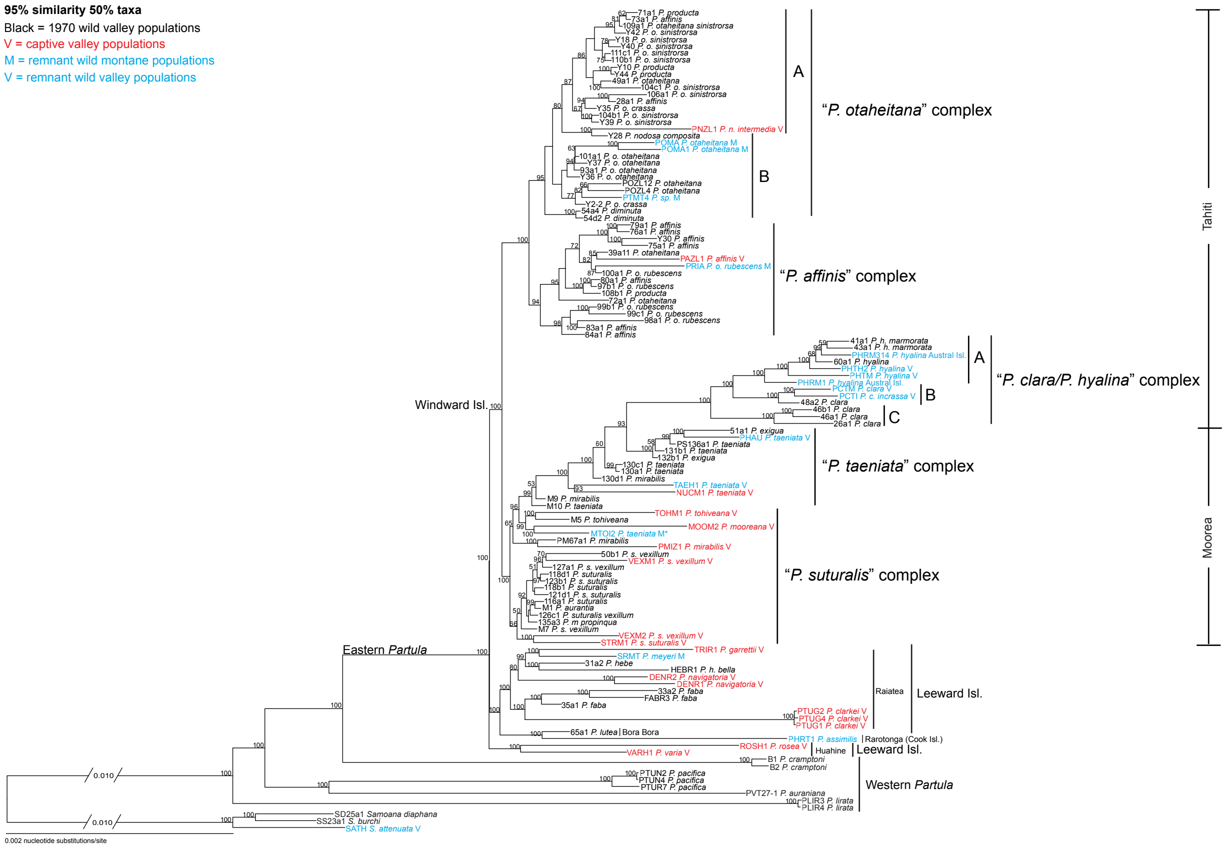
95% similarity 50% taxa
Black = 1970 wild valley populations
V = captive valley populations
M = remnant wild montane populations
V = remnant wild valley populations
"P. otaheitana" complex
A
B
"P. affinis" complex
"P. clara/P. hyalina" complex
C
"P. taeniata" complex
"P. suturalis" complex
Tahiti
Moorea
Windward Isl.
Eastern Partula
Raiatea
Leeward Isl.
Bora Bora
Rarotonga (Cook Isl.)
Huahine
Western Partula
0.010
0.002 nucleotide substitutions/site

## Figure S1 (j)

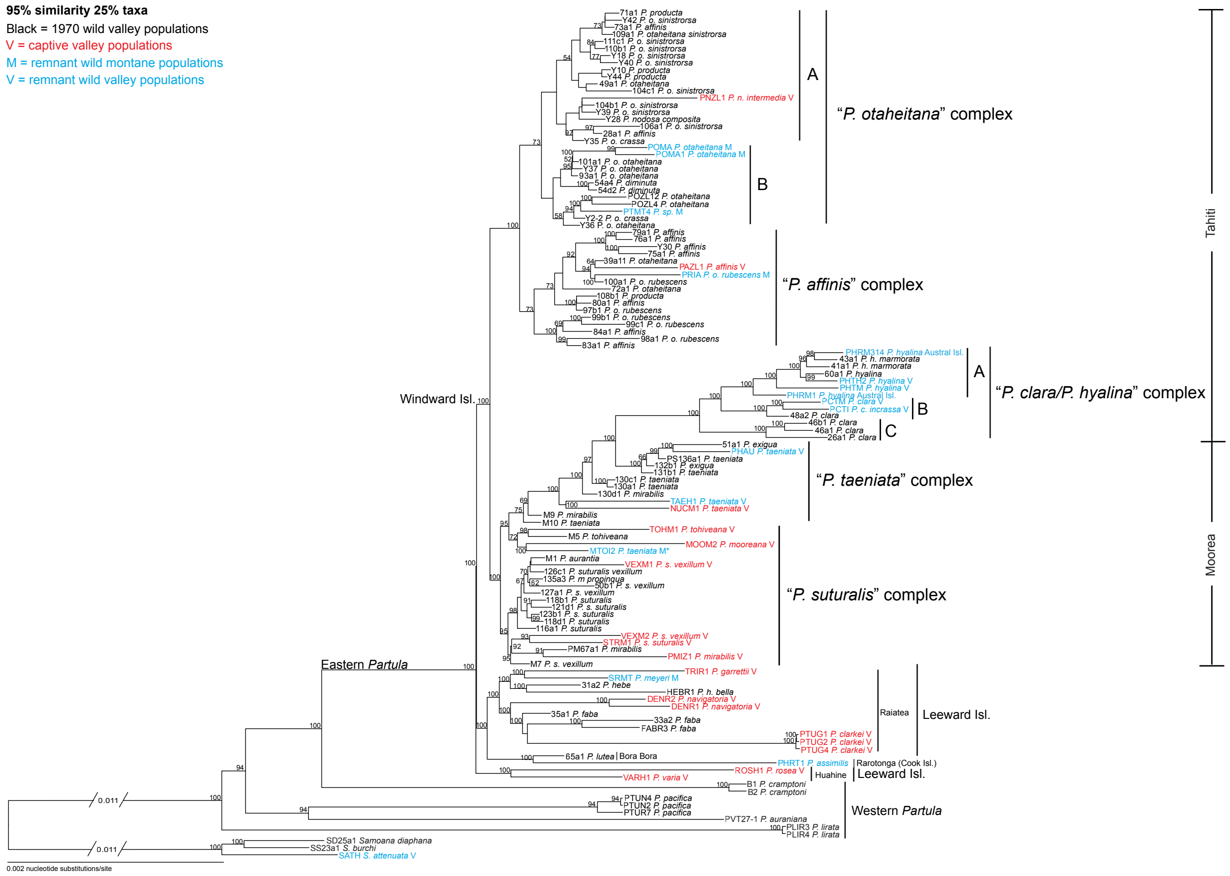


**Figure S1** Bayesian and Maximum likelihood phylogenomic trees depicting relationships among Society Island partulid species. Individuals were clustered at three different similarity thresholds (85, 90, and 95%) and minimum taxon coverages (75, 50, and 25%) and include the 90% similarity threshold, 75% of taxa (a) maximum likelihood and (b) Bayesian trees, totaling 2,169 loci and the Maximum likelihood (c) 90%, 50%, 11,026 loci, (d) 90%, 25%, 23,195 loci, (e) 85%, 75%, 1,607 loci, (f) 85%, 50%, 8,381 loci, (g) 85%, 25%, 18,154 loci, (h) 95%, 75%, 2,455 loci, (i) 95%, 50%, 11,506 loci, and (j) 95%, 25%, 28,194 loci trees. Trees were rooted with three species of Society Islands *Samoana,* the sister genus of *Partula.* Values on tree nodes indicate Bayesian posterior probabilities or Maximum likelihood bootstrap supports. Individuals are identified as 1970 wild (black), remnant valley (V, blue) or montane (M, blue), or captive (C, red) populations. * denotes the remnant montane *P. taeniata* individual (MTOI2) from Lineage 3 (see Discussion).

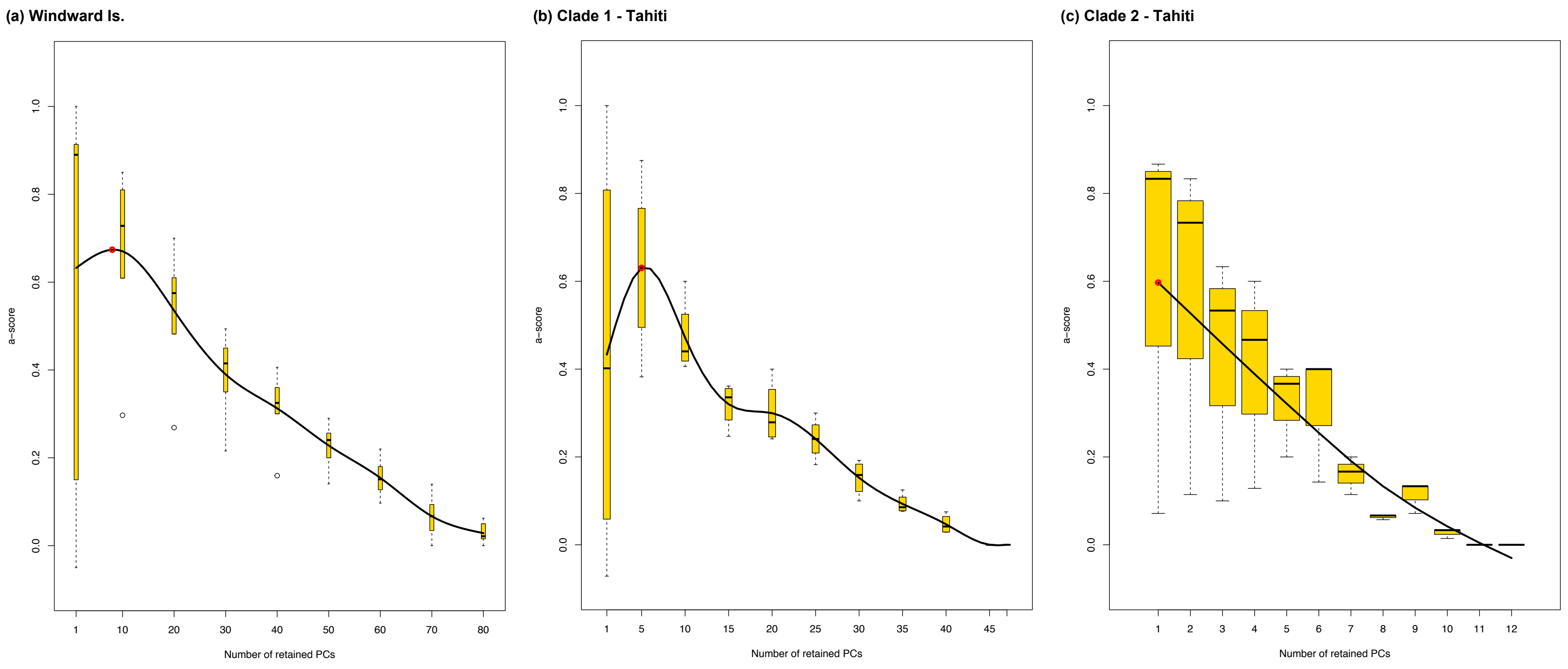

(a) Windward Is.
(b) Clade 1 - Tahiti
(c) Clade 2 - Tahiti
a−score
0.0
0.2
0.4
0.6
0.8
1.0
Number of retained PCs
1
10
20
30
40
50
60
70
80
5
15
25
35
45
2
3
4
6
7
8
9
11
12

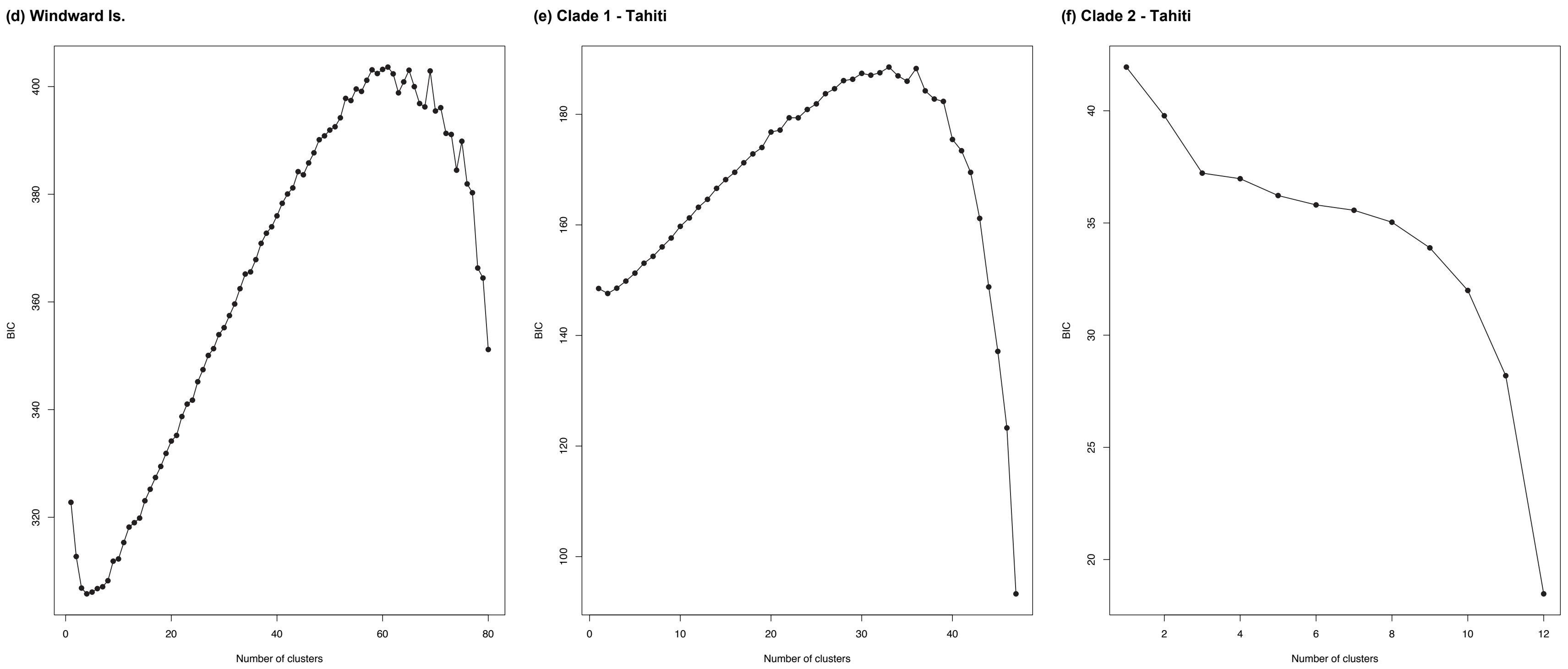


**Figure S2** Scatter plots showing the optimal number of principal components (a-c) and Bayesian Information Criterion (BIC) plots (d-f) used to determine the most likely number of clusters in the dataset for the Discriminant Analysis of Principal Components (DAPC; Jombart, Devillard, & Balloux, 2010) for (a, d) all 93 Moorean and Tahitian individuals analyzed, (b, e) Tahitian Clade 1 ("*P. otaheitana"* and *"P. affinis"* species complexes), and (c, f) the Tahitian portion of Clade 2 ("*P. clara/P. hyalina*" species complex).

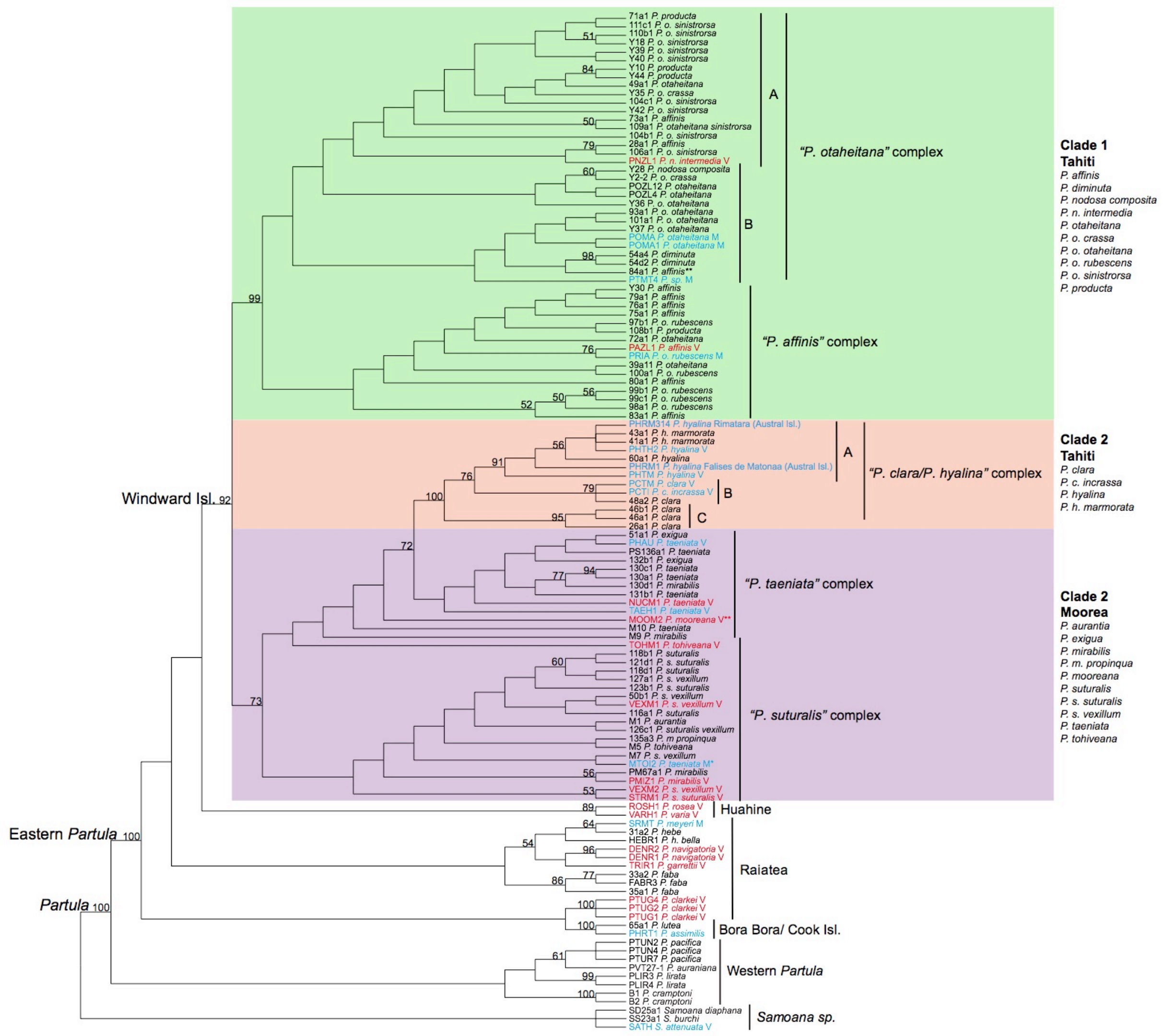


**Figure S3** Species tree estimation of Moorean and Tahitian *Partula* clades for each individual (see Figure 4 for grouped analysis). The 2,169 locus 90% similarity threshold clustering across 75% of individuals dataset was analyzed with SVDquartets (Chifman & Kubatko, 2014) as implemented in PAUP* (Swofford, 2002). Bootstrap supports are indicated for nodes with values > 50%. Tree was rooted with three species of Society Islands *Samoana* (denoted *Samoana* sp.)*,* the sister genus of *Partula.* All Society Islands *Partula* individuals were included in the analysis and are identified by their island of origin.

**Figure S4 (a)** All 93 Moorean and Tahitian *Partula* individuals. Figure caption can be found on page 41.

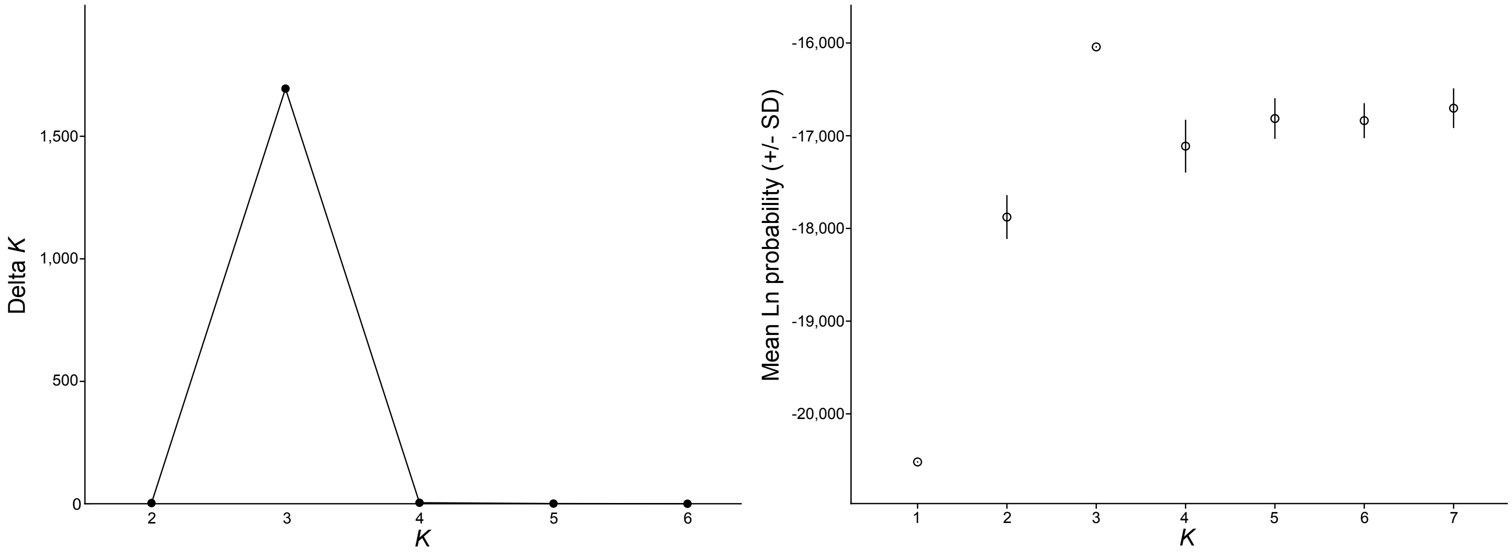


**(b)** Clade 1 – *"P. otaheitana*" and *"P. affinis*" species complexes

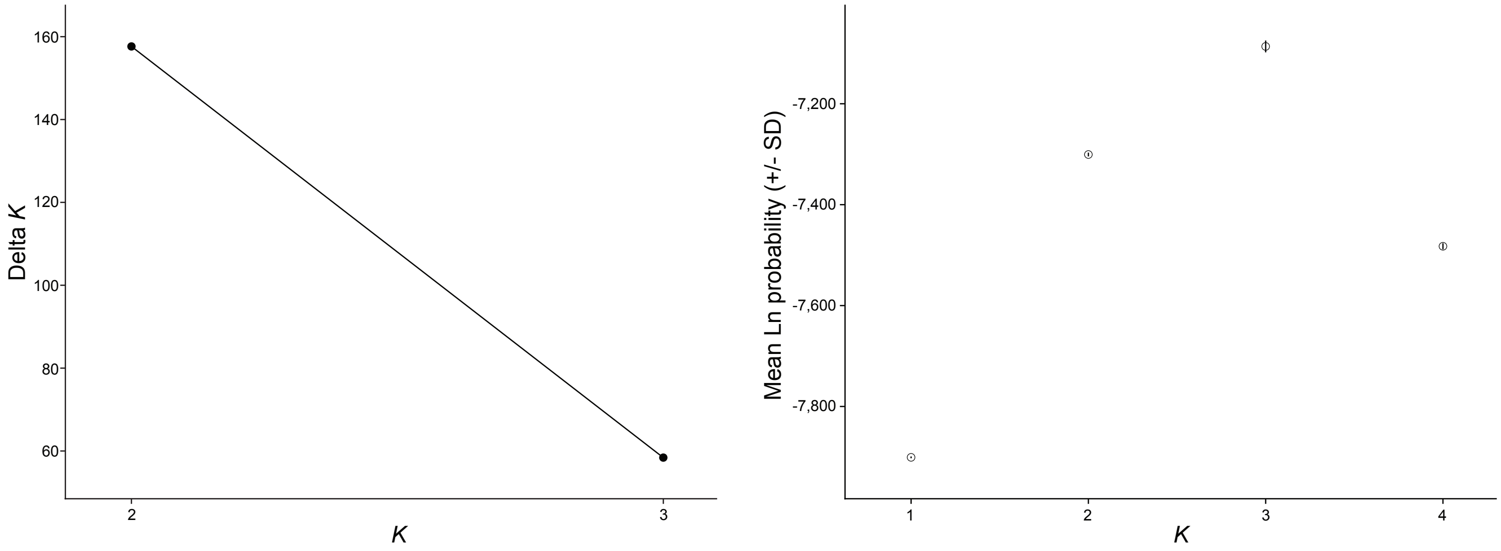

**Figure S4 (c)** Clade 2 – Moorea and Tahiti

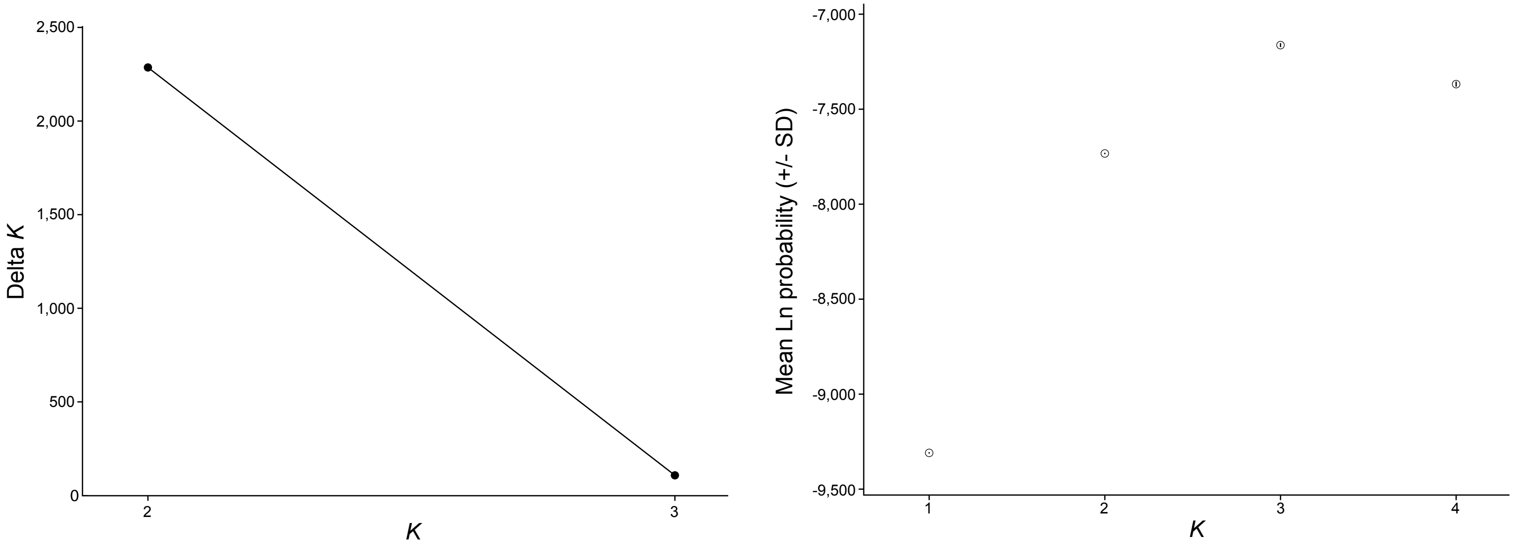


**(d)** Clade 2 – Tahiti – "*P. clara/P. hyalina"* species complex

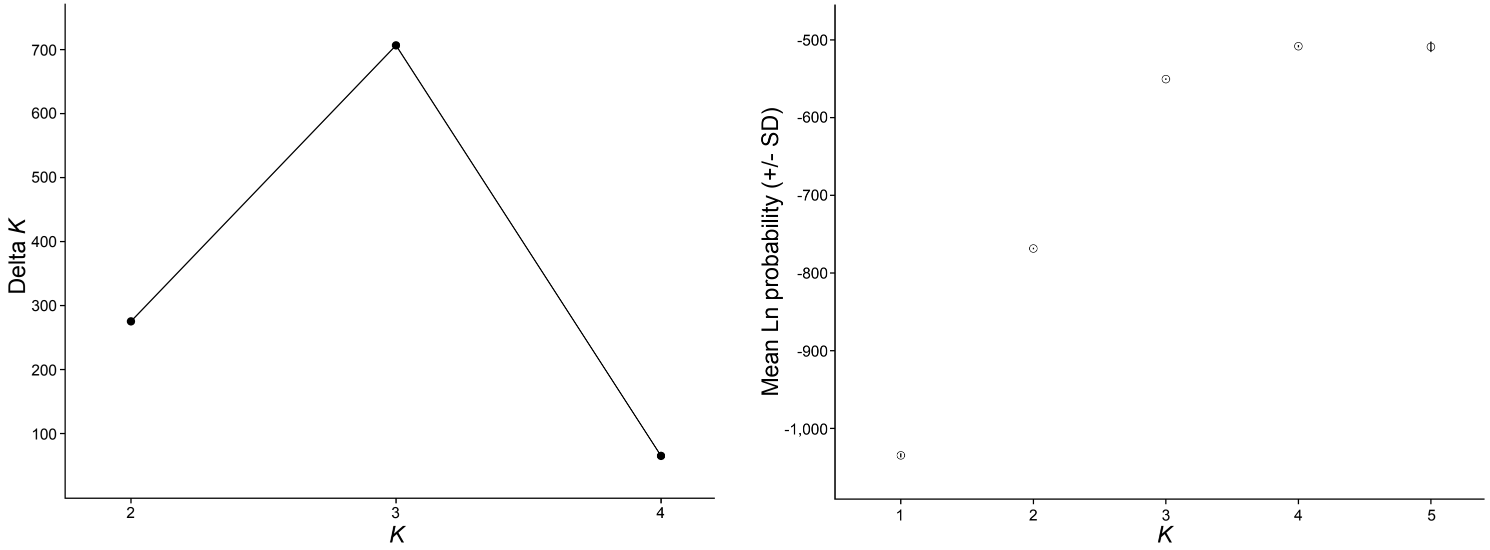

**(e)** Clade 2 – Moorea – *"P. taeniata*" and *"P. suturalis*" species complexes

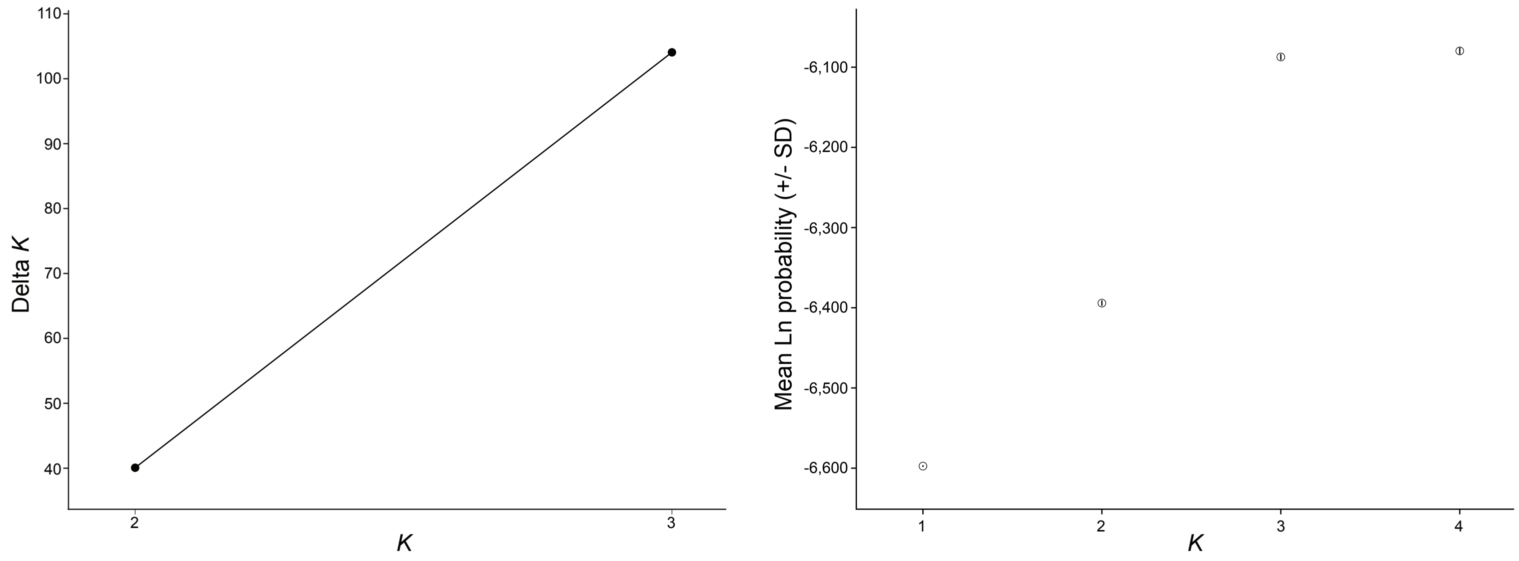


**Figure S4**. Scatter plots showing the most likely *K* for (a) all 93 Moorean and Tahitian individuals, (b) Clade 1 – Tahiti ("*P. otaheitana*" and "*P. affinis*" species complexes), (c) Clade 2 – Moorea and Tahiti, (d) Clade 2 – Tahiti (*"P. clara/P. hyalina*" species complex), and (e) Clade 2 – Moorea ("*P. taeniata*" and "*P. suturalis*" species complexes) using the Δ*K* method of Evanno, Regnaut, & Goudet (2005) and the mean natural log of the probability (+/- standard deviation (SD)) in Structure Harvester (Earl & vonHoldt, 2012). See Figure 5 for Structure bar graphs.

**Supporting Information References**

Chifman, J., & Kubatko, L. (2014) Quartet inference from SNP data under the coalescent model. *Bioinformatics, 30*, 3317-3324.

Earl, D. A., & vonHoldt, B. M. (2012) STRUCTURE HARVESTER: A website and program for visualizing STRUCTURE output and implementing the Evanno method. *Conservation Genetics Resources, 4*, 359-361.

Evanno, G., Regnaut, S., & Goudet, J. (2005) Detecting the number of clusters of individuals using the software Structure: A simulation study. *Molecular Ecology, 14*, 2611-2620.

Jombart, T., Devillard, S., & Balloux, F. (2010) Discriminant analysis of principal components: A new method for the analysis of genetically structured populations. *BMC Genetics, 11*, 94.

Pickrell, J. K., & Pritchard J. K. (2012) Inference of population splits and mixtures from genome-wide allele frequency data. *PLOS Genetics, 8*, e1002967.

Reich, D., Thangaraj, K., Patterson, N., Price, A. L., & Singh, L. (2009) Reconstructing Indian population history. *Nature, 461*, 489-495.

Swofford, D. L. (2002) PAUP*. Phylogenetic Analysis Using Parsimony (*and Other Methods). Version 4. Sunderland, MA: Sinauer Associates.
